# Modeling CICR in rat ventricular myocytes: voltage clamp studies

**DOI:** 10.1186/1742-4682-7-43

**Published:** 2010-11-10

**Authors:** Abhilash Krishna, Liang Sun, Miguel Valderrábano, Philip T Palade, John W Clark

**Affiliations:** 1 Department of Electrical and Computer Engineering, Rice University, 6100 Main Street, Houston, 77005, USA; 2 Proportional Technologies, Inc., 8022 El Rio Street, Houston, 77054, USA; 3 Methodist Hospital Research Institute, Methodist DeBakey Heart & Vascular Center, 6565 Fannin Street, Houston, 77030, USA; 4 Department of Pharmacology and Toxicology, University of Arkansas for Medical Sciences, 4301 West Markham Street, Little Rock, 72205, USA

## Abstract

**Background:**

The past thirty-five years have seen an intense search for the molecular mechanisms underlying calcium-induced calcium-release (CICR) in cardiac myocytes, with voltage clamp (VC) studies being the leading tool employed. Several VC protocols including lowering of extracellular calcium to affect *Ca*^2+ ^loading of the sarcoplasmic reticulum (SR), and administration of blockers caffeine and thapsigargin have been utilized to probe the phenomena surrounding SR *Ca*^2+ ^release. Here, we develop a deterministic mathematical model of a rat ventricular myocyte under VC conditions, to better understand mechanisms underlying the response of an isolated cell to calcium perturbation. Motivation for the study was to pinpoint key control variables influencing CICR and examine the role of CICR in the context of a physiological control system regulating cytosolic *Ca*^2+ ^concentration ([*Ca*^2+^]*_myo_*).

**Methods:**

The cell model consists of an electrical-equivalent model for the cell membrane and a fluid-compartment model describing the flux of ionic species between the extracellular and several intracellular compartments (cell cytosol, SR and the dyadic coupling unit (DCU), in which resides the mechanistic basis of CICR). The DCU is described as a controller-actuator mechanism, internally stabilized by negative feedback control of the unit's two diametrically-opposed *Ca*^2+ ^channels (trigger-channel and release-channel). It releases *Ca*^2+ ^flux into the cyto-plasm and is in turn enclosed within a negative feedback loop involving the SERCA pump, regulating[*Ca*^2+^]*_myo_*.

**Results:**

Our model reproduces measured VC data published by several laboratories, and generates graded *Ca*^2+ ^release at high *Ca*^2+ ^gain in a homeostatically-controlled environment where [*Ca*^2+^]*_myo _*is precisely regulated. We elucidate the importance of the DCU elements in this process, particularly the role of the ryanodine receptor in controlling SR *Ca*^2+ ^release, its activation by trigger *Ca*^2+^, and its refractory characteristics mediated by the luminal SR *Ca*^2+ ^sensor. Proper functioning of the DCU, sodium-calcium exchangers and SERCA pump are important in achieving negative feedback control and hence *Ca*^2+ ^homeostasis.

**Conclusions:**

We examine the role of the above *Ca*^2+ ^regulating mechanisms in handling various types of induced disturbances in *Ca*^2+ ^levels by quantifying cellular *Ca*^2+ ^balance. Our model provides biophysically-based explanations of phenomena associated with CICR generating useful and testable hypotheses.

## Background

Contraction of cardiac muscle is triggered by a transient rise in intracellular *Ca*^2+ ^concentration [*Ca*^2+^]*_myo_*. Sarcolemmal (SL) membrane depolarization triggers *Ca*^2+ ^influx from the extracellular medium by opening dihydropyridine (DHP)-sensitive L-type *Ca*^2+ ^channels. Following diffusion across a small sub-membrane dyadic space, this influx activates ryanodine receptors (RyRs) controlling ryanodine-sensitive *Ca*^2+ ^release channels in the junctional portion of the sarcoplasmic reticulum (jSR). Fabiato and Fabiato [1] named the process calcium-induced calcium release (CICR). *Ca*^2+ ^subsequently diffuses from the dyadic space into the cytosol. Ultimately, intracellular *Ca*^2+ ^concentration [*Ca*^2+^]*_myo _*is returned to resting levels by combination of: (a) *Ca*^2+ ^buffering in the dyadic space and cytosol; (b) sequestration of *Ca*^2+ ^by sarcoplasmic/endoplasmic reticulum *Ca*^2+^-ATPase (SERCA)-type calcium pumps lining the longitudinal portion of the sarcoplasmic reticulum (LSR); and (c) *Ca*^2+ ^extrusion from the cytosol by *Na*^+^/*Ca*^2+ ^exchangers and *Ca*^2+^-ATPase pumps on the sarcolemmal membrane.

CICR in cardiac muscle exhibits both graded behavior and a high gain. Graded behavior refers to the obser-vation that SR *Ca*^2+ ^release is proportional to the influx of trigger *Ca*^2+ ^[2], whereas high gain indicates that the SL trigger current elicits a high SR *Ca*^2+ ^release flux. Graded *Ca*^2+ ^release with high gain is somewhat paradoxical according to Stern [3], in that the positive feedback inherent in such high-gain systems tend to produce regenerative, nearly all-or-none release rather than graded release. Several deterministic models have been developed to explain excitation-contraction (E-C) coupling [4,5], but none of them can explain the mechanism of graded release at high gain over a wide range of values for sarcolemmal *Ca*^2+ ^current. Stern [3] proposed that such a gradation paradox might be explained if the stimulus for *Ca*^2+ ^release by RyRs were actually the local nanodomains of [*Ca*^2+^] generated by nearby L-type channels, rather than the global cytosolic [*Ca*^2+^]*_myo_*. According to this hypothesis, graded control of macroscopic SR *Ca*^2+ ^release can be achieved by graded statistical recruitment of individual, autonomous, all-or-none stochastic release events [6]. In these studies, a distributed differential model of high order that included dynamic interactions between large numbers of individual channels was used to demonstrate this concept. However, rather large amounts of computation time are required with distributed stochastic models of this type. Additional models have sought to characterize the *Ca*^2+ ^release complex, including several [7-9] based on the stochastic release process adopted by Stern et al. These statistical models have solved the graded release problem, however, they too are complicated and computationally very expensive. Other models based on the simplified local control model of CICR developed by Hinch et al. [10] sought to adopt a lower order description of the E-C coupling process [11,12] by making an approximation of rapid equilibrium in the dyadic space. The latency from onset of *Ca*^2+ ^entry via the *I_Ca,L _*channel to triggered SR *Ca*^2+ ^release is known to increase with decrease in the magnitude of *I_Ca,L,TT _*[13], the modeling of which is made possible by considering *Ca*^2+ ^diffusion in the dyadic medium. These models [11,12] also approximate the SR as a single volume compartment with no distinction between junctional versus the longitudinal (network) SR compartments. However, recent work [14] points towards the important role of the *Ca*^2+ ^refilling rate from the network to junctional SR in controlling RyR release termination via the luminal sensor. Shiferaw et al. [15] developed a computationally tractable model of *Ca*^2+ ^cycling to represent the release of calcium from the SR as a sum of spatially localized events that correspond to *Ca*^2+ ^sparks, assuming the recruitment rate of *Ca*^2+ ^sparks is directly proportional to the whole-cell *I_Ca,L _*current. This assumption overlooks the complex calmodulin mediated interaction (calcium dependent facilitation (CDF) and calcium dependent inactivation (CDI)) of the *I_Ca,L _*channel with calcium in its vicinity. It also demands a large amount of computation.

Numerically, distributed as well as statistical models tend to be computationally expensive due to the in-herent repetition involved in the computation. In a spatially distributed model, simultaneous solution for dynamics in identical compartments distributed in space would amount to a large computational cost. In statistical models inference is drawn based on multiple runs of identical events which translate into a prolonged simulation time. Hence, these models are cumbersome to implement, particularly in larger multiple-cell simulations. Consequently, we consider a deterministic approach to the characterization of CICR. Specifically, we develop a lumped model of the *Ca*^2+ ^release complex that includes: (a) a sub-sarcolemmal dyadic cleft space separating the SL and jSR membranes; (b) a single DHP-sensitive *Ca*^2+ ^channel on the SL membrane; and (c) a single equivalent Ry-sensitive channel arranged symmetrically on the opposing jSR membrane that represents the output of a local cluster of Ry-sensitive channels facing the DHP-sensitive channel. Based on morphological data compiled by Bers [16], we further assume that each ventricular cell contains 10,000 of these dyadic *Ca*^2+ ^release units, and that they are associated with the fraction of the SL membrane that is coupled with the jSR. That is, we partition the sarcolemma into free and dyadically coupled SL membrane, and associate each with a different fluid compartment: the cell cytosolic medium in the case of the free SL membrane, and the dyadic cleft space medium in the case of the dyadic-coupled fraction. In a sense, we build on Stern's [3] local domain concept by considering the aforementioned local nanodomains identical, but focusing on the nonlinear dynamics of the two different types of *Ca*^2+ ^channel in the dyadic coupling unit. Our deterministic model although is very descriptive, is computationally tractable and has a run time of 21 sec (including recording of 73 variables of type double on a data file) for 1 cycle of 4 Hz voltage clamp stimulation.

## Methods

### Experimental Methods

Rat ventricular myocytes were prepared from 200-300 g male Sprague Dawley rats by dissociation with collagenase, as previously described [17]. All experiments were performed under conventional whole cell recording conditions with a List EPC-7 patch clamp, recording fluorescence from nearly the entire cell, as described by Fan and Palade [17]. Recordings from an individual cell were rarely extended beyond 10 min in order to reduce as much as possible both escape of dye from the cell and *Ca*^2+ ^current rundown. External solution in the bath was normal Tyrode (1 mM *Ca*^2+^) with *Cs*^+ ^substituted for *K*^+ ^for purposes of blocking inward rectifier *K*^+ ^currents. The internal solution in the pipette contained Cs aspartate supplemented with 20 mM *CsCl*, 3 mM *Na_2_ATP *, 3.5 mM *MgCl_2 _*and 5 mM HEPES. Holding potential used was -40 mV.

### Computational Aspects

All simulations and analysis were performed on a 2.8 GHz Intel^® ^Core™2 Duo CPU-based computer using Microsoft Windows XP operating system. To find the parameters involved in the 6 state Markovian model for *I_Ca,L_*, a non-linear least-squares method [18] was used to obtain the solution of the system of non-linear ordinary differential equations. Specifically, we have employed an algorithm given by Lau [19]. The numerical integration scheme used to solve the full set of forty two 1st-order differential equations describing the dynamic model was the Merson-modified Runge-Kutta 4th-order method [20,21] with a conservative fixed time step, chosen small enough to allow the local truncation error to be of fourth order. The explicit finite difference scheme was used to numerically solve the Laplacian equations of *Ca*^2+ ^diffusion in the cleft space. Detailed numerical methods are similar to those presented by Smith et al. [22]. The results were visualized using Matlab by Mathworks and Origin by Microcal Software.

### Model Development

Our objective was to develop a model of the rat ventricular cell which could be used to explain *Ca*^2+ ^signaling at the nanoscale level of the dyad and integrate the contributions of many dyads to produce a *Ca*^2+ ^transient and continuous *Ca*^2+ ^balance at the whole-cell level. Therefore, we start with a broad discussion of the elements of the DCU and its *Ca*^2+ ^supply (the jSR), and continue with a progressively more detailed description of the whole cell model. It is important to note that all *Ca*^2+ ^concentrations discussed in the model pertain to unbound *Ca*^2+ ^unless specified.

### Membrane Classification

**Figure 1:**
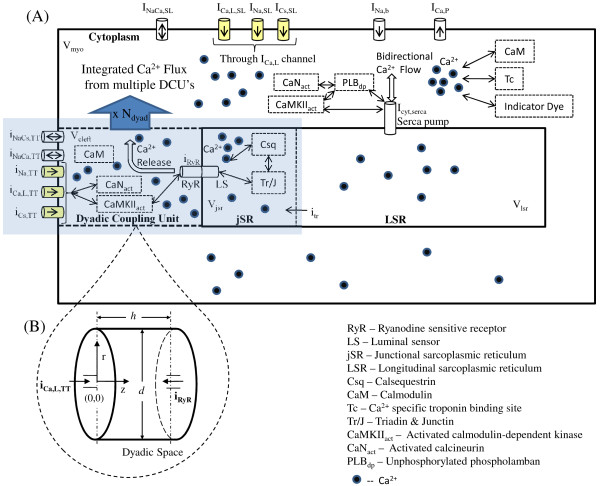
**Cellular fluid compartments**. Figure 1: Cellular fluid compartments. (a) Model configuration showing dyadic space, jSR, LSR, cytoplasm and SL; (b) Inset provides a more detailed description of the dyadic space showing the coupling of the two types of *Ca*^2+ ^channels (trigger and *Ca*^2+ ^release channels) via the dyadic fluid medium. Only one representative dyadic coupling unit is shown, however the whole model contains such 10,000 identical units lumped together.

**Figure 2:**
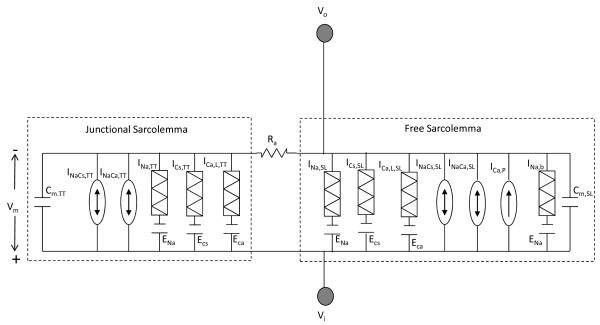
**Electrical equivalent circuit for the plasma membrane of a rat ventricular cell**. Figure 2: C_*m*,*TT *_: membrane capacitance of the junctional SL membrane coupled with the dyadic space; C_*m*,*SL*_: membrane capacitance of the uncoupled free SL membrane; Currents through the uncoupled free SL membrane are (a) I_*Ca*,*L*,*SL*_: L-type calcium current, (b) I_*Na*,*SL*_: sodium current through the DHPR channel, (c) I_*Cs*,*SL*_: cesium current through the DHPR channel, (d) *I*_*NaC*a,*SL*_: sodium-calcium exchanger current, (e) I_*NaCs*,*SL*_: sodium-cesium exchanger current, (f) I*_PMCA_*: calcium pump current, (g) I_*Na*,*b *_: background sodium current; Currents through the junctional SL membrane coupled with a dyadic space are (h) I_*Ca*,*L*,*TT *_: L-type calcium current; (i) I_*Na*,*TT *_: sodium current through the DHPR channel, (j) I_*Cs*,*TT *_: cesium current through the DHPR channel, (k) I_*NaCa*,*TT *_: sodium-calcium exchanger current, (l) I_*NaCs*,*TT *_: sodium-cesium exchanger current; V*_o_*: potential in external medium; V*_i_*: intracellular potential; The coupling resistance between the surface sarcolemma and the transverse tubules being very small is neglected in our model hence V*_m_*: common transmembrane potential across both uncoupled and coupled membranes.

**Figure 3:**
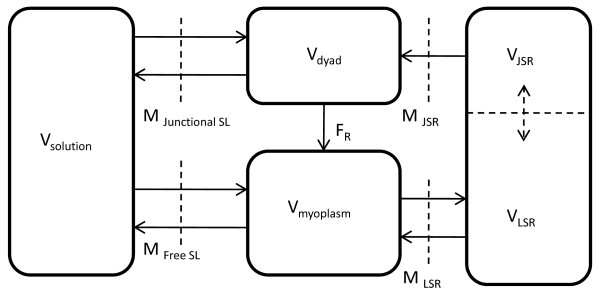
**Fluid compartment model**. Figure 3: Representative fluid compartment model, showing membrane surface area separating different compartments. M*_FreeSL_*: free SL membrane; M*_JunctionalSL_*: junctional SL membrane; M*_jSR_*: junctional SR membrane; M*_LSR_*: Longitudinal SR membrane.

We assume that a continuous membrane barrier exists between the cytoplasm and the external bathing medium (Figure 1A; Figure 2), which consists of two components: a surface sarcolemma (SL) (*M_FreeSL _*in Figure 3) free of any sub-membrane contact with the junctional sarcoplasmic reticulum (jSR) and the remnant membrane (*M_JunctionalSL _*in Figure 3) that does make contact with the jSR via a dyadic space (nanodomain) (Figure 1A). These membrane components have the same basic plasma membrane, but differ in content with regard to total membrane surface area, type and distribution of transmembrane ion channels, ATPase pumps and exchangers, as well as their functional coupling with a dyadic space. Ultrastructural information from several cardiac preparations including the rat ventricular cell has been compiled by Bers [16], which can be used to estimate the percentage of the cell membrane in contact with a dyadic space, for either the free surface plasmalemma or for the transverse tubule (TT) which brings the extracellular medium to the plasma membrane of the dyadic coupling unit. Thus, the bounding membrane is divided into two lumped parts (free and coupled) based on the existence of sub-membrane coupling to a dyadic space (Table 1). A portion of membrane could be part of a transverse tubular membrane, but if there is no dyadic coupling involved, that membrane would be classified as belonging to the free surface plasmalemma. Another portion of membrane might be part of the bounding outer surface of the cylindrical cell and yet have submembrane coupling to a dyadic space. In this case, it would be classified as belonging to the coupled category. Table 2 gives values for volumes of the fluid compartments (shown in Figure 3) assumed for the rat ventricular cell, which are largely based on measured data from rat ventricular myocytes [16].

**Table 1:** Surface area of various plasma membranes in the cell

Variable	Description	Value
*A_Ext.SL_*	Surface area of external SL	11.4 × 10^3 ^*μm*^2^

*A_TT_*	Surface area of T-tubule	5.52 × 10^3 ^*μm*^2^

*A_TotSL_*	Surface area of total SL (including external SL and T-tubule)	16.9 × 10^3 ^*μm*^2 ^(*)

*A_JunctExt.SL_*	Surface area of junctional external SL	0.846 × 10^3 ^*μm*^2^

*A_JunctTT_*	Surface area of junctional T-tubule	2.54 × 10^3 ^*μm*^2^

*A_TotJunct_*	Surface area of total junctional plasma membrane	3.39 × 10^3 ^*μm*^2^

*A_JunctSR_*	Surface area of junctional SR	6.99 × 10^3 ^*μm*^2^

*A_LongSR_*	Surface area of longitudinal SR	36.8 × 10^3 ^*μm*^2^

*A_TotSR_*	Surface area of total SR	43.8 × 10^3 ^*μm*^2^

*Electrical capacitance of the cell membrane = 169 pF (using 1 *μ*F/*cm*^2^)

Table 1: Surface area of various plasma membranes in the cell. All the above parameters are derived from Bers [16].

**Table 2:** Parameters used to model sub-cellular morphology

Parameter	Definition	Value	References
*N_dyad_*	Number of dyadic units	10000	[8]^‡^

*V_myo_*	Myoplasmic volume	5.3581 × 10^-2 ^*nL*	[16]^†^

*V_LSR_*	Longitudinal SR volume	1.1776 × 10^-3 ^*nL*	[16]*

$\sum_{N\;dyad}V_{jSR}$	Total junctional SR volume	1.104 × 10^-4 ^*nL*	[16]*

Δ*r*	Step size in the 'r' direction	10 *nm*	Numerical solution^†^

*d*	Diameter of the cylindrical cleft space in the 'r' direction	400 *nm*	[119,8,121,6]^‡^

Δ*z*	Step size in the 'z' direction	0.76 *nm*	Numerical solution^†^

*h*	Length of the cylindrical cleft space in the 'z' direction	15.2 *nm*	[119,8,121,6]^‡^

*V_cleft_*	Volume of a unit dyadic space	1.91 × 10^-9 ^*nL*	--

Table 2: Parameters used to model sub-cellular morphology. Adopted (*), derived (†) or estimated (‡) from the cited sources.

### Channel and Exchanger Distribution

**Figure 4:**
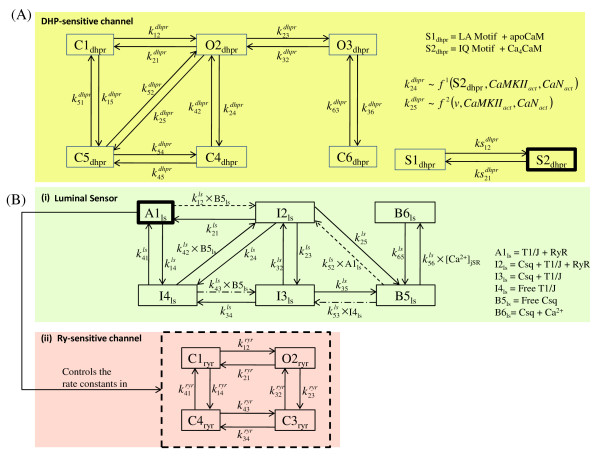
**Calcium channel dynamics**. Figure 4: Calcium channel dynamics. (a) Markovian model describing the DHP-sensitive *Ca*^2+ ^channel, and (b) Markovian model of the Ry-sensitive *Ca*^2+ ^channel and the luminal SR *Ca*^2+ ^sensor. Input from the luminal SR *Ca*^2+ ^sensor modulates the rate constants in the model of Ry-sensitive channel exercising the indirect bias of luminal [*Ca*^2+^]*_jSR _*on the RyR receptor.

Recent research has also shown that besides L-type *Ca*^2+ ^channels, *Na*^+^/*Ca*^+ ^exchanger activity is also found predominantly in the T-tubules of rat ventricular myocytes [23]. Our model configuration reflects this finding in that the tubular fraction of *I_Ca,L _*,*I_NaCa _*and *I_NaCs _*channels facing a unitary dyadic space are denoted as *i_Ca,L,TT _*(source of trigger *Ca*^2+ ^into a unitary dyad), *i_NaCa,TT _*and *i_NaCs,TT _*respectively (Figure 2). The free sarcolemmal component of these same channels are denoted as *I_Ca,L,SL_*, *I_NaCa,SL _*and *I_NaCs,SL _*respectively. *I_Ca,L,TT _*is the total current entering through L-type *Ca*^2+ ^channels via all the dyadic units (*N_dyad_*). We define the total L-type current *I_Ca,L _*as the combination of *I_Ca,L,TT _*and *I_Ca,L,SL _*(i.e., *I_Ca,L _*= *I_Ca,L,TT _*+ *I_Ca,L,SL_*). *I_Ca,L _*in our model is mostly (90%) from the L-type *Ca*^2+ ^current in the T-tubules, since Kawai et al. [24], found L-type current to be highly concentrated (9-fold) in the T-tubules (*I_Ca,L,TT_*) vs. the cell surface sarcolemma (*I_Ca,L,SL_*) of rat ventricular myocytes. We described the *I_Ca,L _*channel using a 6-state Markovian model as shown in Figure 4A. The distribution of *I_NaCa _*and *I_NaCs _*correspond to that of *I_Ca,L _*channel in order to ensure *Ca*^2+ ^and *Na*^+ ^ion balance.

Since our study is focused on voltage clamp testing of *Ca*^2+ ^transients in rat ventricular cells, we assume that the majority of *Na*^+ ^and *K*^+ ^channels are blocked by either the holding potential used (-40 mV) or appropriate blocking agents. Thus, these channels are not modeled, and we assume that only the dihy-dropyridine (DHP) -sensitive *Ca*^2+ ^channels, the electrogenic pumps, *Na*^+^/*Ca*^+ ^exchangers and *Na*^+^/*Cs*^+ ^pumps expressed in the free and/or coupled SL membranes contribute to the voltage clamp response. Table 3 provides values for various parameters used to model the ion transport across the sarcolemmal membrane.

**Table 3:** Parameters used to model ion transport across the sarcolemmal membrane

Parameter	Definition	Value	References
*F*	Faraday's constant	96485 *coul · mol*^-1^	--

*R*	Ideal gas constant	8314 *mJ · mol*^-1 ^*· K*^-1^	--

*T*	Absolute temperature	290 *K*	Measured

[*Ca^2+^*]*_o_*	Extracellular *Ca*^2+ ^concentration	1.0 *mM*	Measured

[*Na^+^*]*_o_*	Extracellular *Na*^+ ^concentration	140.0 *mM*	Measured

[*Cs^+^*]*_o_*	Extracellular *Cs*^+ ^concentration	3.0 *mM*	Measured

*Z_Na_*, *Z_Cs_*	Valence of *Na*^+ ^and *Cs*^+ ^ions	1.0	--

*Z_Ca_*, *Z_Ba_*	Valence of *Ca*^2+ ^and *Ba*^2+ ^ions	2.0	--

*P_Ca_*	Permeability of L-Type calcium channel to *Ca*^2+^	6.7367 × 10^-9 ^*μL · s*^-1^	[30]*

*P_Na_*	Permeability of L-Type calcium channel to *Na*^+^	8.0355 × 10^-11 ^*μL · s*^-1^	[30]*

*P_Cs_*	Permeability of L-Type calcium channel to *Cs*^+^	6.2088 × 10^-11 ^*μL · s*^-1^	[30]*

*K_mAllo_*	Dissociation constant for allosteric *Ca*^2+ ^activation	125 × 10^-6 ^*mM*	[85]*

*K_mCao_*	Dissociation constant for extracellular *Ca*^2+^	1.14 *mM*	[85]*

*K_mCai_*	Dissociation constant for intracellular *Ca*^2+^	0.0036 *mM*	[122]*

*K_mNao_*	Dissociation constant for extracellular *Na*^+^	87.5 *mM*	[83]*

*K_mNai_*	Dissociation constant for intracellular *Na*^+^	12.3 *mM*	[85]*

*V_max_*	Maximum *Na*^+^/*Ca*^+ ^exchange current	776.2392 *pA*	[85]*

*k_mpca_*	Half saturation constant for the SL *Ca*^2+ ^pump	0.5 *μM*	[30]*

${\bar{I}}_{PMCA}$	Maximum sarcolemmal *Ca*^2+ ^pump current	1.15 *pA*	[30]*

*K_mcs_*	Dissociation constant for extracellular *Cs*^+^	1.5 × 10^3 ^*μM*	[94,92,88,74]^‡^

*K_mna_*	Dissociation constant for intracellular *Na*^+^	2.14 × 10^5 ^*μM*	[94,92,88,74]^‡^

${\bar{I}}_{NaCs}$	Maximum *Na*^+^/*Cs*^+ ^pump current	147.3 *pA*	[94,92,88,74]^‡^

*G_Nab_*	Maximum background *Na*^+ ^current conductance	0.00141 *nS*	[30]*

*R_a_*	Mean access resistance of the tubular system	20.0 *kΩ*	[123]*

Table 3: Parameters used to model the transmembrane currents *I*_*Ca*,*L *_, *I_NaCa_*, *I_PMCA_*, *I_NaCs _*and the background sodium current I*_Na,b_*. Adopted (*) or estimated (‡) from the cited sources.

### The SR Fluid Compartment

**Figure 5:**
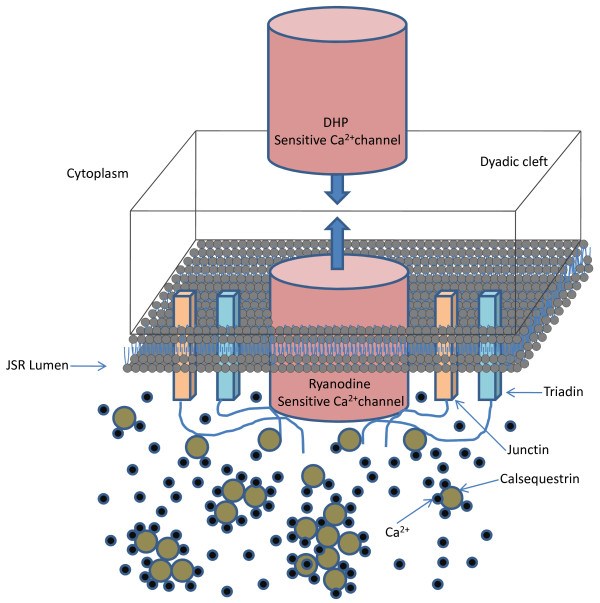
**Schematic of the CICR subsystem**. Figure 5: Schematic of the CICR subsystem. The dyadic coupling unit comprises of a DHP-sensitive *Ca*^2+ ^channel opposing a Ry-sensitive *Ca*^2+ ^channel. The transmembrane proteins triadin and junction along with calsequestrin mediate the interaction between the luminal *Ca*^2+ ^and the RyR thus regulating the release of *Ca*^2+ ^flux from the jSR into the dyadic space.

The SR is an intracellular organelle that consists of two lumped fluid compartments (the jSR and LSR) that communicate (Figure 1A; Figure 3). Like the sarcolemma, the bounding membranes of the jSR and LSR are differentiated regarding their ionic current content and degree of coupling with the sarcolemma. With regard to ionic currents, the LSR membrane has a thapsigargin-sensitive SERCA pump for pumping *Ca*^2+ ^into the LSR lumen against a concentration gradient. In contrast, the jSR membrane contains an outwardly directed ryanodine (Ry)-sensitive channel for *Ca*^2+ ^release from the jSR to the dyadic space. The jSR fluid compartment contains the *Ca*^2+ ^binding protein calsequestin as well as the proteins triadin and junctin, which interact with the ryanodine receptor (RyR) and calsequestrin. This co-located configuration of the RyR receptor, along with the proteins calsequestrin, triadin and junctin which exist on the luminal side of the jSR membrane, constitutes a jSR *Ca*^2+ ^release regulating mechanism called the luminal sensor (Figure 4B; Figure 5). The protein-protein interaction between them plays an important role in regulating the open-state of the RyR *Ca*^2+ ^release channel [25]. A six-state Markovian scheme (Appendix A1, Equations 87-92) is used to describe the dynamics of this interaction and it is called the SR luminal *Ca*^2+ ^sensor. Figure 4B shows a functional diagram of the luminal sensor and its output state is shown connected to the four-state RyR model. Specifically, the sensor adjusts *Ca*^2+ ^dependent rate functions within the ryanodine receptor model, which affects the open probability *P_o _*of the SR *Ca*^2+ ^release channel.

**Figure 6:**
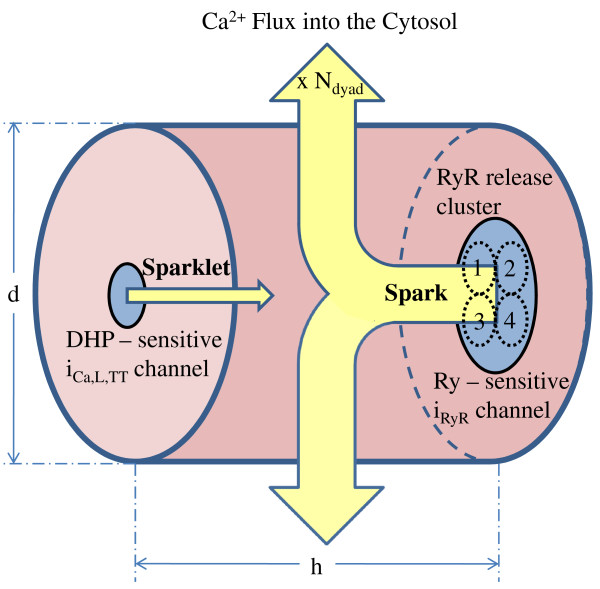
**Schematic of the Dyadic nanodomain**. Figure 6: Schematic of the LCC-RyR coupling. The dyadic cleft allows *Ca*^2+ ^signaling between a DHP-sensitive *Ca*^2+ ^channel opposing a cluster of Ry-sensitive *Ca*^2+ ^channels (represented as the release channel *i_RyR _*in our model). The trigger sparklet due to *Ca*^2+ ^entering via DHP-sensitive *i*_*Ca*,*L*,*TT *_channel causes a spark as a result of release from a cluster of opposing RyR channels. The large amount of *Ca*^2+ ^released subsequently diffuses into the cytosol.

With regard to coupling, the DHP and Ry-sensitive *Ca*^2+ ^channels are assumed to be located on opposite sides of the small dyadic fluid space (nanodomain) as shown in Figure 6, and coupled functionally by a CICR mechanism. The dyadic space is assumed to be in fluid communication with the cell cytoplasm via a restricted diffusion region. In contrast, the LSR is not functionally coupled to the sarcolemma, but rather is in contact with the cytoplasm via the SERCA pump (as shown in Figure 3). Table 4 provides values for parameters used to model the intracellular ion transport.

**Table 4:** Parameters used to model intracellular ion transport

Parameter	Definition	Value	References
$k_{12}^{PLB}$	rate of *PLB_dp _*phosphorylation	6800 *s*^-1^	[77]*

$k_{21}^{PLB}$	rate of *PLB_p _*dephosphorylation	1000 *s*^-1^	[77]*

K_*cyt,serca*_	Maximal binding/release rate of *Ca*^2+ ^from cytosol to SERCA	6250 *s*^-1^	[77]*

*K_serca,sr_*	Maximal binding/release rate of *Ca*^2+ ^from SERCA to LSR	6.25 *s*^-1^	[77]*

*SERCA_tot_*	Total amount of SERCA	47.0 *μM*	[77]*

*PSR*	Phospholamban to SERCA ratio	1.0	[77]*

*PKA*_*ac*t_	Relative regulatory activity of PKA	0.1	[77]^†^

*τ_tr_*	Time const. for transfer of *Ca*^2+ ^from LSR to jSR	7.0 × 10^-3 ^*s*	[30]^‡^

*τ_Na_*	Time const. for transfer of *Na*^+ ^from dyad to myoplasm	1.0 × 10^-3 ^*s*	[30]^‡^

*τ_Cs_*	Time const. for transfer of *Cs*^+ ^from dyad to myoplasm	6.0 × 10^-3 ^*s*	[30]^‡^

*P_ryr_*	Permeability of the RyR channel	1.714 × 10^-7 ^*μL *· *s*^-1^	[27,28,13]^‡^

*D_Ca_*	Diffusion constant for *Ca*^2+ ^in the dyadic space	100.0 *μm*^2^*s*^-1^	[119]*

*N_h_*	Density of high-affinity *Ca*^2+ ^binding sites	200.0 *μM*	[119]^‡^

*N_l_*	Density of low-affinity *Ca*^2+ ^binding sites	16.0 *μM*	[119]^‡^

*K_l_*	Half-saturation value of low-affinity *Ca*^2+ ^binding site	1100.0 *μM*	[119]*

*K_h_*	Half-saturation value of high-affinity *Ca*^2+ ^binding site	13.0 *μM*	[119]*

[*Mg*^2+^]*_myo_*	Intracellular *Mg*^2+ ^concentration	634 *μM*	[124]*

[*fluo*3]*_tot_*	total concentration of indicator dye	100.0 *μM*	Measured

$k_{fluo3}^{+}$	association rate of *Ca*^2+ ^binding to dye fluo-3	80 *μM *^-1 ^· *s*^-1^	[125]*

$k_{fluo3}^{-}$	dissociation rate of *Ca*^2+ ^binding to dye fluo-3	90 *s*^-1^	[125]*

Table 4 Parameters used to model the intracellular ion transport I*_cyt,serca_*, I*_serca.sr_*, I*_tr_*, I*_RyR_*, and *Ca*^2+ ^diffusion/buffering. Adopted (*), derived (†) or estimated (‡) from the cited sources.

### The Dyadic Coupling Unit (DCU)

In describing this functional unit it is necessary to provide progressively more detailed descriptions of the component elements of the individual dyad, particularly the geometrically opposed DHP and Ry-sensitive *Ca*^2+ ^channels, as well as the geometry and buffering properties of the small dyadic space. As will be shown, we have described the dynamics of both types of *Ca*^2+ ^channels using Markovian state models (Figure 4) which include features such as *Ca*^2+ ^mediated channel inactivation, a graded CICR process with a "calcium gain" of approximately 6-7, and two-dimensional *Ca*^2+ ^diffusion within the dyadic space. Crank [26] discusses diffusion problems in a two-phase heterogeneous medium and shows that diffusion through a system of barriers (RyR feet structures in the dyadic cleft space) can be approximated by diffusion in the same region without barriers but with a reduced effective diffusion coefficient. We hence take this approach in modeling the *Ca*^2+ ^diffusion by solving the 2-D Laplacian equation Appendix A3 (Equations 147-150) in the DCU without explicitly accounting for local potential fields. The DHP-sensitive *i_Ca,L,TT _*channel brings in trigger *Ca*^2+ ^(0.1 pA which is of the same order as measured by Wang et. al. [13]) causing a sparklet (a local increase in *Ca*^2+ ^concentration at the mouth of the channel). This trigger *Ca*^2+ ^causes a release from a cluster of opposing RyR channels, causing a spark. This combined release from a cluster of RyR channels causing a spark is represented as the release from a unitary RyR channel (*i_RyR_*) in our model (shown in Figure 6). The characteristics of elemental *Ca*^2+ ^release from a unitary RyR channel in our model agrees with data in terms of amplitude which is of the order of 3 pA (reported by Cheng et al. [27] and Blatter et al. [28]) and duration (full duration at half maximum (FDHM)) which is of the order of 50 ms (reported by Zima et al. [14]).

The single DCU in our model represents the lumped activity of a large number of individual dyads (e.g. 10,000), and it is charged with the task of forming the cytosolic *Ca*^2+ ^transient (hence mechanical contraction) each beat of the cardiac muscle cell. In response to tonic application of voltage clamp pulses, the DCU strongly depends on an adequate supply of *Ca*^2+ ^from the SR. The measurements of Diaz et al. [29] show that, although trigger current may be supplied regularly by tonic voltage clamp pulses, there is an inherent steady-state dependence of the magnitude of *Ca*^2+ ^release on the particular value of SR *Ca*^2+ ^content (i.e., there is a relationship between SR *Ca*^2+ ^content and peak [*Ca*^2+^]*_myo _*; Figure 4 Diaz et al. [29]). This plot gives us a glimpse of the input-output relationship of the dyadic coupling unit and indicates that SR *Ca*^2+ ^content is an important controlling variable for the CICR process implemented by the DCU model.

### L-Type Ca^2+ ^Current

A multiple state characterization of *I_Ca,L _*in rat ventricular myocytes has been reported previously by our group [30]. The gating scheme used in this *I_Ca,L _*model has an additional high voltage state *C*6*_dhpr _*as shown in Figure 4A which is introduced to reproduce *I_Ca,L _*tail currents. Upon voltage-dependent activation, the channel achieves the primary open state *O*2*_dhpr_*. The degree of opening is enhanced in the presence of activated calmodulin-dependent kinase (*CaMKII_act_*) [31-33] which is known as *Ca*^2+^-dependent facilitation (CDF). CaMKII has been shown to tether to the *I_Ca,L _*channel [34] functioning as a *Ca*^2+ ^signalling sensor for facilitation. The open probability of the *I_Ca,L _*channel is also increased in the presence of activated calcineurin (*CaN_act_*) [35]. These two effects are modeled as shown in Figure 4A, where $k_{12}^{dhpr}$ is a function of both *CaMKII_act _*and *CaN_act _*besides voltage. The gating scheme features two different pathways for inactivation of the open state. The pathway *O*2*_dhpr _*↔ *C*5*_dhpr _*accounts for voltage-dependent inactivation, whereas the pathway *O*2*_dhpr _*↔ *C*4*_dhpr _*↔ *C*5*_dhpr _*with *Ca*^2+^-calmodulin (*Ca_4_CaM*) dependent rate constant $k_{24}^{dhpr}$ accounts for the fast and slow phases of *Ca*^2+^-dependent inactivation (CDI) [36]. Transitions to the closed state via both the inactivation pathways are suppressed in the presence of *CaMKII_act _*and *CaN_act _*to allow CDF. A 2-state Markovian model allows *Ca*^2+ ^mediated interaction between calmodulin and the *I_Ca,L _*channel. As shown in Figure 4A, state *S*2*_dhpr_*, which denotes *Ca_4_CaM *bound to the IQ-motif of the *I_Ca,L _*channel, modulates calmodulin dependent *Ca*^2+^-induced inactivation. This is in agreement with the findings of Nikolai M. Soldatov [37]. Our approach to modeling the effects of the proteins *CaM*, *CaMKII*, and *CaN *on the *I_Ca,L _*channel was based on the premise that they are co-localized with the channel itself [35]. Most of the beat-to-beat modulation is produced by *CaM *and *CaMKII*, whereas *CaN *is constitutively active in the dyad [38]. Tables 5, 6 and 7 provide values for the rate constants used to model *Ca*^2+^/CaM binding, CaM buffering, *Ca*^2+^/CaM/CaMKII as well as *Ca*^2+^/CaM/CaN interactions.

**Table 5:** Rate constants used to model Ca/CaM binding and CaM buffering

Rate Constant	Value	Rate Constant	Value
$k_{02}^{CM}$	4.8387^‡^	$k_{42B}^{CM}$	$k_{42\;}^{CM}$

$k_{20}^{CM}$	10.0*	$k_{0Bon}^{CM}$	3.5 × 10^-4‡^

$k_{24}^{CM}$	3.4722^‡^	$\;k_{0Boff\;}^{CM}$	1.4 × 10^-6‡^

$k_{42\;}^{CM}$	500.0*	$k_{2Bon\;}^{CM}$	$k_{0Bon}^{CM}$

$k_{02B}^{CM}$	$k_{02}^{CM}$	$k_{2Boff\;}^{CM}$	$\;k_{0Boff\;}^{CM}$

$k_{20\;B}^{CM}$	$k_{20}^{CM}$/100	$\;k_{4Bon\;\;}^{CM}$	$k_{0Bon}^{CM}$

$k_{24\;B}^{CM}$	$k_{24}^{CM}$	$k_{4Boff\;}^{CM}$	$\;k_{0Boff\;}^{CM}$

Table 5: Rate constants used to model Ca/CaM binding and CaM buffering. Adopted (*) or estimated (‡) from Saucerman et. al. [38].

**Table 6:** Rate constants used to model Ca/CaM/CaMKII interactions

Rate Constant	Value	Rate Constant	Value
*k*_*PP*1_	1.72	$k_{32}^{CK}$	2.2

*k*_*mPP*1_	11.5	$k_{45}^{CK}$	3.35 × 10^-3^

$k_{21\;}^{CK}$	65.67164	$k_{54}^{CK}$	3.4722

$k_{12\;}^{CK}$	328.3582	$k_{46}^{CK}$	2.2 × 10^-3^

$k_{13\;}^{CK}$	3.4722	$k_{64}^{CK}$	65.67164

$k_{31\;}^{CK}$	3.35	$k_{56}^{CK}$	328.3582

$k_{23\;}^{CK}$	65.67164	$k_{65}^{CK}$	65.67164

Table 6: Rate constants used to model Ca/CaM/CaMKII interactions. All the above parameters are adopted from Saucerman et. al. [38].

**Table 7:** Rate constants used to model Ca/CaM/CaN interactions

Rate Constant	Value	Rate Constant	Value
$k_{Caon}^{CN}$	2.0	$k_{0on}^{CN}$	46.0

$k_{Caoff}^{CN}$	1.0	$k_{0off}^{CN}$	537.966

$k_{02}^{CN}$	4.8387	$k_{2on}^{CN}$	46.0

$k_{20}^{CN}$	0.0606	$k_{2off}^{CN}$	3.2604

$k_{24}^{CN}$	3.4722	$k_{4on}^{CN}$	46.0

$k_{42}^{CN}$	0.199362	$k_{4off}^{CN}$	1.3 × 10^-3^

Table 7: Rate constants used to model Ca/CaM/CaN interactions. All the above parameters are adopted from Saucerman et. al. [38].

Our previous study [30] was focused on the characterization of the *I_Ca,L _*channel under conditions of low [*Ca*^2+^] in the dyadic space and myoplasm. In fact, *Ca*^2+^- release from the jSR was blocked by administering a relatively high dose of ryanodine (20 *μ*M) in all experiments. Therefore, to study the additional influence of SR *Ca*^2+ ^release on *I_Ca,L_*, we modified the original *I_Ca,L _*model to better characterize the process of *Ca*^2+^-dependent inactivation. In the modified *I_Ca,L _*model, majority of the structure for the 6-state dynamic scheme remains the same, however changes have been made to the voltage-dependent inactivation rate function ($k_{25}^{dhpr}$), and the *Ca*_4_*CaM *dependent inactivation rate function ($k_{24}^{dhpr}$). The specific formulas for the Markovian state equations and the modified $k_{25}^{dhpr}$ and $k_{24}^{dhpr}$ functions used in this study are given in Appendix A1 (Equations 4-50). Our adjustments consist of reducing the contribution from voltage-dependent inactivation process and strengthening the *Ca*_4_*CaM*-dependent inactivation process. The rate constants $k_{36}^{dhpr}$ and $k_{63}^{dhpr}$ are constructed in order to provide a re-excitation window during the return of the clamp voltage to the resting potential. With these adjustments, the Markovian state description for *I_Ca,L _*can provide good fits to measured *I_Ca,L _*data under both test conditions (presence and absence of ryanodine -sensitive *Ca*^2+ ^release) as well as produce tail currents during repolarization from large clamp voltages (≥ 40 mv).

### Calcium Buffering in the Dyadic Space

Previous modeling work by Post et al. [39], and Post and Langer [40] considered the effect of *Ca*^2+ ^binding sites on the inner sarcolemmal leaflet. Following these authors, we included low-affinity (*k_d _*= 1.1 mM) and high-affinity (*k_d _*= 1.3 *μ*M) *Ca*^2+ ^binding sites on SL wall boundary of cylindrical dyadic space. The presence of these membrane bound sarcolemmal *Ca*^2+ ^binding sites in our model has significant physiological implications, in that it prevents local dyadic *Ca*^2+ ^concentration near the "mouth" of DHP-sensitive *I_Ca,L _*channel from becoming excessively high. Allowing such a condition to occur can cause a reversal of the *I_Ca,L _*current, which does not occur physically during normal jSR release. Addition of these SL *Ca*^2+ ^binding sites does not significantly slow the build-up of *Ca*^2+ ^within the dyadic space, nor is the *Ca*^2+^-induced *Ca*^2+ ^release mechanism affected [41].

### Ca^2+ ^Release Channel

The gating characteristics of the Ry-sensitive release channel are not only modulated by the dyadic *Ca*^2+ ^concentration at its mouth but also the jSR *Ca*^2+ ^concentration via the luminal sensor. Several RyR gating schemes have been deduced from isolated RyR currents measured in lipid bilayers, including: (1) the 4-state scheme developed by Keizer and Levine [42] with 4 *Ca*^2+ ^binding for activation and 3 *Ca*^2+ ^binding for inactivation to explain the "adaptation" of the RyR observed by Gyorke and Fill [43]; (2) the 6-state scheme suggested by Zahradnikova and Zahradnik [44], which allowed opening the RyR channel upon binding of a single calcium ion; and (3) the Markov model proposed by Keizer and Smith [45], which can be dynamically switched among the six, five and four-state representations during the simulation as *Ca*^2+ ^levels vary. Stern et al. [6] demonstrated that none of these schemes yielded stable local control of SR release, even with extensive adjustment of parameters. Stern et al. [6] further reported that all schemes resulted in models that manifested local instability, as indicated by failure of release to terminate after activation, or global instability caused by spontaneous activation by resting [*Ca*^2+^]*_myo_*. Since many of the kinetic gating schemes derived from lipid bi-layer data fail to support stable E-C coupling in simulations, he concluded that the RyR gating process in situ may differ considerably from that in bi-layers.

Our gating scheme is patterned after the release channel used in the model of Stern, Pizarro and Rios [46] (Figure 4B), where the channel is assumed to have four states: rest (closed), activated (open), inactivated (closed) and refractory (closed). The activation gate is opened by the simultaneous, cooperative binding of two *Ca*^2+ ^ions, whereas inactivation depends on the binding of a single *Ca*^2+ ^ion. Four additional features have been built into our model: (a) the crucial role of the luminal SR *Ca*^2+ ^sensor (Figure 5) in assisting the inactivation of the RyR channel is modeled via the dependence of all the rate constants in the 4-state RyR model, on the degree of interaction between the RyR and the proteins triadin and junctin (Figure 4B); (b) *CaMKII_act _*dependent enhancement in RyR release [47-50] is modeled via the rate functions $k_{12}^{ryr}$, $k_{41}^{ryr}$, $k_{43}^{ryr}$ and $k_{32}^{ryr}$; (c) a stronger *Ca*^2+ ^dependent inactivation of the RyR channel (at a fixed depolarization level), is adopted to reflect recent observations that the inactivation of the RyR depends on the high local *Ca*^2+ ^concentration consequential to their own *Ca*^2+ ^release [51]; and (d) inactivation of the RyR at different depolarization levels is made dependent on local *Ca*^2+ ^concentration (i.e., [*Ca*^2+^]*_ryr _*at the "mouth" of the RyR channel on the dyadic side) as per Wier et al. [52] and Zucchi et al. [53]. Our initial studies indicated that the repriming rate ($k_{32}^{ryr}$) in the RyR gating scheme of Stern et al. [46] was quite large, which can lead to a saturated open probability of the *Ca*^2+ ^release channel. This occurs during the later phase of channel inactivation, where the large value of $k_{32}^{ryr}$ tends to reactivate the channel, resulting in saturated *Ca*^2+ ^release. Therefore, we utilized a value of $k_{32}^{ryr}$ that is 10% of that used by Stern et al. [46], and further assumed that the unitary permeation flux of the jSR release channel is proportional to jSR luminal *Ca*^2+ ^concentration ([*Ca*^2+^]*_jSR_*). The specific equations for the *Ca*^2+ ^release model are given in Appendix A1 (Equations 73-92).

### Luminal RyR sensor

The RyR *Ca*^2+ ^release is modulated by a multi-molecular *Ca*^2+ ^signalling complex which is localized to the junctional SR [54-56]. This complex consists of the ryanodine receptor (RyR) which functions as a *Ca*^2+ ^conducting pore [57,58], calsequestrin (CS) which acts as the *Ca*^2+ ^binding protein [59,60], and the junctional SR transmembrane proteins triadin [61] and junctin [62]. It was known previously that the proteins triadin and junctin anchor calsequestrin to the ryanodine sensitive receptor [61]. More recently, it has been observed that the protein-protein interactions between triadin, calsequestrin and RyR modulate sarcoplasmic reticulum calcium-release in cardiac myocytes [25]. Triadin and junctin are structurally homologous proteins [63], but the functional differences in their roles are unclear at present. Therefore we have refrained from modeling these proteins separately with unique roles, and will hereafter only mention triadin. The RyR model proposed by Shannon et al. [64] takes a heuristic approach towards free SR *Ca*^2+ ^concentration dependent luminal control of the RyR channel. Our detailed biophysical model of the luminal sensor is strongly based on the recent findings of Terentyev et al. [25] which uncovers complex [*Ca*^2+^]*_jSR_*) dependent, CS mediated mechanistic interaction of the protein triadin with the RyR channel.

The triadin protein facilitates SR *Ca*^2+ ^release by sensitizing the RyR to activation by the trigger current *I_Ca,L,TT _*. This is incorporated in our model by allowing the rate constants in the 4-state Markovian model for the RyR (Figure 4B-ii) channel to be functions of the activated state *A*1*_ls_*, which represents the degree of binding between triadin and RyR, in the 6-state model for the luminal sensor (Figure 4B-i).The degree of triadin assisted anchoring of CS to the RyR channel [61,62] is denoted by the state *I*2*_ls_*. Triadin is also known to exist in a form bound to CS alone [25] denoted by the state *I*3*_ls _*and in its free unbound form represented as *I*4*_ls _*in the model. CS which is known to exist in a *Ca*^2+ ^bound form modeled by *B*6*_ls _*also modulates SR *Ca*^2+ ^release by influencing the open probability of the RyR channel [65-67], via interactions with Triadin [54,66,25]. The degree of unbound CS is denoted by *B*5*_ls _*in the model.

During the diastolic period the relatively large concentration of available free *Ca*^2+ ^in the SR results in most of the CS being bound to *Ca*^2+^, decreasing the degree of interaction between CS and triadin. This enables a strong interaction between the available unbound triadin and the RyR channel increasing its propensity for trigger *Ca*^2+ ^induced SR release. Our model incorporates this property by facilitating movement of states towards *A*1*_ls _*and *B*6*_ls _*in the presence of large SR *Ca*^2+ ^concentration. Following SR *Ca*^2+ ^release, a reduced luminal *Ca*^2+ ^concentration in the jSR causes an increase in the amount of free calsequestrin (*B*5*_ls _*in the model) available to bind with triadin. This results in a decrease in the extent of interaction between triadin and RyR (*A*1*_ls _*in the model), thus inhibiting the RyR channel and leading to robust termination of SR *Ca*^2+ ^release in cardiac myocytes [66]. This release termination mechanism is incorporated in our combined RyR-Luminal sensor model (Figure 4B and Appendix A1, Equations 73-92).

Due to the lack of in-vivo measurements on individual state transitions, the rate constants in our novel luminal sensor model (Table 8) are chosen such that (i) the triadin mediated sensitization of the RyR channel (via *A*1*_ls_*) provides adequate peak RyR release which translates into the upstroke velocity of the cytosolic *Ca*^2+ ^transient; (ii) the luminal sensor mediated RyR channel inactivation (via *A*1*_ls_*) causes timely release termination resulting in a cytosolic *Ca*^2+ ^transient duration, physiological for a rat ventricular myocyte; (iii) the rate of post-release RyR recovery results in appropriate channel refractory characteristics. In the case of channels where there is an interaction between ion flow (which is not at equilibrium) and its gating mechanism the microscopic reversibility criteria does not hold true [68,69]. The RyR channel which is solely modulated by [*Ca*^2+^] (both on the dyadic side ([*Ca*^2+^]*_ryr_*) as well as the luminal side ([*Ca*^2+^]*_jSR_*)) experiences a strong interaction of *Ca*^2+ ^flow through itself and its gating mechanism described as [*Ca*^2+^]*_ryr _*induced self-inhibition and the luminal sensor induced inactivation. Hence, the rate constants of the luminal sensor model (Figure 4B-i) are not constrained by microscopic reversibility criteria. However, a stability constraint in the form of, the sum of probabilities of all possible states corresponding to triadin (*A*1*_ls_*, *I*2*_ls_*, *I*3*_ls_*, *I*4*_ls_*) and CS (*I*2*_ls_*, *I*3*_ls_*, *B*5*_ls_*, *B*6*_ls_*) being equal to one is explicitly imposed Appendix A1, Equations 87-88) on the model of the luminal sensor in order to avoid run-off.

**Table 8:** Parameters used in the luminal sensor model

Rate Constant	Value	Rate Constant	Value
$k_{12}^{ls}$	88.16	$k_{23}^{ls}$	57.9

$k_{21}^{ls}$	4.1	$k_{32}^{ls}$	2.42

$k_{14}^{ls}$	0.5	$k_{35}^{ls}$	150.3

$k_{41}^{ls}$	85.7	$k_{53}^{ls}$	25.5

$k_{42}^{ls}$	2.98	$k_{52}^{ls}$	88.16

$k_{43}^{ls}$	25.5	$k_{56}^{ls}$	1.2

$k_{34}^{ls}$	150.3	$k_{65}^{ls}$	401.7

Table 8: Static rate constants used in the model for the luminal sensor

### Ca^2+ ^Buffering in Myoplasm and SR

*Ca*^2+ ^buffers play an important role in sequestering a fraction of the total *Ca*^2+ ^released during E-C coupling and contraction. These buffers include: (a) calmodulin (CaM), which is assumed to be uniformly distributed in the myoplasm and dyadic space; (b) troponin in the bulk myoplasm; and (c) calsequestrin (CS) in the jSR. The dyadic space has been shown to be accessible to calmodulin (CaM), but mostly inaccessible to fluorescent dyes [70,71]. Therefore, we consider the dyadic space filled with calmodulin, but not fluo-3. This provides a more direct calcium communication pathway between the DHP and Ry receptors. The rate constants for *Ca*^2+ ^binding to calmodulin were based on a model from [22], whereas those for *Ca*^2+ ^binding to troponin were taken from Potter and Zott [72]. Rate constants used to describe *Ca*^2+ ^binding to calsequestrin were based on the study of Cannell and Allen [73], whereas those for *Ca*^2+ ^binding to troponin-Mg complex were adopted from Lindblad et al. [74].

It is well recognized that fluorescent indicator dyes introduced into the cytosol also act as *Ca*^2+ ^buffers [22], even at submillimolar concentrations [51]. In our simulations, we have used 100*μ*M fluo-3. We assume that the quantity observed experimentally as a "calcium concentration" signal is actually the calcium complexed with fluo-3, or [CaF3]. The differential equation describing the change in [CaF3] with time is given in Appendix A2 (Eq. 138) which follows from Shannon et al. [75].

### Ca^2+^-Uptake

Cytosolic *Ca*^2+ ^is pumped into the LSR (Figure 1A) by a *Ca*^2+^-ATPase, which lowers [*Ca*^2+^]*_myo _*and helps to induce relaxation in cardiac muscle. The transport reaction involves two *Ca*^2+ ^ions and one ATP molecule [76], and it is represented by the description given for *I_cyt,serca _*and *I_serca,sr _*in Appendix A1 (Equations 51-63). The model used for the uptake pump is adopted from Koivumaki et al. [77] and takes into account both the forward flux of *Ca*^2+ ^from the cytosol to the LSR lumen and the backward flux from the LSR to the cytosol along with the *Ca*^2+ ^buffering action of the SERCA protein. The phospholamban (PLB) to SERCA ratio has been fixed to 1.0, assuming almost equal availability of both the proteins. *CaMKII_act _*affects the SERCA pump via direct phosphorylation assisting in enhancement of SR *Ca*^2+ ^transport by increasing the pumping rate [78] and indirectly via phosphorylation of PLB [79] relieving the inhibition caused by PLB on the SERCA pump in turn increasing the sensitivity of the pump for *Ca*^2+ ^uptake. These two effects are modeled by allowing the rate constants for *Ca*^2+^+ binding to/release from the SERCA pump as well as the rate constant for phosphorylation of PLB to be a function of *CaMKII_act _*in the cytosol. The activating role of CaN in modulating the SERCA pump [80,81] is accounted for in our model by allowing the rate constant for phosphorylation of PLB to be dependent on available *CaN_act _*in the cytosol. The relative regulatory role of the enzyme protein kinase A(PKA) is fixed at 0.1, as its modulatory effect is beyond the scope of this study. The current *I_cyt,serca _*dictates the transport of *Ca*^2+ ^between the cytosol and the SERCA protein. Similarly, the current *I_serca,sr _*dictates the transport of *Ca*^2+ ^between the SERCA protein and the LSR. The difference in these *Ca*^2+ ^currents accounts for the *Ca*^2+ ^buffered by the SERCA protein. The jSR is subsequently refilled by *Ca*^2+ ^diffusion from the LSR. The differential equations describing the *Ca*^2+ ^balance and particularly the transfer between two separate SR compartments (jSR and LSR) are provided in Appendix A1 (Eq. 72).

### Ca^2+^-Extrusion via Sarcolemmal Ca^2+ ^Pump

Although the sarcolemmal *Ca*^2+^-pump has a high affinity for [*Ca*^2+^]*_myo_*, its transport rate is far too slow for it to be an important factor in *Ca*^2+ ^fluxes during the cardiac cycle. It might, however be more important in long-term extrusion of *Ca*^2+ ^by the cell. Our model of the plasma membrane *Ca*^2+ ^ATPase pump current is adopted from Sun et al. [30]. We have used a constant value of half activation constant *k_mpca _*= 0.5*μ*M in our model on the basis of measurements by Caroni et al [82].

### Ca^2+^-Extrusion via Na^+^/Ca^2+ ^Exchanger

In mammalian cardiac cells, it is generally accepted that the *Na*^+^/*Ca*^2+ ^exchanger has a stoichiometry of 3*Na*^+^:1*Ca*^2+ ^[83]. *I_NaCa _*(again, the combination of *I_NaCa,TT _*and *I_NaCa,SL_*) is important in removing *Ca*^2+ ^during twitch relaxation, in competition with *I_up_*. A simple thermodynamic *Na*^+^/*Ca*^2+ ^exchanger current model [84] may be sufficient to predict the direction of *Ca*^2+ ^transport by *Na*^+^/*Ca*^2+ ^exchange and the driving force, however the amplitude is subject to kinetic limitations (depending on substrate concentrations). A more comprehensive *Na*^+^/*Ca*^2+ ^exchanger current equation [85] is adopted in our model, which includes factors for allosteric *Ca*^2+ ^activation and the transport for *Na*^+ ^and *Ca*^2+ ^inside and out. The maximal flux through the exchanger *V_max _*is estimated to ensure that the *Ca*^2+ ^ion transport (which is voltage dependent) via the *Na*^+^/*Ca*^2+ ^exchanger matches the influx of *Ca*^2+ ^via the *I_Ca,L _*channel [86,87], maintaining whole cell *Ca*^2+ ^homeostasis.

### Na^+^/Cs^+ ^Pump

The *Na*^+^/*K*^+ ^pump, helps in maintaining homeostasis of the intracellular *Na*^+ ^ion concentration. ATPase activity powers the pump, as it generates an outward *Na*^+ ^flux and an inward *K*^+ ^flux with a *Na*^+ ^to *K*^+ ^stoichiometry of 3 to 2 [88]. However, in our experimental protocol, external solution in the bath was normal Tyrode (1 mM *Ca*^2+^) with *Cs*^+ ^substituted for *K*^+ ^in order to block the inward rectifier *K*^+ ^current. The internal solution in the pipette contained Cesium aspartate supplemented with 20 mM *CsCl*, 3 mM *Na*_2_*ATP *, 3.5 mM *MgCl*_2 _and 5 mM HEPES.

Activation of the electrogenic sodium pump in mammalian non-myelinated nerve fibres [89], skeletal muscle [90] and rat brain cells [91] by *Cs*^+ ^is reported in the literature. It is observed that the late effects of reducing extracellular *K*^+ ^concentration ([*K*^+^]*_o_*) to 0 mM in mammalian cardiac muscle can be prevented by including appropriate concentrations of other activator cations of the *Na*^+^/*K*^+ ^pump such as *Cs*^+ ^in the 0 mM [*K*^+^]*_o _*bathing solution [92]. Monovalent cations (including *Cs*^+^) were also added to *K*^+ ^free bathing solution to reactivate the sodium pump in guinea-pig ventricular myocytes [93]. The effectiveness of *Cs*^+ ^as an external cation in activating the electrogenic sodium pump is known to be lesser than potassium [92].

We have represented *I_NaCs _*in our model by the expression for *Na*^+^/*K*^+ ^pump formulated by Linblad et al. [74] replacing *K*^+ ^ion concentrations with the *Cs*^+ ^ion concentration. While ensuring whole cell *Na*^+ ^ion balance, the peak *Na*^+^/*Cs*^+ ^pump current is modified to be one-sixth to account for the decreased potency of the cation *Cs*^+ ^in activating the pump. The voltage-dependence of *I_NaCs _*is adopted from the data on *Na*^+^/*K*^+ ^pump from Hansen et al. [94].

## Results

The DCU is a fundamental element in the mechanism of CICR. The sequence of events resulting in CICR is triggered by the *Ca*^2+ ^entering through the *I_Ca,L _*channel. The characteristics of this channel are hence examined in detail to understand its voltage and *Ca*^2+ ^dependent behavior. The ability of the *I_Ca,L _*channel to facilitate graded RyR release is noted. The high gain associated with RyR release is quantified. This is followed by a study of the properties of the RyR channel with a particular emphasis on its inactivation mechanism. The *Ca*^2+ ^mediated interaction between these channels is investigated in detail. This is followed by an effort to understand the role of [*Ca*^2+^]*_jSR _*in CICR. In particular, the relationship of the peak cytosolic calcium transient and the SR *Ca*^2+ ^content is examined. The effect of modulatory agents like caffeine and thapsigargin on SR release/uptake is studied to understand the significance of these mechanisms in facilitating a normal CICR.

### L-type Ca^2+ ^current (I_Ca,L_)

**Figure 7:**
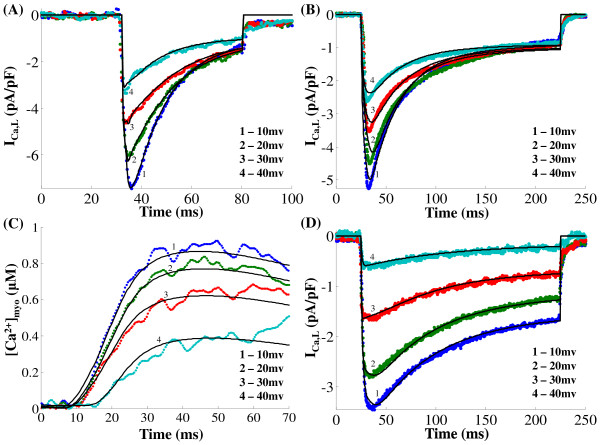
**I_Ca,L _& [Ca^2+^]_myo _- comparison of model generated and experimental data in the positive voltage range**. Figure 7: Model generated and experimentally obtained data from a rat ventricular myocyte clamped to voltages between 10 and 40 mv in steps of 10 mv. The holding potential used is -40 mv. Data is obtained from Dr. Philip. T. Palade's lab. (A) *I*_*Ca*,*L *_in the presence of *Ca*^2+ ^release from the Ry-sensitive channel. The pulse duration used is 50 ms. (B) *I*_*Ca*,*L *_with Ryanodine administered to block RyR release. The pulse duration used is 200 ms. (C) Global cytosolic calcium transients [*Ca*^2+^]*_myo _*in the presence of RyR release. The pulse duration used is 50 ms (D) *I*_*Ca*,*L *_using Barium as the charge carrier in the absence of RyR. The pulse duration used is 200 ms. Solid traces are the model-generated results and dotted lines represent the data

The L-type DHP-sensitive *Ca*^2+ ^channel has a key role in initiating CICR. Hence a thorough analysis of its activation and inactivation mechanisms is considered here. Though the activation of the *I_Ca,L _*channel is solely voltage dependent once activated, the inactivation of the channel is influenced not only by the trans-membrane voltage but also the *Ca*^2+ ^concentration in the vicinity of the channel [95]. The relative con-tribution of the voltage-dependent and *Ca*^2+^-dependent inactivation pathways in the *I_Ca,L _*channel model can be studied by selectively blocking each pathway in the model. Figure 7 shows the model-generated waveform for *I_Ca,L _*under three conditions; (i) control case with both voltage and calcium dependent in-activation pathways intact; (ii) ryanodine applied to allow only the voltage-dependent inactivation and the *Ca*^2+^-dependent inactivation via *I_Ca,L _*self-inhibition in the absence of RyR release; and (iii) *Ca*^2+ ^substituted with *Ba*^2+ ^to facilitate only the voltage-dependent inactivation pathway and block all *Ca*^2+ ^dependent inactivation pathways (*k*24*_dhpr _*set to zero). The protocol used in case (i) was a 50 ms pulse with clamp voltages ranging from 10 mv to 40 mv in steps of 10 mv from a holding potential of -40 mV. In case (ii) and (iii) the pulse duration was increased to 200 ms to replicate the data.

Measured data obtained using the same voltage clamp pulse under all three conditions (Control, Ryan-odine application and Barium substitution) is shown for comparison (data obtained from Dr. Palade's lab was pre-processed to eliminate transients produced by changing clamp voltage in order to obtain model fits using a non-linear least-squares method [18]). The degree of *I_Ca,L _*channel opening is known to be enhanced in the presence of *CaMKII_act _*[33] which is known as *Ca*^2+^-dependent facilitation (CDF). According to Bers et al. [33] the positive regulation of the channel by *CaMKII_act _*requires *Ca*^2+ ^influx (it is not seen when *Ba*^2+ ^is the charge carrier as in Figure 7D and is more strongly apparent when local *Ca*^2+ ^influx is amplified by SR *Ca*^2+ ^release as in Figure 7A). Increase in *CaMKII_act _*seems to play an important function in enhancing *I_Ca,L _*peak amplitude [96], suggesting a critical role for *Ca*^2+^-dependent facilitation. Our model generated results also indicate that a stronger CDF (in Figure 7A) in the presence of RyR *Ca*^2+ ^release causes an enhancement in *I_Ca,L _*peak amplitude (compare the model fits to data in Figure 7A (presence of RyR release) with Figure 7B (no RyR release) and Figure 7C (*Ca*^2+ ^substituted by *Ba*^2+^)). It is important to note that the peak of the *I_Ca,L _*current is reached after the RyR open probability reaches its maximum value owing to the faster dynamics of the RyR channel. Our simulations indicate that following the inward current peak, *Ca*^2+^-dependent inactivation (CDI) is much faster and dominates the response for the duration of voltage clamp (30 ms <t <80 ms). In addition, a comparison of Figs. 7A and 7B shows that, *Ca*^2+^-dependent inactivation (CDI) caused by *Ca*^2+ ^release from Ry-sensitive channels is much more significant than the self-inhibition produced by *Ca*^2+ ^influx via *I_Ca,L _*itself. The graded behavior of the cytosolic *Ca*^2+ ^transient is shown in Figure 7C. Voltage dependent inactivation (VDI) is relatively slow compared with CDI and is best seen in Figure 7D where all *Ca*^2+ ^inactivation effects have been blocked by *Ba*^2+ ^substitution for *Ca*^2+^. Under RyR blockade (Figure 7B), the relatively slow VDI has its major effect during the late phase of the long voltage clamp pulse (e.g., beyond 100 ms) and in a time range where CDI is relatively constant. Quantitative analysis of movement of states (during the depolarizing pulse duration of 50ms) via different pathways in the six state Markovian model shows that 68.24% of the total inactivation of *I_Ca,L,TT _*is via the calcium dependent *O*2*_dhpr_*-*C*4*_dhpr _*pathway, owing to the large *Ca*^2+ ^concentration which the channel is exposed to in the dyad. In contrast, only 19.21% of the total inactivation of *I_Ca,L,SL _*is via the calcium dependent *O*2*_dhpr_*-*C*4*_dhpr _*pathway, because of the low cytoplasmic *Ca*^2+ ^concentration in the vicinity of the channel. Considering the total *I_Ca,L _*current (*I_Ca,L,TT _*+ *I_Ca,L,SL_*), around 63.34% of the *I_Ca,L _*channel inactivation is via the *Ca*^2+ ^dependent pathway.

**Figure 8:**
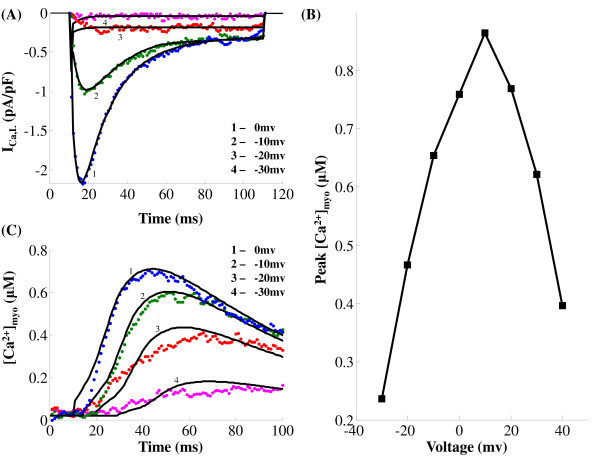
**I_Ca,L _& [Ca^2+^]_myo _- comparison of model generated and experimental data in the negative voltage range**. Figure 8: Model generated and experimentally obtained data from a rat ventricular myocyte clamped to voltages between -30 and 0 mv in steps of 10 mv. The holding potential used is -40 mv. Data is obtained from Dr. Philip. T. Palade's lab. (A) *I*_*Ca*,*L *_invoked by negative clamp voltages in the range of -30 mv to 0 mv in steps of 10 mv. The pulse duration used is 100 ms. (B) Peak *I*_*Ca*,*L *_dependance on clamp voltage. (C) Global cytosolic calcium transients [*Ca*^2+^]*_myo _*at negative (-30 mv ≤ v ≤ 0 mv) clamp voltages. The pulse duration used is 100 ms (D) Peak cytosolic calcium transients ([*Ca*^2+^]*_myo _*) dependence on clamp voltages. Solid traces are the model-generated results and dotted lines represent the data

Figure 8A shows a comparison of model generated and experimentally obtained *I_Ca,L _*data (obtained from Dr. Palade's lab and pre-processed to eliminate transients produced by changing clamp voltage in order to obtain model fits using a non-linear least-squares method [18]) from a rat ventricular myocyte (different from the cell used to obtain data in Figure 7) at negative (-30 mv ≤ v ≤ 0 mv) clamp voltages. The corresponding graded behavior of the cytosolic *Ca*^2+ ^transient is shown in Figure 8C. Figure 8B shows the well known [52] bell-shaped dependence of the peak [*Ca*^2+^]*_myo _*on the clamp voltage.

**Figure 9:**
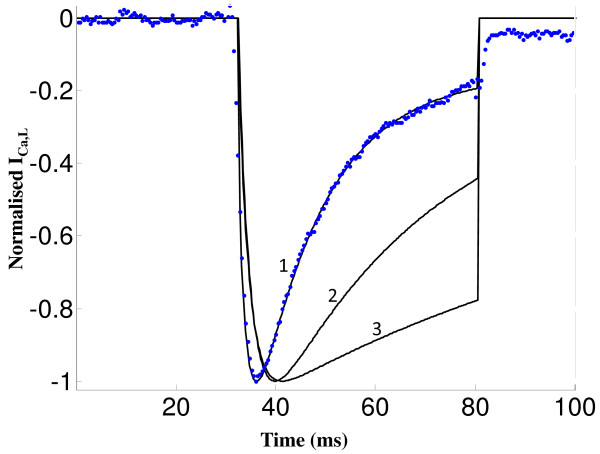
**I_Ca,L _inactivation pathways**. Figure 9: *I*_*Ca*,*L *_inactivation pathways. Voltage clamp protocol used is a 50 ms step pulse to 10 mv from a holding potential of -40 mv. Both model generated results and the data are normalized for easy comparison (1) Control case where both voltage and calcium dependent inactivation pathways are intact. Solid trace is the model-generated result and the dotted line represents the data obtained from Dr. Philip. T. Palade's lab; (2) voltage-dependent inactivation exists however *Ca*^2+^-dependent inactivation is only via *I*_*Ca*,*L *_self-inhibition without RyR release; (3) Only voltage-dependent inactivation pathway preserved by blocking all *Ca*^2+ ^dependent pathways.

Figure 9 shows model generated normalized *I_Ca,L _*obtained by a voltage clamp to 10 mv from a holding potential of -40 mv using the following protocols. Case 1: Normal release is allowed where, following CICR, a strong *Ca*^2+ ^dependent inactivation inhibits the *I_Ca,L _*current as seen in the plot numbered 1 (data obtained from Dr. Palade's lab). Case 2: Ryanodine applied to block RyR release; *Ca*^2+ ^entering via the *I_Ca,L _*channel causes *Ca*^2+ ^induced inactivation, although the magnitude of inhibition is far less than in the control case. Case 3: *Ba*^2+ ^substitution for *Ca*^2+ ^to completely suppress the *Ca*^2+ ^inactivation mechanism; the only inactivation pathway present is the slow voltage dependent pathway [97], which causes substantially reduced recovery compared to cases 1 and 2. Besides the inactivation, it can be seen from Figure 9 that the rate of channel activation, which is evident in the slope of the individual plots during the initial activation phase, is faster in the presence of RyR *Ca*^2+ ^release (a result of enhanced CDF in the presence of elevated *Ca*^2+ ^levels). This highlights the important role of *Ca*^2+ ^in regulating *I_Ca,L _*channel opening and thus controlling the amount of extracellular *Ca*^2+ ^entering the cell.

### I_Ca,L,TT _- dependent Graded SR-release

**Figure 10:**
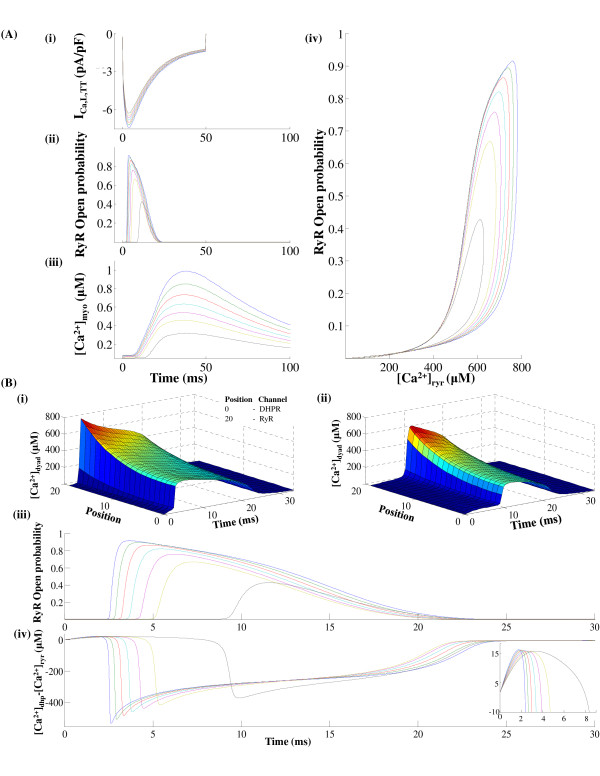
**Graded CICR**. Figure 10: SR calcium release graded by the trigger current *I*_*Ca*,*L*,*TT *_. (A) - (i) Decreasing trigger current *I*_*Ca*,*L,TT *_(ii) With decreasing trigger current, RyR open probability shows a graded decrease in its peak as well as an increasing delay in its onset (iii) Cytosolic calcium transient follows the trend shown by the RyR release channel (a graded decrease in peak and increasing delay in onset) (iv) RyR open probability vs [*Ca*^2+^]*_ryr _*for increasing trigger current *I*_*Ca*,*L*,*TT *_. (B)- (i)Concentration profile in the dyad as a result of RyR release caused by a largest trigger current (see part A(i)) (ii)Concentration profile in the dyad as a result of RyR release caused by a smallest trigger current (see part A(i)) (iii) With decreasing trigger current, RyR open probability shows a graded decrease in its peak as well as an increasing delay in its onset (shown on an expanded time scale) (iv)Difference in concentration between the mouth of the DHP and Ry-sensitive *Ca*^2+ ^channels. (Inset shows the cross-over when this difference becomes negative indicating trigger current dependent latency involved in release).

One of the most important characteristics of the CICR mechanism is the modulation of the RyR *Ca*^2+ ^release based on the amount of trigger calcium delivered via the DHPR channel. The voltage-controlled *I_Ca,L _*channel is the link between the extracellular excitation and the intracellular *Ca*^2+ ^release. As shown in Figure 10A, our model reproduces graded release. It is important to note that the onset of the *Ca*^2+ ^transient is also modulated based on the magnitude of the trigger calcium available to initiate release, as shown in Figure 10A-iii. This is due to the fact that the rate of increase in the open probability of the Ry-sensitive *Ca*^2+ ^release channel is controlled by the amount of trigger *Ca*^2+ ^present at the 'mouth' of this channel, which in-turn is graded by the amount of trigger *Ca*^2+ ^entering the DCU by means of the DHP-sensitive *I_Ca,L,TT _*channel. This mechanism is incorporated in our model by allowing the rate constants in the 4-state Markovian model of the Ry-sensitive *Ca*^2+ ^release channel (Figure 4) to be [*Ca*^2+^]*_ryr _*dependent. Small changes in *i_Ca,L,TT _*cause modulation of pre-release [*Ca*^2+^]*_ryr_*, which dictates the propensity of the RyR channel for a *Ca*^2+ ^release. The pre-release values of the rate constants in the 4-state Markovian model of the RyR channel (nonlinear dependence on [*Ca*^2+^]*_ryr _*as shown in Appendix A1, Equations 79-84) along with the diastolic [*Ca*^2+^]*_jSR _*(which is kept constant in Figure 10) set the peak RyR open probability achieved by the RyR channel as well as the peak [*Ca*^2+^]*_myo_*. The use-dependent adaptation of the RyR channel [13] is reflected in its non-linear response to trigger *Ca*^2+^. As shown in Figures 10B-iii, iv, the delay in the onset of cytosolic *Ca*^2+ ^transient closely follows the modulation of the onset of RyR release. Though occurring in separate compartments, the peak of cytosolic *Ca*^2+ ^transient also tracks the maximum value attained by the open probability of the Ry-sensitive *Ca*^2+ ^release channel. Here, the clamp voltage was held constant at 10 mv to avoid the interference of voltage dependent *Ca*^2+ ^transport via the *Na*^+^/*Ca*^2+ ^exchanger which is co-located [98] with the *I_Ca,L _*channel in the dyad and the scaling down of *I_Ca,L,TT _*corresponds to a fast flicker block of the channels by dihydropyridine.

The slow rising foot that precedes the rapid upstroke (trace at position 0 in Figure 10B-i and B-ii) is the contribution from a single sparklet (Figure 6), which is the result of *Ca*^2+ ^release from a single *i_Ca,L,TT _*channel in our model (0.1 pA), which is of the same order as reported by Wang et. al. [13] bringing trigger *Ca*^2+ ^into a unitary dyad. This L-type *Ca*^2+ ^channel (LCC) triggers release from a cluster of RyR channels causing a *Ca*^2+ ^spark (Figure 6), which results in the rapid increase in [*Ca*^2+^]*_dyad _*that follows the foot (shown in Figure 10B-i and B-ii). As shown in Figure 10B, the latency from the onset of the sparklet foot to the triggered *Ca*^2+ ^spark increases with decrease in the magnitude of *i_Ca,L,TT _*[13]. The SR *Ca*^2+ ^release flux underlying a typical *Ca*^2+ ^spark corresponds to approximately 2 pA [27,28,13]. From RyR single channel conductance measurements in lipid bilayer studies [99], a *Ca*^2+ ^spark translates into a release from around 4 RyR channels (also reported by Blatter et. al. [28]). This single *Ca*^2+ ^spark corresponds to a unitary RyR release (2 pA) in our model. Our model does not attempt to reproduce the stochastic kinetics of the single channel LCC-RyR coupling; however it mimics accurately the average behavior of this stochastic process which is the net *Ca*^2+ ^release flux into the cytosol causing the *Ca*^2+ ^transient. This on/off stochastic nature of the coupling was used earlier to explain the release termination via stochastic attrition. However, it is now known [25] that the luminal sensor plays a fundamental role in an active extinguishing mechanism [51] that effects a robust [*Ca*^2+^]*_jSR _*- dependent closure of the RyR channel. This mechanism for inactivation of the Ry-sensitive *Ca*^2+ ^release channel is accounted for in our model.

**Figure 11:**
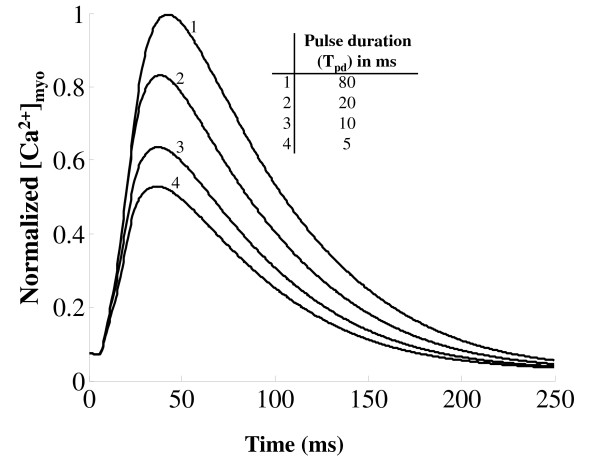
**Effect of depolarization duration on [*Ca*^2+^]*_myo_***. Figure 11: The effect of changing the duration of depolarizing pulses *T_pd _*on the time course of the [*Ca*^2+^]*_myo _*transient. Voltage clamp protocol used is a step pulse to 10 mv from a holding potential of -40 mv. Normalized [*Ca*^2+^]*_myo _*transient corresponding to depolarizing pulses of 5, 10, 20, and 80 ms duration. This model-generated result shows similarity to data in Figure 2, Cannell et al. [100] showing pulse-duration dependent changes in cytosolic *Ca*^2+ ^concentration.

Cytosolic *Ca*^2+ ^transient is also graded by the duration of the *I_Ca,L,TT _*trigger current controlled by the voltage clamp pulse duration. Our model also shows that triggered release can be prematurely stopped by rapid repolarization [100-102]. The effect of depolarization duration on the time course of the cytosolic *Ca*^2+ ^transient is indicated in Figure 11 where the duration of the voltage clamp pulse is decreased from 80 ms to 5 ms (while keeping the clamp voltage constant at 10 mv) resulting in premature stoppage of release and thus a decrease in peak cytosolic *Ca*^2+ ^transient. This effect is a combined result of (i) the modulation of release due to pulse duration dependent change in the amount of trigger *Ca*^2+ ^entering the dyadic coupling unit (DCU) and (ii) the pulse duration dependent change in the relative role of the *Na*^+^/*Ca*^2+ ^exchanger co-located in the DCU.

### High Gain of *Ca*^2+ ^Release

Besides the graded release, an extremely valuable characteristic of the CICR process is the high gain associ-ated with it. A small amount of *Ca*^2+ ^entering the DCU via the *I_Ca,L,TT _*channel causes a large release of *Ca*^2+ ^from the sarcoplasmic stores via the Ry-sensitive *Ca*^2+ ^release channel on the jSR lumen that interacts with the DCU. This high gain is essential in producing physiological cytosolic *Ca*^2+ ^transients when 10000 of these DCU's operate in tandem. Two different definitions of the gain or amplification factor due to CICR have been adopted in our model simulations, namely the ratio of:

1. average integrated RyR flux to average integrated DHPR flux [7]; and

2. the peak *Ca*^2+ ^transient in the presence of CICR to the peak calcium transient in its absence, con-tributed by the trigger calcium alone [3].

Thus, criterion (1) measures calcium gain observed in the dyadic space, whereas criterion (2) measures gain observed in the cytosolic space.

**Figure 12:**
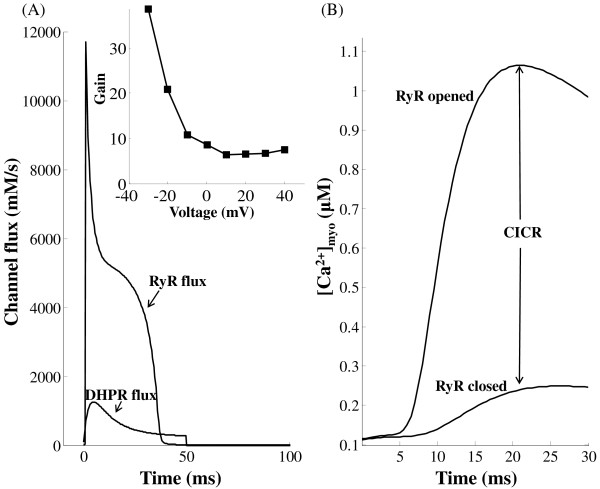
**CICR *Ca*^2+ ^gain**. Figure 12: High gain associated with CICR. Voltage clamp protocol used is a 50 ms step pulse to 10 mv from a holding potential of -40 mv. (A) Gain formulation by comparison of *Ca*^2+ ^flux through the DHP sensitive and RyR sensitive *Ca*^2+ ^channels. The voltage dependence of gain measured using this method is shown in the inset. (B) Gain formulation by comparison of cytosolic calcium transient formed in the presence and absence of RyR *Ca*^2+ ^release.

With regard to the dyadic space, Figure 12A shows the *Ca*^2+ ^flux through DHPR and RyRs, respectively. Figure 12B shows cytosolic calcium transients under conditions of ryanodine blockade and with RyR *Ca*^2+ ^release. Based on our measurements, there is approximately a 2 ms delay from the onset of the DHPR influx to the initiation of the RyR release flux.

At a clamp voltage of 10 mv, criterion (1) applied to our simulation yields an integrated flux ratio of 6.39, whereas a gain of 5.75 is calculated using criterion (2). By either method, a CICR amplification factor of approximately 6 is calculated, which is similar to that reported by Stern [3]. This result is also consistent with gain calculations from the measured data of Fan and Palade [17] on rat ventricular cells. They estimated a gain of approximately 7 using comparisons of the rates of rise of *Ca*^2+ ^transients in the presence and absence of ryanodine. The model generated results are obtained by using a voltage clamp protocol of a 50 ms step pulse to 10 mv from a holding potential of -40 mv.

The inset in Figure 12A shows the voltage dependence of CICR gain formulated using criterion (1). Our model shows a decline in gain as the clamp voltage is increased from -30 mv to 20 mv [52,103]. However, any further increase in clamp voltage results in a small increase in gain (Figure 1C, Altamirano et al. [104]). It is important to note that, with increasing clamp voltage, the decreased ability of the *Na*^+^/*Ca*^2+ ^exchanger (which is co-located [98] in the dyad) to extrude *Ca*^2+ ^partially compensates for the declining trigger current, in facilitating SR release and hence assists in increasing CICR gain.

### RyR Refractory Characteristics

**Figure 13:**
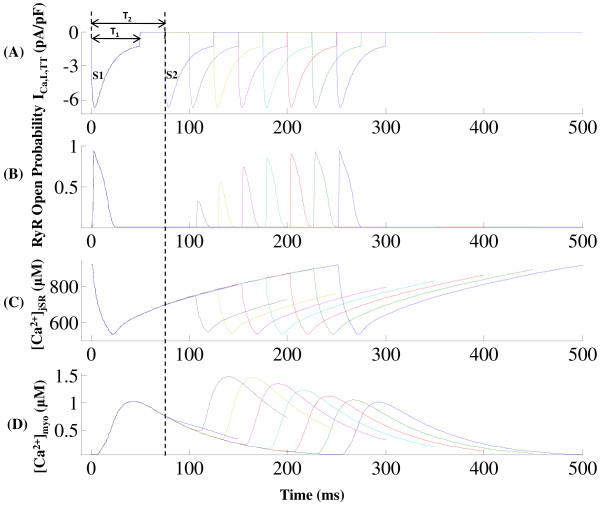
**RyR refractory characteristics**. Figure 13: RyR refractory characteristics. (A) The stimulation protocol comprises of two consecutive stimuli S1 & S2, both of which are identical to the dyadic component (*I*_*C*a,*L*,*TT *_) of trace 1 (*I*_*Ca*,*L *_evoked by a 10 mv clamp pulse) in Figure 7A with a peak amplitude of 6.657 pA/pF and a duration (*T*_1_) of 50 ms. These are applied with their separation in time (*T*_2_) increasing from 50ms to 250 ms in steps of 25 ms. (B) The RyR sensitive *Ca*^2+ ^release channel shows refractory characteristics with an absolute refractory period of 50 ms and a relative refractory period of 250 ms. (C) The corresponding [*Ca*^2+^]*_jSR _*traces reflect partial RyR recovery. (D) Cytosolic calcium transient recovers completely after 250 ms. The dotted line indicates lack of RyR release when *T*_2 _= 75 ms due to insufficient RyR recovery.

Given the fact that the CICR mechanism has a high gain associated with it, it could be prone to unstable behavior if not for the refractory characteristics of the Ry-sensitive release channel [105]. It is now known [25] that the luminal sensor plays a fundamental role in an active extinguishing mechanism [51] effecting a [*Ca*^2+^]*_jSR _*dependent robust closure of the RyR channel which is accounted for in our model. The RyR release fails to become regenerative due to the role of the luminal sensor which after a release occurs, forces the RyR channel into an absolute refractory state followed by a relative refractory state as shown in Figure 13C. When the RyR channel is in the absolute refractory period, [*Ca*^2+^]*_ryr _*drops to a level much below what is caused by a sparklet (trigger *Ca*^2+^) hence it robustly avoids re-excitation. This extremely critical refractory feature of the channel enabled by the RyR luminal sensor acts as a protective mechanism against premature *Ca*^2+ ^release in the wake of a secondary excitation that occurs before jSR can be filled back to the control level. The refractory nature of the channel is caused by the [*Ca*^2+^]*_jSR _*dependent inhibition induced by the protein triadin from the luminal side of the RyR channel. This restraining lock on the RyR channel assists in reloading the jSR.

A dual stimulus protocol (S1-S2) was employed to study RyR refractoriness. The RyR channel was stimulated initially by stimulus trigger current S1, followed after an interval (*T*_2 _in Figure 13A) by an identical stimulus current S2. Each of the two stimuli directed into the dyadic coupling unit is the dyadic component (*I_Ca,L,TT _*) of trace 1 (*I_Ca,L _*evoked by a 10 mv clamp pulse) in Figure 7A with a peak amplitude of 6.657 pA/pF and a duration (*T*_1 _in Figure 13A) of 50 ms. The stimuli S1 & S2 were kept identical to delineate the effects of RyR refractoriness on CICR. The time interval between the two stimuli (*T*_2_) was gradually reduced from 250 ms to 50 ms in steps of 25 ms to observe the effects of partial recovery of the RyR channel.

As seen in Figure 13, the decrease in interval between stimuli causes partial recovery of [*Ca*^2+^] level in the jSR which manifests in increasing levels of unbound version of the protein calsequestrin, which tends to bind with triadin. As more triadin binds to calsequestrin, there is less left to bind to the luminal side of RyR to activate the channel for CICR. This signaling sequence mediated by the luminal sensor via the interaction of proteins calsequestrin and triadin, allows only a partial SR *Ca*^2+ ^release based on the degree of SR *Ca*^2+ ^content recovery. This is an important feature that helps restore the jSR *Ca*^2+ ^content at the end of every cycle. The RyR channel in our model has an absolute refractory period of 75 ms and a relative refractory period of 250 ms (Figure 13C). These results are consistent with the refractory period measurements by Sobie et al [106] on rat ventricular myocytes. When the RyR channel is in the absolute refractory period, [*Ca*^2+^]*_RyR _*drops to a level much below what is caused by a sparklet (trigger *Ca*^2+^) and hence robustly avoids re-excitation. However, when the RyR channel is in the relative refractory period, a partial release (Figure 13C) is possible, despite the RyR receptors affinity for release being low.

**Figure 14:**
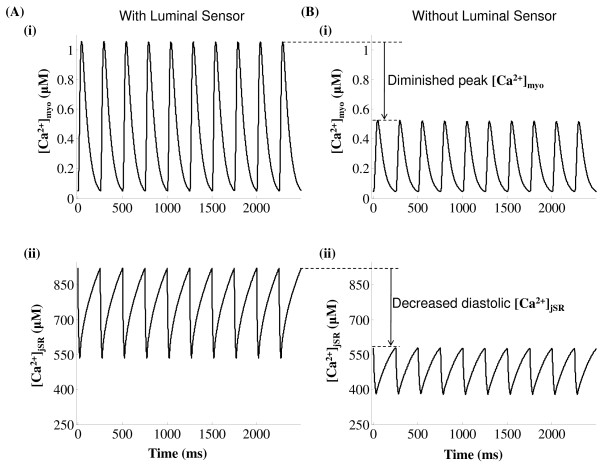
**Role of Luminal sensor**. Figure 14: Application of a train of voltage clamp pulses (amplitude -40 to 10 mV; duration 50 ms) for a period of 2.5 sec. Pulse repetition rate is 4.0 Hz. Luminal sensor is intact in A and removed in B. Both A and B are steady state responses to voltage clamp stimulation. A-(i) [*Ca*^2+^]*_myo _*transients with normal amplitude. A-(ii) Sustained SR filling due to robust RyR inactivation. B-(i) Diminishing [*Ca*^2+^]*_myo _*transients indicate poor SR filling. B-(ii) Lowered [*Ca*^2+^]*_jSR _*levels indicating inadequate refilling of SR stores due to lack of RyR refractoriness.

Figures 14A and 14B show the steady state [*Ca*^2+^]*_myo _*and [*Ca*^2+^]*_jSR _*predicted by the model, with and without a functioning luminal sensor, respectively. The stimulation protocol used is a pulse train of amplitude (-40 mv to 10 mv), duration (50 ms) and frequency of 4.0 Hz which is applied for a period of 2.5 sec. The value of the luminal control of the RyR channel in allowing adequate SR filling can be seen in Figure 14 where, in the presence of the luminal sensor the cell maintains a normal cytosolic *Ca*^2+ ^transient (Figure 14A-i). This is made possible due to sufficient SR filling (Figure 14A-ii) as a result of RyR refractoriness. However, inactivation of the RyR channel is also known to depend on the high local *Ca*^2+ ^concentration consequential to its own *Ca*^2+ ^release [51]. The lack of a luminal sensor forces the RyR channel to solely rely on *Ca*^2+ ^dependent inactivation mechanism. The resulting inadequate RyR inactivation depletes diastolic [*Ca*^2+^]*_jSR _*level (Figure 14B-ii) causing the cytosolic *Ca*^2+ ^transient to diminish to new lowered steady state values (shown in Figure 14B-i). The absence of the luminal sensor is modeled by setting the value of the luminal sensing variable ('var' in the model) to a level consistent with it's normal value under 4 Hz, voltage clamp stimulation. This mimics the case where the luminal sensor is insensitive to changes in [*Ca*^2+^]*_jSR _*and hence non-functional. It is important to note that the decrease (37.07%) in diastolic SR level (as indicated in Figure 14B-ii) is due to the lack of a luminal control on the RyR channel resulting in a reduced rate of RyR recovery which in turn reflects in a compromised SR filling rate. Hence, the presence of a luminal sensor which monitors the SR *Ca*^2+ ^content, is key to long term *Ca*^2+ ^stability in the cell.

### CICR modulation by the jSR Ca^2+ ^content

From the refractory characteristics of the RyR channel, it is evident that the RyR release is strongly dependent on the jSR *Ca*^2+ ^content. In fact, the SR *Ca*^2+ ^release through the RyR channel depends on: (a) the amount of trigger *Ca*^2+ ^entering by means of the *I_Ca,L,TT _*channel (b) the concentration gradient of *Ca*^2+ ^between the jSR and the mouth of the RyR channel in the dyadic space and (c) the open probability of the RyR channel modulated by the interaction between the luminal sensor and the RyR protein. The SR *Ca*^2+ ^released also inactivates the trigger current *I_Ca,L,TT _*(as shown in Figure 7), hence causing an indirect self-inhibition of RyR release.

**Figure 15:**
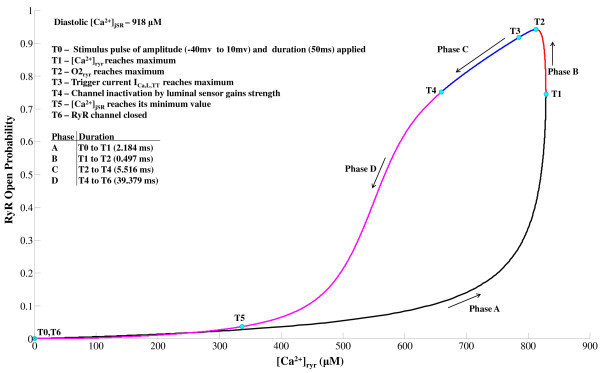
**Ryanodine receptor open probability**. Figure 15: RyR open probability modulation by concentration at the mouth. The pre-release diastolic [*Ca*^2+^]*_jSR _*is 918 *μ*M. The stimulation protocol used is a pulse of amplitude (-40 mv to 10 mv) and duration (50 ms). Phase A - As a result of excitation by the trigger current, [*Ca*^2+^]*_ryr _*initially increases rapidly with a small accompanying rise in RyR open probability followed by steeper increase to peak [*Ca*^2+^]*_ryr _*with a large increase in RyR open probability.; Phase B - RyR open probability increases to its maximum value at a declining rate reflecting a falling [*Ca*^2+^]*_ryr _*level and onset of *Ca*^2+ ^induced self-inhibition; Phase C - Decreasing *Ca*^2+^*_dyad _*and continued *Ca*^2+ ^induced self-inhibition, begins the channel recovery process; Phase D - Decreasing [*Ca*^2+^]*_jSR _*forces a strong RyR inactivation via the luminal sensor hence robustly closing the channel.

Figure 15 shows a phase plot of RyR open probability (*O*2*_ryr_*) versus the *Ca*^2+ ^concentration at the mouth of the RyR channel in the dyadic space [*Ca*^2+^]*_ryr _*constructed from model-generated data for a pulse of amplitude -40 mv to 10 mv and a duration of 50 ms. The pre-release diastolic [*Ca*^2+^]*_jSR _*is 918 *μ*M. Following excitation by the trigger current during phase A, the RyR channel begins to open, allowing *Ca*^2+ ^flux into the dyad and elevating [*Ca*^2+^]*_ryr _*at the mouth of the RyR channel. The rate of opening of the channel is *Ca*^2+ ^dependent; hence as [*Ca*^2+^]*_ryr _*increases, RyR open probability increases first slowly and then ever more rapidly. With this onrush of *Ca*^2+^, [*Ca*^2+^]*_ryr _*rapidly equilibrates with the [*Ca*^2+^]*_jSR_*, reducing and eventually abolishing the concentration gradient across the channel. [*Ca*^2+^]*_ryr _*soon reaches its maximum value (T1 in Figure 15) and begins to track the decrease in [*Ca*^2+^]*_jSR_*, there being no further significant concentration gradient existing between [*Ca*^2+^]*_jSR _*and [*Ca*^2+^]*_ryr _*at the mouth of the RyR channel. During phase B, the rate of rise in RyR open probability decreases due to decreasing [*Ca*^2+^]*_ryr _*and the onset of self-inhibition due to the large values of *Ca*^2+ ^concentration at the mouth forcing the RyR open probability to attain its maximum value (T2 in Figure 15). This is followed by phase C, where the RyR open probability begins to decrease slowly, initiating channel recovery due to decreasing overall [*Ca*^2+^]*_dyad _*levels as a result of (a) *Ca*^2+ ^fluxing out of the dyad into the cytosol, (b) lack of a drive from SR release due to drastically diminished *Ca*^2+ ^gradient at the mouth of the RyR channel and (c) continued *Ca*^2+ ^induced self-inhibition. Decreasing levels of free *Ca*^2+ ^in the jSR force a strong RyR inactivation via the luminal sensor (beginning at T4 and continuing throughout phase D; Figure 15), which in turn causes a sharp decline in RyR open probability. The minimum value of [*Ca*^2+^]*_jSR _*occurs at T5, and the channel is ultimately closed at T6 (Figure 15). The luminal sensor mediated inactivation assists in robust [*Ca*^2+^]*_jSR _*recovery.

#### Cytosolic peak [Ca^2+^] dependence on SR Ca^2+ ^content

**Figure 16:**
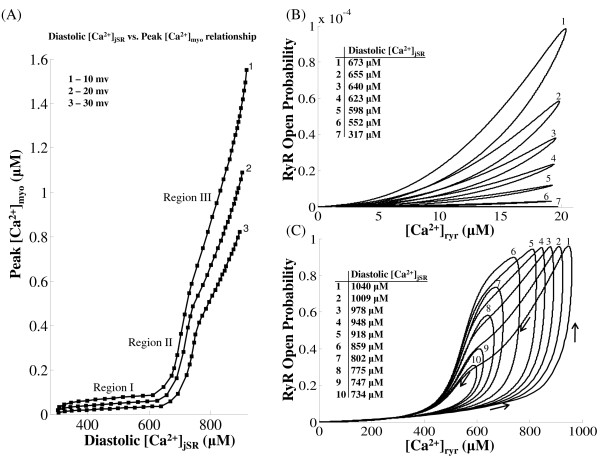
**Peak [Ca^2+^]_myo _vs [Ca^2+^]_jSR_**. Figure 16: (A) Relation between the peak of the cytosolic *Ca*^2+ ^transient and the diastolic [*Ca*^2+^]*_jSR_*. Region I: The peak RyR open probability is very small owing to the low SR *Ca*^2+ ^content. This causes the peak *Ca*^2+ ^transient to be very small. Region II: Increasing SR *Ca*^2+ ^content causes an increase in the peak RyR open probability and hence in the peak of the cytosolic *Ca*^2+ ^transient. Region III: A large rapid increase in the peak *Ca*^2+^*_myo _*is observed due to the large RyR *Ca*^2+ ^release associated with the high SR *Ca*^2+ ^content and saturation of RyR open probability. This model-generated result shows similarity to data in Figure 4, Diaz et al. [29].(B) Dependence of RyR open probability (O2*_RyR_*) on diastolic *Ca*^2+ ^*_jSR_*. Increasing pre-release diastolic *Ca*^2+^*_jSR _*results in increasing peak O2*_RyR_*. However, owing to the low SR *Ca*^2+ ^levels the peak open probability is very small. The stimulation protocol used is a pulse of amplitude -40 mv to 10 mv with a duration of 50 ms. (C) Dependence of RyR open probability (O2 *_RyR_*) on diastolic *Ca*^2+^*_jSR_*. Increasing diastolic *Ca*^2+^*_jSR _*results in a linearly increasing peak O2*_RyR_*. In the open probability saturation region the increase in peak cytosolic *Ca*^2+ ^transient is sustained by increasing the area contained by each loop as seen in traces numbered 5 to 1. The progress in time occurs along the arrow indicated on trace 1. The stimulation protocol used is a pulse of amplitude -40 mv to 10 mv with a duration of 50 ms.

Figure 16A shows three characteristic regions in the plot of the peak [*Ca*^2+^]*_myo _*vs [*Ca*^2+^]*_jSR_*. In Region I, where the jSR load is small, two things occur: (a) RyR release is reduced, as is the basal intracellular *Ca*^2+ ^concentration, and (b) *Ca*^2+ ^dependent inactivation of the *I_Ca,L,TT _*channel is reduced due to the lowered dyadic *Ca*^2+ ^concentration, which allows for greater *Ca*^2+ ^entry into the dyadic space via the *I_Ca,L _*channel. This increase in trigger current results only in a very small increase in *Ca*^2+ ^release, because of the inhibition of the Ry-sensitive channel caused by the luminal sensor in response to low SR *Ca*^2+ ^content. Thus, these factors cumulatively result in only a small linear increase in the peak [*Ca*^2+^]*_myo _*with increasing SR *Ca*^2+ ^content in Region I. The behavior in Region I corresponds to traces 7-1 in Figure 16B (shows phase plots of *O*2*_ryr _*vs [*Ca*^2+^]*_ryr _*for different steady state [*Ca*^2+^]*_jSR _*concentrations), where the peak RyR open probability shows very small gradual increase with increasing levels of diastolic pre-release [*Ca*^2+^]*_jSR _*for a constant trigger *Ca*^2+ ^input via *I_Ca,L_*. The steady state diastolic [*Ca*^2+^]*_jSR _*levels being low, the maximum RyR open probability values attained are low. Although the peak [*Ca*^2+^]*_ryr _*does not increase substantially as the diastolic [*Ca*^2+^]*_jSR _*levels increase from 317*μ*M to 673*μ*M, the area enclosed by the [*Ca*^2+^]*_ryr _*- *O*2*_ryr _*loop (which indicates the amount of SR *Ca*^2+ ^released into the dyad) increases exponentially due to increasing peak RyR open probability combined with the increasing difference between rate of activation and inactivation with rate of activation increasing faster than the rate of channel inactivation. In Region II of Figure 16A, increasing SR *Ca*^2+ ^content begins to translate into a substantial increase in peak [*Ca*^2+^]*_myo_*. This is because increasing diastolic [*Ca*^2+^]*_jSR _*causes a significant increase in the peak RyR open probability, as shown in Figure 16C, which translates into a commensurate increase in the peak of the cytosolic *Ca*^2+ ^transient. The inhibiting role of the luminal sensor is relieved with building [*Ca*^2+^]*_jSR _*levels. The nonlinearity observed between the diastolic [*Ca*^2+^]*_jSR _*levels 673*μ*M (Figure 16B) and 734*μ*M (Figure 16C) at the transition between Regions I and II corresponds to the existence of a threshold characteristic for RyR release [107,108]. As the diastolic [*Ca*^2+^]*_jSR _*levels increase beyond 734*μ*M, the area enclosed by the [*Ca*^2+^]*_ryr _*- *O*2*_ryr _*loop increases substantially reaching a maximum value at [*Ca*^2+^]*_jSR _*level of 948*μ*M not only due to a significant increase in both peak [*Ca*^2+^]*_ryr _*and peak *O*2*_ryr _*but also due to a rapid increase in rate of RyR activation resulting in a much larger difference in rate of activation and inactivation. Region III exhibits a large rapid increase in peak [*Ca*^2+^]*_myo _*due to the large RyR *Ca*^2+ ^release associated with the high SR *Ca*^2+ ^content. However, it is important to note that this is not a result of increasing peak RyR open probability, which begins to plateau for steady state [*Ca*^2+^]*_jSR _*values larger than 850*μ*M (traces 6-1 in Figure 16C). The continuing rapid increase in peak cytosolic *Ca*^2+ ^concentration in region III is a combined result of: (a) increased release owing to large SR *Ca*^2+ ^content; (b) large values for saturated RyR open probability supported by an increase in the area contained by each loop as seen in traces 6-1 of Figure 16C; and (c) saturated operation of the SERCA pump which acts as a predominant buffer in restoring the *Ca*^2+ ^concentration levels in the cytosol after RyR release [77]. As the diastolic [*Ca*^2+^]*_jSR _*levels increase beyond 948*μ*M, the area enclosed by the [*Ca*^2+^]*_ryr _*- *O*2*_ryr _*loop begins to decrease despite increasing peak [*Ca*^2+^]*_ryr _*due to saturation of the peak RyR open probability and increasing rate of RyR channel inactivation due to large values of local *Ca*^2+ ^concentration ([*Ca*^2+^]*_ryr_*) assisting in faster recovery [51]. This model generated relationship between the peak [*Ca*^2+^]*_myo _*and the pre-release [*Ca*^2+^]*_jSR _*agrees with Figure 4 in Diaz et. al. [29].

As seen in traces 1-10 of Figure 16C, during the initial activation of a RyR channel (linear region of phase A in Figure 15), at any specific [*Ca*^2+^]*_ryr _*the RyR open probability takes a lower value despite a higher steady state diastolic [*Ca*^2+^]*_jSR _*level. This decreasing slope with increasing diastolic, pre-release [*Ca*^2+^]*_jSR _*reflects a faster rate of rise in [*Ca*^2+^]*_ryr _*for a larger pre-release SR content with a very small accompanying increase in the RyR open probability.

### Ca^2+ ^Release and its Effect on I_Ca,L_

#### Caffeine

Adachi-Akahane et al. [71] investigated *I_Ca,L_*-induced *Ca*^2+ ^release in rat ventricular myocytes in the presence and absence of caffeine, which altered [*Ca*^2+^]*_myo_*. Using their protocols and data as a guide, we modeled the effect of caffeine on jSR *Ca*^2+ ^release and its subsequent effect on *I_Ca,L _*. Specifically, a Boltzman relationship was adopted to simulate the binding of caffeine to the ryanodine receptor increasing channel conductance, which in turn enhances jSR *Ca*^2+ ^release. The process is assumed to be dose-dependent, with an open probability of 0.5 at a caffeine concentration of 5 mM (Appendix A1, Eq. 86).

**Figure 17:**
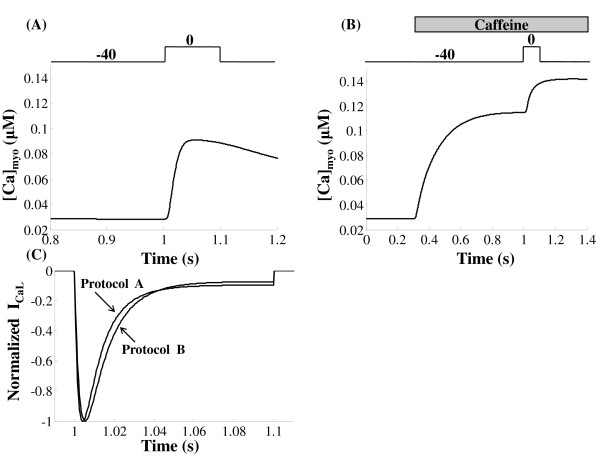
**Effects of the application of caffeine**. Figure 17: Model-simulated effects of the application of caffeine on *I*_*Ca*,*L*,*TT *_and [*Ca*^2+^]*_i _*in a cell dialyzed with 2 mM Fura-2. Changes in [*Ca*^2+^]*_myo _*resulting from: (A) application of a simple depolarizing pulse from -40 mV to 0 mV, and (B) the same depolarizing pulse following pre-exposure to caffeine (5 mM). Panel (C) compares the *I_Ca,L _*waveforms associated with these protocols. This model-generated result shows similarity to data in Figure 3, Adachi-Akahane et al. [71].

Figures 17A and 17B show model-generated [*Ca*^2+^]*_myo _*responses in myocytes dialyzed with 2 mM Fura-2, where a simple voltage clamp pulse (-40 mV to 0 mV) is applied in the presence and absence of caffeine. *I_Ca,L _*waveforms associated with these two protocols are shown in Figure 17C. In protocol A, conducted at normal rest levels of [*Ca*^2+^]*_myo _*(10 nM), the depolarizing test pulse to 0 mV from the holding potential of -40 mV for 0.1 sec fully activates *I_Ca,L _*, which in turn triggers a rapid but small amplitude *Ca*^2+ ^transient (a rise from 30 nM to 95 nM as shown in Figure 17A. Protocol B starts with an application of 5 mM caffeine for a period of 1.0 sec (open state probability of RyR channel is 0.5), followed by the same depolarizing test pulse applied at 1.0 sec. The conditioning caffeine pulse induces a [*Ca*^2+^]*_myo _*transient rising from a resting value of 30 nM to a peak value of 110 nM (Figure 17B, first peak -note the different timescale). The short depolarizing pulse activates *I_Ca,L_*, which triggers a much smaller [*Ca*^2+^]*_myo _*transient via CICR (30 nM, Figure 17B, second peak). This *I_Ca,L_*-evoked *Ca*^2+ ^transient in response to the voltage pulse is smaller in amplitude than control (Figure 17A), due to: (a) reduction in RyR release due to depletion in [*Ca*^2+^]*_jSR _*([*Ca*^2+^]*_jSR _*drops from 1.25 mM to 0.25 mM with caffeine application).; and (b) the strong competing role of the luminal RyR sensor in inactivating the RyR channel in response to the post-caffeine, low SR content. The *I_Ca,L_*-inactivation characteristics undergo a change as shown in Figure 17C, where trace B inactivates more slowly compared to the control *I_Ca,L _*(trace 1). After exposure to caffeine, the overall amplitude of [*Ca*^2+^]*_dhp _*during release does not increase as much as in the control case, since jSR *Ca*^2+ ^release is reduced. Consequently, *Ca*^2+ ^dependent inactivation (during release) of *I_Ca,L _*after caffeine application is not as strong as that in the control case. However, the baseline level of *Ca*^2+ ^concentration at the mouth of the DHP channel ([*Ca*^2+^]*_dhp_*) is elevated following caffeine application, causing sustained inactivation resulting in the crossover of the current trace in Figure 17C. This is consistent with the observation of Cens et al. [41], that *Ca*^2+ ^induced inactivation is a result of calmodulin mediated sensing of the local *Ca*^2+ ^concentration.

#### Thapsigargin

**Figure 18:**
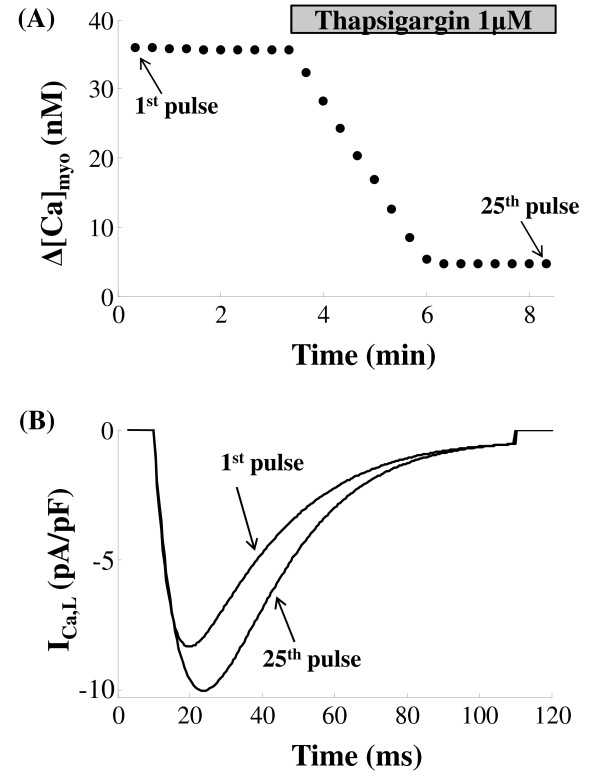
**Effects of the application of Thapsigargin**. Figure 18: In rat ventricular myocytes dialyzed with 2 mM Fura-2, thapsigargin gradually abolishes [*Ca*^2+^]*_myo _*transients and decreases the rate of inactivation of *I_Ca,L_*. This result is generated by the model, in response to a pulse train of amplitude (-40 mV to 0 mV), duration (0.1 sec) and frequency of 0.05 Hz. (A) Time course of the effect of thapsigargin on Δ[*Ca*^2+^]_i_. (B) L-type *Ca*^2+ ^currents at the times indicated in (A). This model-generated result shows similarity to data in Figure 11, Adachi-Akahane et al. [71]

Analogous to the experimental protocol of Adachi-Akahane et al. [71] dealing with thapsigargin blockade of the SERCA pump (their Figure 9), we apply a series of voltage pulses (-40 mV to 0 mV, duration 0.1 sec, frequency 0.05 Hz) to our cell model, modified by blockade of the SERCA pump and addition of 2 mM Fura-2. Figure 18 shows that when *Ca*^2+ ^uptake is blocked, SR *Ca*^2+ ^content declines, as does the jSR *Ca*^2+ ^release with each voltage pulse (Figure 18A). Considerable time is consumed after thapsigargin application, before SR *Ca*^2+ ^content is finally exhausted (8-10 beats at 0.05 Hz; Figure 18A). As the magnitude of the [*Ca*^2+^]*_myo _*transient decreases, the [*Ca*^2+^]*_dhp _*dependent inactivation of *I_Ca,L _*slows (Figure 18B), resulting in an enhanced peak.

For a given clamp amplitude, a decrease in jSR *Ca*^2+ ^content decreases *Ca*^2+ ^release, producing decreased *Ca*^2+ ^concentration at the dyadic side of the DHP-sensitive channel. Consequently, a smaller amount of calcium-calmodulin complex (*Ca*_4_*CaM*) is activated, leading to a lower degree of *Ca*^2+ ^dependent inactivation. Thus, *I_Ca,L _*peak is enhanced, as shown in Figure 18B, increasing the area under the waveform, corresponding to an increased amount of *Ca*^2+ ^entering the cell. This inverse relationship between jSR *Ca*^2+ ^content and the amount of trigger *Ca*^2+ ^entering the dyadic space for a given clamp voltage is the essence of the feedback mechanism maintaining the efficacy of CICR. This mechanism is operational even in the presence of high concentrations of cytoplasmic *Ca*^2+^-buffers, since the small dimensions of the dyadic space allow jSR released *Ca*^2+ ^to reach its destination (i.e., the DHP receptor) before being captured by the buffers [109].

### Effect of modulation of [Ca^2+^]_o_

**Figure 19:**
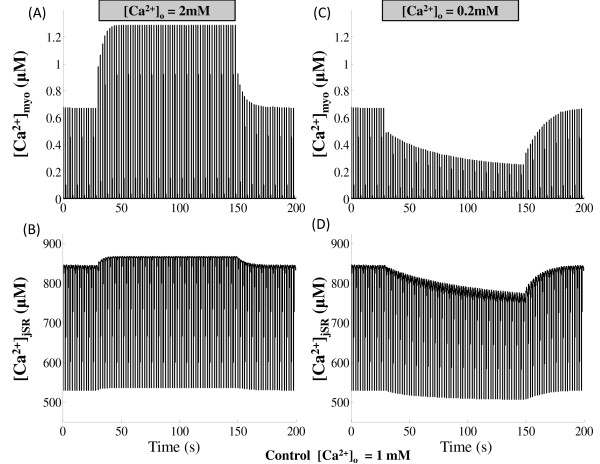
**Effects of modulating [Ca^2+^]_o_**. Figure 19: The effects of changing external *Ca*^2+ ^concentration on cytoplasmic *Ca*^2+ ^and SR *Ca*^2+ ^content. The stimulation protocol used is a pulse train of amplitude (-40 mv to 0 mv), duration (100 ms) and frequency of 0.5 Hz. (A) Following sudden rise in [*Ca*^2+^]*_o _*the instantaneous increase in [*Ca*^2+^]*_myo _*occurs due to more Ca entry, followed by a gradual staircase rise due to the the change in SR content; (B) Changes in [*Ca*^2+^]*_myo _*forced by a sudden decrease in [*Ca*^2+^]*_o_*; (C) Changes in [*Ca*^2+^]*_jSR _*due to increased [*Ca*^2+^]*_o_*; (D) Changes in [*Ca*^2+^]*_jSR _*due to reduced [*Ca*^2+^]*_o_*. This model-generated result shows similarity to data in Figure 2, Diaz et al. [110].

Increase in [*Ca*^2+^]*_o _*causes an increase in entry of extracellular *Ca*^2+ ^into the cell via the *I_Ca,L _*channel. This results in an increase in SR *Ca*^2+ ^content, as shown in Figure 19B. Aided by an increase in trigger *Ca*^2+ ^as well as a larger SR *Ca*^2+ ^content, the RyR release is enhanced. This increase in SR release assisted by impaired *Na*^+^/*Ca*^2+ ^exchange due to elevated [*Ca*^2+^]*_o _*manifests in an increase in the peak *Ca*^2+ ^concentration levels in the cytosol, as shown in Figure 19A. Similarly, a decrease in extracellular *Ca*^2+ ^levels causes a decrease in trigger *Ca*^2+ ^available to cause CICR, which results in a decrease in peak [*Ca*^2+^] levels in the cytosol, as shown in Figure 19C. Reduced *Ca*^2+ ^entry into the cell via *I_Ca,L_*, translating into decreased availability of post-release *Ca*^2+ ^in the cytosol, causes reduction in SR filling via the bi-directional SERCA pump. The corresponding drop in the SR *Ca*^2+ ^content is evident in Figure 19D. Although, our model-generated results show similarity to data in Figure 2, Diaz et al. [110], our result in Figure 19D does not confirm an increase in SR *Ca*^2+ ^content with a decrease in [*Ca*^2+^]*_o _*as reported in Figure 2B, Diaz et al. [110]. On the contrary, they agree with the data in Figure 5, Diaz et al. 1997 which shows a decrease in free diastolic steady-state SR *Ca*^2+ ^content with decrease in [*Ca*^2+^]*_o _*in cells not showing spontaneous release. In this study, we have attempted to isolate the effects of changes in [*Ca*^2+^]*_o _*on intracellular *Ca*^2+ ^levels by ensuring that there are no voltage dependent effects on *Ca*^2+ ^transport via the *I_Ca,L _*channel, *Na*^+^/*Ca*^2+ ^exchanger or the plasma membrane *Ca*^2+ ^ATPase pump. The simulation protocol used in our study (reproduced from Diaz et al. [110]),is a pulse train of amplitude (-40 mv to 0 mv), duration (100 ms) and a frequency of 0.5 Hz. However, it is important to note that, for any constant extracellular *Ca*^2+ ^concentration, in response to a voltage clamp pulse train, increasing clamp potential (*>*10 mv) results in a decrease in steady state SR *Ca*^2+ ^content as a result of the dominating effect of reduced *Ca*^2+ ^entry via the *I_Ca,L _*channel despite a decrease in net *Ca*^2+ ^extrusion via the *Na*^+^/*Ca*^2+ ^exchanger per cycle.

### Calcium balance under conditions of repetitive stimulation

**Figure 20:**
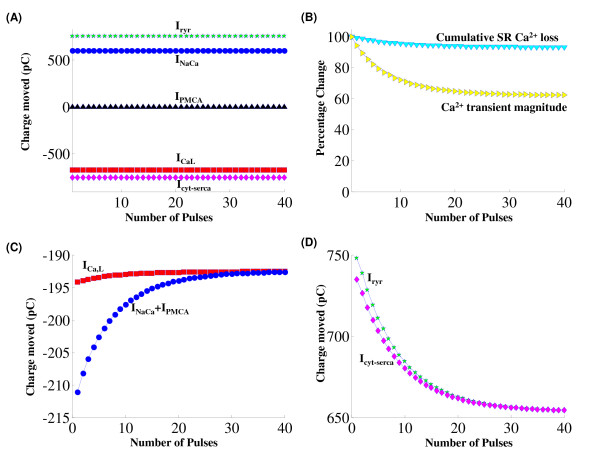
**Long term *Ca*^2+ ^stability during repetitive stimulation**. Figure 20: The stimulation protocol used is a pulse train of amplitude (-40 mv to 10 mv) and frequency of 0.33 Hz.(A) Integrals of external and internal *Ca*^2+ ^currents on stimulation with repetitive long pulses (800 ms); (B) Percentage of changes in [*Ca*^2+^]*_myo _*peak magnitude and cumulative SR *Ca*^2+ ^loss on stimulation with repetitive short pulses (100 ms); (C) Integrals of external *Ca*^2+ ^currents with short-pulse stimulations (100 ms); (D) Integrals of internal *Ca*^2+ ^currents with short-pulse stimulations (100 ms). This model-generated result shows similarity to data in Figure 3 and Figure 5, Negretti et al. [111].

Our model exhibits long term *Ca*^2+ ^stability. Analogous to the experimental protocols of Negretti el al. [111], we studied the dynamic aspects of the calcium balance in the cell model by subjecting it to repetitive voltage clamps of different durations. For stable steady-state operation, *Ca*^2+ ^entry into the cytosol via *I_Ca,L _*must exactly balance *Ca*^2+ ^efflux. Changing the rate or pattern of stimulation can have significant effects on the cell's cytosolic *Ca*^2+ ^balance and subsequent contractile response [16]. To demonstrate *Ca*^2+ ^balance in our model, long (Figure 20A) or short (Figure 20B, C and 20D) voltage clamp pulses were applied at a selected repetition frequency, and the resultant changes in [*Ca*^2+^]*_myo _*were correlated with sarcolemmal and SR *Ca*^2+ ^fluxes. Analogous to the protocol of Negretti et al. [111], pulse trains were applied from a holding potential of -40 mV to +10 mV, with durations of either 800 ms (long-pulse) or 100 ms (short-pulse) at a frequency of 0.33 Hz, for a total test duration of 2 minutes (40 pulses). In analyzing the *Ca*^2+ ^balance, the amount of electric charge Q transferred per pulse was calculated according to the equation: $\int_{0}^{T}I\left(t\right)dt$, where I(t) can be *I_Ca,L _, I_NaCa_, I_PMCA_, I_ryr_, I_cyt,serca_*, and T is 1 cycle duration (3 ms for 0.33 Hz stimulation).

Numerical integration was carried out with respect to steady-state levels, hence only activating currents and leakage currents were considered (background *Ca*^2+ ^current is neglected). The results of the *Ca*^2+ ^balance are presented in a manner where relative *Ca*^2+ ^fluxes can be easily compared. We note that in evaluating the integral of the combined exchanger current *I_NaCa_*, the integral is multiplied by a factor of 2 to account for the fact that the exchanger stoichiometry is 3*Na*^+ ^per *Ca*^2+ ^(the exchanger transports 1 net charge per *Ca*^2+^, whereas all other *Ca*^2+ ^currents transport 2 charges per *Ca*^2+^).

#### Long-pulse protocol

During repetitive long-pulse (800 ms) stimulation, transient *Ca*^2+ ^fluxes cross the sarcolemmal and SR membranes in both directions (inward (into the cytoplasm) and outward). However, [*Ca*^2+^]*_myo _*returns to control levels by the end of the long-pulse stimulation protocol. The long-pulse stimulation protocol thus serves as a means of studying the steady-state balance of *Ca*^2+ ^influx and efflux to and from the cytoplasm, under control conditions. In contrast, this *Ca*^2+ ^balance is not present in the short-pulse protocol, and [*Ca*^2+^]*_myo _*is elevated at pulse termination. Our long-pulse simulations show that: (1) the magnitude of individual *Ca*^2+ ^transients do not change during the 40 pulse sequence; (2) the peak calcium release current (*I_ryr_*) has a constant magnitude; and (3) the occupancy of the calsequestrin *Ca*^2+ ^buffer in the jSR compartment is reduced by 15% as a result of *Ca*^2+ ^release during each cycle. Although *Ca*^2+ ^fluxes cross the sarcolemma and SR membranes in either direction, the integral of each of these currents (indicating charge transfer) over the pulse repetition interval is a constant. Figure 20A shows this well, in that, the charge transfer for each model current (large or small) is a constant, indicating no net loss of *Ca*^2+ ^nor consecutive-pulse *Ca*^2+ ^depletion (or augmentation) in the jSR compartment under the long-pulse protocol. In Figure 20, we refer to *Ca*^2+ ^influx and efflux through the bounding cell membrane (sarcolemma (SL) and T-tubule) as "external fluxes" (i.e., *I_Ca,L_*, *I_NaCa_*, and *I_PMCA_*), whereas SR membrane *Ca*^2+ ^fluxes (*I_ryr_*, *I_cyt,serca_*) are called "internal fluxes." By convention, inward fluxes are negative (e.g. *I_Ca,L_*) and outward positive (e.g., *I_NaCa _*and *I_PMCA_*). Figure 20A shows that the integrated external fluxes sum to zero. The average *Ca*^2+ ^charge entering the cell via *I_Ca,L _*is 603.28 pC (pico-coulomb), and the sum of averaged *Ca*^2+ ^charges extruded from the cell via *I_NaCa _*and *I_PMCA _*is 603.28 pC (602.2 pC by *I_NaCa _*and 1.08 pC by *I_PMCA_*. Figure 20A also shows the magnitudes of the integrated internal fluxes, which sum to zero as well. In calculating inward and outward *Ca*^2+ ^fluxes relative to the SR lumen, we consider an inward flux (e.g., *I_cyt,serca_*) negative and an outward flux positive (e.g., *I_ryr_*). The integral of *I_ryr _*is 751.8 pC, compared with integral of the *I_Ca,L _*trigger current (603.28 pC). The calcium gain of CICR calculated in this way is low owing to the increased entry of *Ca*^2+ ^via the trigger current *I_Ca,L _*for a prolonged duration of 800 ms compared to 50 ms previously. The integral of SR *Ca*^2+ ^uptake is 751.8 pC. These two internal component currents yield flat integral values during long-pulse stimulations and sum to zero.

#### Short-pulse protocol

Decreasing the length of the depolarizing pulse to 100 ms (short pulse) has a pronounced effect on the recovery of contraction (Figure 20B, C and 20D). Compared with the relatively flat amplitude of [*Ca*^2+^]*_myo _*observed with stimulation by long pulses, short pulse stimulation produces a negative staircase (Figure 20B) in agreement with the data (Negretti et al. [111]), wherein the peak magnitude decays exponentially to a steady state level of 60% of the initial value. Figure 20B also shows that the model predicts a similar exponential decay in jSR *Ca*^2+ ^concentration [*Ca*^2+^]*_jSR_*. Both of these indicators show a net loss of *Ca*^2+ ^from the cell. Figure 20C and 20D show the transient charge movements associated with the external and internal *Ca*^2+ ^currents respectively during application of the short-pulse train, which are indicative of an elevated *Ca*^2+ ^load and resultant *Ca*^2+ ^imbalance when the pulse train is first applied. Balance is achieved, but at new lower values of [*Ca*^2+^]*_myo _*and [*Ca*^2+^]*_jSR _*. For convenient comparison, all internal fluxes are made positive and all external fluxes negative. Figure 20C shows that *I_NaCa _*plays a significant role in forming the transient response seen in the short-pulse protocol in extrusion of excess *Ca*^2+ ^load on the cell. The decreasing amplitude of the *Ca*^2+ ^transient causes a decreased LSR uptake current (*I_cyt,serca_*), which in turn causes a decrease in [*Ca*^2+^]*_jSR _*resulting in decreased release (*I_ryr_*). With successive pulses, *I_cyt,serca _*and *I_ryr _*decline further, mirroring the exponential decline in *I_NaCa _*toward balance. At steady state, the external and internal fluxes reach the new equilibrium, where the sum of the integrals of external currents and the sum of the integrals of internal currents are zero.

#### Long-term calcium stability at higher pacing rates

Most VC experiments on cardiac cells are usually conducted at low stimulation rates. Our simulations of typical VC experiments at these low rates all exhibit long term calcium stability (Figures 17, 18, 19 and 20). However, our model can also exhibit sustained calcium balance at higher pacing rates. Figure 14A shows steady state cytosolic *Ca*^2+ ^transients in response to a repetitive 4 Hz (which is more physiological for a rat ventricular myocyte) voltage clamp stimulation lasting for 2.5 seconds. The sustained peak and basal *Ca*^2+ ^levels of [*Ca*^2+^]*_myo _*over this prolonged time frame are indicative of long tern *Ca*^2+ ^stability in the model. The corresponding [*Ca*^2+^]*_jSR _*profile over 2.5 seconds is also shown in Figure 14A, which indicates sustained SR filling owing to the luminal sensor mediated control of the RyR channel. The stimulation protocol used is a pulse train of amplitude (-40 mv to 10 mv), duration (50 ms) and frequency of 4.0 Hz. Tables 9 and 10 provide values for the initial conditions used in our model.

**Table 9:** Initial Conditions

State variable	Definition	Initial Value	
[*Ca*^2+^] *_myo_*	*Ca*^2+ ^concentration in the myoplasm	8.1027 × 10^-5 ^*mM*	

[*Ca*^2+^]*_jSR_*	*Ca*^2+ ^concentration in jSR	1.2677 *mM*	

[*Ca*^2+^]*_LSR_*	*Ca*^2+ ^concentration in LSR	1.3346 *mM*	

[*Na*^+^]*_myo_*	*Na*^+ ^concentration in myoplasm	16.746 *mM*	

[*Na*^+^]*_dyad_*	*Na*^+ ^concentration in dyadic space	16.321 *mM*	

[*Cs*^+^]*_myo_*	*Cs*^+ ^concentration in myoplasm	140.2154 *mM*	

[*Cs*^+^]*_dyad_*	*Cs*^+ ^concentration in dyadic space	140.2157 *mM*	

*O_c_*	Fractional occupancy of Calmodulin by *Ca*^2+^	0.033091	

*O_tc_*	Fractional occupancy of Troponin by *Ca*^2+^	0.016049	

*O_tmgc_*	Fractional occupancy of Troponin by *Mg*^2+ ^and *Ca*^2+^	0.321764	

*O_tmgmg_*	Fractional occupancy of Troponin by *Mg*^2+^	0.598385	

[*CaF *3]	Concentration of Fluo3-*Ca*^2+ ^complex	21.88721 *μM*	

*C*1*_ryr_*	Closed (resting) state of RyR channel	0.9990953	

*O*2*_ryr_*	Open (activated) state of RyR channel	1.03668 × 10^-9^	

*C*3*_ryr_*	Inactivated state of RyR channel	9.38711 × 10^-13^	

*C*1*_dhpr_*	Closed (resting) state of DHPR sensitive *Ca*^2+ ^channel	0.1673614	

*O*2*_dhpr_*	Open (activated) state of DHPR sensitive *Ca*^2+ ^channel	1.499173 × 10^-3^	

*O*3*_dhpr_*	Open (activated) state of DHPR sensitive *Ca*^2+ ^channel	3.300291 × 10^-3^	

*C*4*_dhpr_*	Closed (resting) state of DHPR sensitive *Ca*^2+ ^channel	7.478058 × 10^-8^	

*C*6*_dhpr_*	Closed (resting) state of DHPR sensitive *Ca*^2+ ^channel	7.478058 × 10^-8^	

*A*1*_ls_*	Fraction of Tr/J bound to RyR	0.6709816	

*I*2*_ls_*	Fraction of Tr/J bound to RyR and Calsequestrin	0.155258	

*I*3*_ls_*	Fraction of Tr/J bound only to Calsequestrin	0.0623695	

*B*6*_ls_*	Fraction Calsequestrin bound to *Ca*^2+^	0.619346	

[*Ca*^2+^]*_serca_*	Ca bound to the serca protein	36.0637 × 10^-5^*μM*	

*S*2*_dhpr_*	Fraction of IQ Motif bound to Ca_4_CaM	6.816729 × 10^-2^	

*PLB_dp_*	Fraction of unphosphorylated Phospholamban	7.684160 × 10^-2^	

*Ca*_2_*CaM*	2 *Ca*^2+ ^ions bound to C-terminus of CaM	34.56529	

*Ca*_4_*CaM*	4 *Ca*^2+ ^ions bound to C & N terminus of CaM	8.635052 × 10^-2^	

*CaMB*	Buffered CaM	7.563836 × 10^-2^	

*Ca*_2_*CaMB*	Buffered *Ca*_2_*CaM*	2.035086	

*Ca*_4_*CaMB*	Buffered *Ca *_4_*CaM*	1.288455 × 10^-6^	

*Ca*_4_*CaN*	CaN bound to 4 *Ca *^2+ ^ions	2.606246 × 10^-4^*μM*	

*CaMCaN*	CaN bound to CaM	4.348535 × 10^-3^*μM*	

*Ca*_2_*CaMCaN*	CaN bound to 2 *Ca*^2+ ^ions and CaM	1.419613 × 10^-1^*μM*	

*Ca*_4_*CaMCaN*	CaN bound to 4 *Ca*^2+ ^ions and CaM	3.473412*μM*	

[*Ca*^2+^]*_dyad_*	*Ca*^2+ ^concentration in the dyadic space	9.012 × 10^-5 ^*mM*	

Table 9: Initial conditions used for the state vector in order to solve the linearized system of ordinary differential equations

**Table 10:** Initial Conditions

State variable	Definition	Initial Value
*P*_1_	Fraction of inactive dephosphorylated CaMKII in *Ca*_2_*CaM *bound state	5.527608 × 10^-1^

*P*_3_	Fraction of active dephosphorylated CaMKII in *Ca*_4_*CaM *bound state	3.661260 × 10^-1^

*P*_6_	Fraction of active *Thr*^287^-autophosphorylated but *CaM *autonomous CaMKII	1.314410 × 10^-3^

*P*_5_	Fraction of active *Thr*^287^-autophosphorylated but Ca2CaM bound CaMKII	6.277911 × 10^-7^

*P*4	Fraction of active *Thr*^287^-autophosphorylated but Ca4CaM trapped CaMKII	9.121920 × 10^-8^

Table 10: Initial conditions used for the state vector in order to solve the linearized system of ordinary differential equations

### Secondary [Ca^2+^]_myo _transients induced by "tail currents"

**Figure 21:**
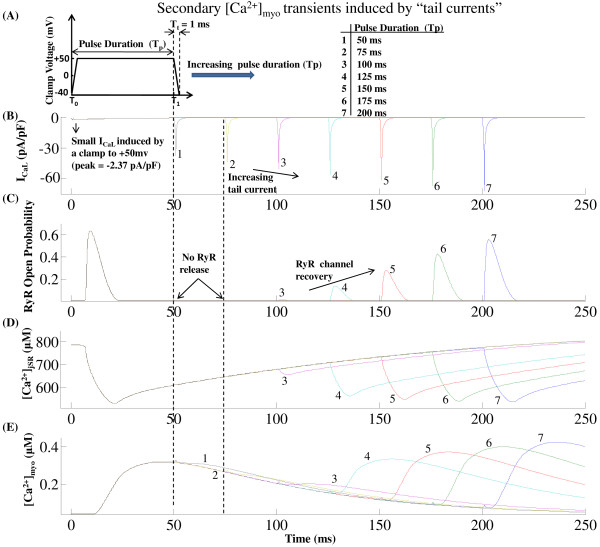
**Secondary [Ca^2+^]_myo _transients induced by "tail currents"**. Figure 21: The stimulation protocol used is a pulse of amplitude (-40 mv to +50 mv) with pulse duration *T_p _*increasing from 50 ms (trace 1) to 200 ms (trace 7) in steps of 25 ms. (A) A cartoon depicting the voltage clamp stimulus protocol where, pulse duration (*T_p_*) is varied to obtain traces 1-7 and the clamp potential transition time (*T_t_*) fixed at 1 ms. (B) *I_Ca,L _*tail currents elicited during repolarization from +50 mv to -40 mv. (C) Open probability of the RyR channel indicating gradual recovery from the refractory period (D) [*Ca*^2+^]*_jSR _*traces show increase in RyR release with increasing pulse duration as a result of adequate RyR channel recovery. (E) Cytosolic calcium transients indicate secondary RyR release caused by tail currents.

Our voltage clamp simulations have dealt with clamp voltages in the range -30 mV ≤ V ≤ 40 mV. Some experimental studies [100,2,112] however have employed even larger clamp voltages (40 mV ≤ V ≤ 60 mV) to explore the high voltage behavior of the DHP-sensitive calcium channel. Such large clamp voltages elicit a brief *Ca*^2+ ^influx called a "tail current", which has been shown to trigger RyR release and hence cause contraction during repolarization [100,2]. The secondary *Ca*^2+ ^transient induced by the "tail current" is a critical argument in favor of CICR as these "tail currents" are not observed in skeletal muscle where the membrane potential directly controls SR *Ca*^2+ ^release [112]. Our model reproduces this secondary *Ca*^2+ ^transient observed during the return to resting potential from a large clamp voltage (≥ 40 mV). Figure 21A shows a cartoon depicting the voltage clamp stimulation protocol used in our study where a pulse of amplitude (-40 mV to +50 mV) is employed with the pulse duration (*T_p_*; Figure 21A) increasing from 50 ms (trace 1) to 200 ms (trace 7) in steps of 25 ms. The clamp potential transition time (*T_t_*) is fixed at 1 ms. The peak of the "tail current" at the end of the pulse is 25 times larger than the peak of the *I_Ca,L _*current observed at the beginning of the pulse. This is in agreement with model generated VC data reported by Geenstein et al.([8]; Figure 8). Figure 21B shows that, with an increase in clamp pulse duration, there is a corresponding increase in the peak of the trail current (see traces 1-7) until the effect ultimately saturates. Corresponding to this increase in peak *I_Ca,L _*tail current, there is an increase in the open probability of the RyR *Ca*^2+ ^channel, with RyR *Ca*^2+ ^release indicated by the corresponding decrease in [*Ca*^2+^]*_jSR _*(panel C) and increase in [*Ca*^2+^]*_myo _*(panel D). We note from Figure 21C that for a shorter 50 ms VC pulse, there is no RyR *Ca*^2+ ^release although an *I_Ca,L _*tail current is produced. This tail current produced with the 50 ms pulse slightly augments the [*Ca*^2+^]*_myo _*transient just after its peak (trace 1, panel E), but there is no corresponding decrease in the [*Ca*^2+^]*_jSR _*transient (trace 1, panel D) indicating no RyR *Ca*^2+ ^release. At the end of this short pulse (50 ms), the RyR channel is in fact in its absolute refractory period. The small secondary increase in [*Ca*^2+^]*_myo _*transient as seen in data corresponding to trace 4 of Figure 7C is a result of the large *Ca*^2+ ^influx via the *I_Ca,L _*"tail current". During this phase, the *Na*^+^/*Ca*^2+ ^exchanger is biased to extrude *Ca*^2+ ^out of the dyad, and hence cannot be responsible for this secondary rise in the cytosolic *Ca*^2+ ^transient. As pulse duration is increased beyond 100 ms the "tail current" causes a gradual increase in RyR open probability (traces 3-7 in Figure 21) indicating progressive recovery from the refractory period. Minimum value of [*Ca*^2+^]*_jSR _*in traces 3-7 of Figure 21D show increase in SR *Ca*^2+ ^release with increasing pulse duration as a result of adequate RyR channel recovery and increasing *I_Ca,L _*"tail current". Cytosolic *Ca*^2+ ^transients shown in Figure 21E indicate "tail current" induced secondary RyR release for pulse duration ≥ 100 ms. It has also been previously reported [113] that recruitment of an additional population of previously 'silent' *Ca*^2+ ^channels could cause facilitation of tail currents at increasingly large clamp voltages with a time-dependence associated with the recruitment process. Our model not only accounts for the contribution of the already open channels (in states *O*2*_dhpr _*&*O*3*_dhpr_*) to the tail current during repolarization but also allows for the voltage- and time-dependent recruitment of 'silent' *Ca*^2+ ^channels modeled using a high voltage state *C*6*_dhpr_*. This study proves to be an adjunct to the study presented in Figure 13 in establishing the refractory nature of the RyR channel.

### Cytosolic Buffering

**Figure 22:**
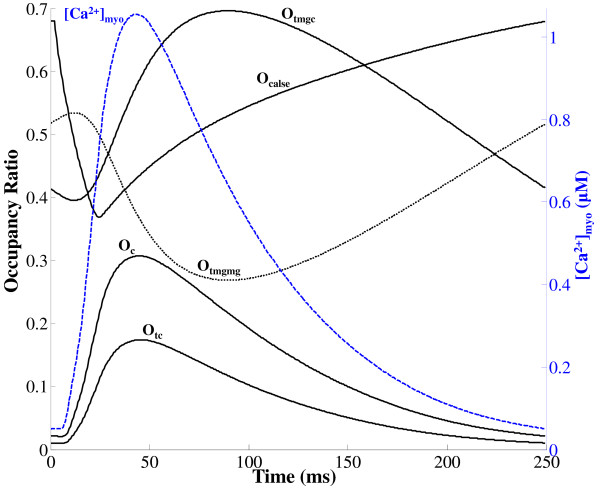
**Occupancy of Ca^2+ ^buffers**. Figure 22: Contributions of fractional occupancy of *Ca*^2+ ^buffers in SR and cytoplasm. Transfer from *O_c _*and *O_tc _*to *O_tmgc _*occurs as a result of the pulse stimulation. *O_tmgmg _*is shown as a dotted line as it does not reflect *Ca*^2+ ^binding. The corresponding [*Ca*^2+^]*_myo _*is overlayed (note the different axis)

In our model, cytosolic buffering is attributed to several factors including: (a) calmodulin (CaM); (b) the *Ca*^2+^-specific (Tc) troponin binding site; (c) the *Ca*^2+ ^- *Mg*^2+ ^competitive troponin binding site; and (d) the fluorescent indicator dye Fluo3 used to detect changes in [*Ca*^2+^]*_myo_*. The effects of the component buffers in helping to maintain a low [*Ca*^2+^]*_myo _*are shown in Figure 22 in terms of occupancy functions, such as *O_C _*(fractional occupancy of calmodulin by *Ca*^2+^), *O_tc _*(fractional occupancy of troponin-Ca sites by *Ca*^2+^), *O_tmgc _*(fractional ocupancy of troponin-Mg sites by *Ca*^2+^), *O_tmgmg _*(fractional occupancy of troponin-Mg sites by *Mg*^2+^) in the cytosol, and *O_calse _*(fractional occupancy of calsequestrin by *Ca*^2+^) in the jSR *Ca*^2+ ^release compartment. Figure 22 shows that when the Ry-sensitive *Ca*^2+ ^release channel is triggered, the jSR releases *Ca*^2+ ^and the occupancy *O_calse _*in the jSR, declines from 68% to 37%. *Ca*^2+ ^release from the jSR induces fast *Ca*^2+ ^binding by calmodulin and troponin in the cytosol, as represented by the increases of *O_c _*(0.29) and *O_tc _*(0.16) in Figure 22. Interactions between *Ca*^2+ ^and the troponin-Mg sites result in an increase in occupancy of these sites by *Ca*^2+ ^(*O_tmgc_*), and a decrease in occupancy by *Mg*^2+ ^(*O_tmgmg_*).

## Discussion

It is well-established that mammalian cardiac excitation-contraction coupling is mediated by calcium-induced calcium release (CICR). We have developed a comprehensive mechanistic model of CICR under voltage clamp conditions in the rat ventricular myocyte, which includes electrical equivalent circuit models for both the free sarcolemma and that portion involving junctional transmission, as well as, fluid compartment models for several fluid media within the cell (dyadic cleft space, longitudinal sarcoplasmic reticulum (LSR or *Ca*^2+ ^uptake compartment), junctional sarcoplasmic reticulum (jSR or release compartment), and the cytosolic fluid compartment). An external bathing medium completes our fluid compartment description of the cell Figure 1. The multiple component model is referred to as the "whole-cell model" (Figures 1 and 2). We have probed the mechanisms regulating *Ca*^2+ ^in the cell. In particular, we have focused on the dyadic mechanisms effecting CICR.

The dyadic controller is a finely tuned coupling device consisting of two opposed *Ca*^2+ ^channels separated by a small dyadic space (Figure 1B): the sarcolemmal DHP-sensitive "trigger" channel and the Ry-sensitive jSR *Ca*^2+ ^release channel. The trigger channel is voltage-activated and is driven in our simulations by a voltage clamp pulse which opens the channels, admitting *Ca*^2+ ^influx (trigger current) to the dyadic space. After diffusion within the dyadic space, trigger *Ca*^2+ ^affects the *Ca*^2+ ^dependent open probability of the jSR *Ca*^2+ ^release channel over a small range of *Ca*^2+ ^concentrations. Clamp voltage magnitude strongly affects the *I_Ca,L _*current and hence the amount of *Ca*^2+ ^delivered to the dyadic space, but the range over which the *Ca*^2+ ^concentration at the mouth of the *Ca*^2+ ^release channel changes (in the absence of RyR release) is quite small (23.42*μ*M to 8.82*μ*M for a change in VC from 10 mv to 40 mv). The RyR-mediated *Ca*^2+ ^release is activated by trigger current, but the release itself is affected by the concentration gradient between the jSR and the dyadic space, and the temporal and refractory properties of the ryanodine receptor (Figure 13). The four-state RyR model is informed regarding supply *Ca*^2+ ^content by the jSR luminal sensor, a novel feature in our model which characterizes the important protein-protein interactions between calsequesterin, triadin/junctin and the RyR receptor. Triadin/junctin strongly regulates the sensitivity of RyR to trigger *Ca*^2+^. Hence the luminal sensor, which is a key element responsible for robust post-release RyR inactivation and refractoriness of the Ry-sensitive *Ca*^2+ ^release channel, is critical in providing realistic fits to cytosolic *Ca*^2+ ^transients and an adequate refilling time for the SR *Ca*^2+ ^stores. *Ca*^2+ ^release is thus graded with *Ca*^2+ ^concentration at the mouth of *Ca*^2+ ^channel in a very sensitive manner with a gain of approximately 7, as is shown in Figure 12.

The sarcolemmal portion of the dyadic membrane defining the dyadic space contains voltage-sensitive *Ca*^2+ ^channels and deals with changes in the external environment of the ventricular cell (e.g., membrane response to changes in transmembrane potential and chemical signaling agents). It integrates these various stimuli and delivers a trigger current to the small dyadic space. In contrast, the jSR membrane lining the opposite boundary of the dyadic space is concerned with the adequacy of *Ca*^2+ ^release in CICR. It contains Ry-sensitive *Ca*^2+ ^channels that require a *Ca*^2+ ^concentration gradient directed across the channel and into the dyadic space for their operation. Thus, jSR *Ca*^2+ ^concentration must be maintained within an acceptable range so that calcium is always available for ready release. The relationship between jSR *Ca*^2+ ^content and peak [*Ca*^2+^]*_myo _*is shown in Figure 16A. The LSR compartment connects the jSR compartment with the cytosolic compartment. It feeds make-up calcium to the jSR, by using a SERCA pump to actively draw in *Ca*^2+ ^from the myoplasm. Pumping rate is controlled by the cytosolic *Ca*^2+ ^concentration [*Ca*^2+^]*_myo _*as well as the LSR *Ca*^2+ ^concentration [*Ca*^2+^]*_LSR _*as part of a mechanism for replenishing and maintaining jSR *Ca*^2+ ^stores. Our model also incorporates the *Ca*^2+ ^induced CaM mediated effects of CaMKII and CaN on targets such as the DHP-sensitive *I_Ca,L _*channel, Ry-sensitive *Ca*^2+ ^release channel, as well as the SR *Ca*^2+ ^ATPase pump. Provision for the effects of phospholamban on SERCA has also been included.

The voltage-gated and chemically gated channels of the dyad are tightly coupled by feedback mechanisms that involve *Ca*^2+ ^signaling. Although physically separated, the voltage-sensitive *Ca*^2+ ^channel (Figure 9) as well as the Ry-sensitive release channel (Phase C in Figure 15) are inhibited by increased *Ca*^2+ ^levels in the dyadic space [53]. In earlier models which lacked the luminal sensor, RyR self-inhibition by dyadic *Ca*^2+ ^was the only mechanism besides stochastic attrition (both of which are inadequate in effecting a robust RyR channel closure) that was given the role of initiating RyR recovery. This has been tested in our model by artificially clamping all the variables (including concentration at the mouth of the RyR channel) to the levels reached at T2 in Figure 15 despite which the RyR open probability, begins to decline in phase C of Figure 15 showing the self-inhibitory role of high dyadic *Ca*^2+ ^concentration. Our model predicts (data not shown) that this *Ca*^2+ ^signaling continues even in the presence of high concentrations of *Ca*^2+ ^buffering agents in the cytosol (in agreement with the data of Adachi-Akahane et al. [71] and Diaz et al. [110]). Tight coupling between the DHP and Ry-sensitive *Ca*^2+ ^channels within the dyadic space thus preserves the mechanism of CICR under these extreme conditions.

Regulation of cytosolic *Ca*^2+ ^concentration [*Ca*^2+^]*_myo _*is evident at the cellular level. It is affected strongly by the voltage and [*Ca*^2+^]*_myo_*-dependent properties of the sarcolemmal currents *I_NaCa _*and *I_Ca,L_*, as well as the [*Ca*^2+^]*_myo_*-dependent properties of the *I_PMCA _*and SERCA pumps. We demonstrate a model-generated whole-cell *Ca*^2+ ^balance, which shows the importance of the *Na*^+^/*Ca*^+ ^exchanger in extruding the *Ca*^2+ ^that has entered the cell under normal activity, and also any excess that might occur when cytosolic *Ca*^2+ ^levels rise. The variety of experiments emulated in this study demonstrates quantification of *Ca*^2+ ^balances for all external and internal *Ca*^2+ ^fluxes and shows that the model has long-term stability in regulating cytosolic *Ca*^2+^, as shown in the 120-sec duration experiments of Negretti et al. (Figure 20) at a pulse repetition rate of 0.33 Hz., and the faster paced stimulation at 4 Hz shown in Figure 14A.

We have examined the dyadic component of the *I_Ca,L _*with regard to its *Ca*^2+ ^-dependent inactivation as a function of jSR *Ca*^2+ ^content (Figure 17C). Trigger current is voltage-dependent and therefore parameterized by clamp pulse amplitude (Figure 7A). In addition, its inactivation is *Ca*^2+ ^dependent, especially during *Ca*^2+ ^release. At a constant depolarizing voltage pulse, the pre-release jSR *Ca*^2+ ^content dictates the peak of the cytosolic *Ca*^2+ ^transient (Figure 16A), by controlling peak *Ca*^2+ ^concentration in individual dyads. As jSR *Ca*^2+ ^content increases, so does the peak dyadic *Ca*^2+ ^concentration, and the amount of sarcolemmal *Ca*^2+ ^influx declines due to greater *Ca*^2+ ^dependent inactivation. This autoregulatory feedback mechanism helps to establish a stable operating point for jSR *Ca*^2+ ^content. Transient *increases *in jSR *Ca*^2+ ^content bring about increases in *Ca*^2+ ^release, but reflexly, decrease sarcolemmal *Ca*^2+ ^influx via *I_Ca,L_*. Coupled with other dyadic and extra-dyadic mechanisms (*I_NaCa_*) that decrease [*Ca*^2+^]*_myo _*and hence *Ca*^2+^-uptake via the SERCA pump, jSR *Ca*^2+ ^content decreases. For *decreases *in jSR *Ca*^2+ ^content, the opposite occurs, so that regardless of the sign of the perturbation in jSR *Ca*^2+ ^content, it tends to stay constant in the steady-state. This is an important feature of the dyadic mechanism that preserves the integrity of CICR. The gain of this feedback system about the operating point is visualized as the slope of the peak *Ca*^2+ ^transient vs SR *Ca*^2+ ^content characteristic (Figure 16A) for a given voltage pulse level. Observing this figure, we note that there is a linear operating range beyond which the system gain increases dramatically. Our model results also show that a decrease of jSR *Ca*^2+ ^content (simulated either by a decrease of *Ca*^2+ ^uptake into the LSR by thapsigargin or an increase of *Ca*^2+ ^leak out of the jSR by caffeine) decreases systolic [*Ca*^2+^]*_myo_*, and hence the model might serve as a useful adjunct in a study of heart failure, where decreased contractility as a result of diminished *Ca*^2+ ^transients are commonly observed [114].

### Model Limitations

(a) This model of a rat ventricular myocyte is limited to *Ca*^2+ ^related channel, exchanger and pumps (*I_Ca,L_*, *I_NaCa_*, *I_PMCA _*and SERCA pump), while lacking exclusive *Na*^+ ^or *K*^+ ^related channels and transporters and is based on data at positive potentials in the range 10 mV ≤ V ≤ 40 mV. It is aimed at mimicking voltage clamp conditions where channels other than calcium are blocked, and it cannot be used to study experiments involving the generation of action potentials. However, its focus on the *Ca*^2+ ^dynamics allows one to comprehend more clearly the important role of *Ca*^2+ ^signalling pathways and feedback control systems in maintaining whole cell homeostasis over a prolonged period of time.

(b) A single dyadic space in our model has one representative, lumped DHP-sensitive and Ry-sensitive *Ca*^2+ ^channel, on opposing sarcolemmal and SR surfaces respectively. This simplified configuration does not conform to detailed structural information regarding the geometrical relationships between DHP and RyR-sensitive *Ca*^2+ ^channels [115,55,116] and thus cannot be used to draw conclusions about this part of the EC coupling process. However, our lumped abstraction forms a functional model of the dyadic coupling unit that can produce accurate predictions of cytosolic *Ca*^2+ ^transients. The effectiveness of this model is further demonstrated by its ability to accurately characterize the interaction between the DHP and Ry-sensitive *Ca*^2+ ^channels, including pulse duration dependent termination of release (Figure 11), *Ca*^2+ ^dependent inactivation of *I_Ca,L _*(Figures 7, 9), as well as the wide variety of whole-cell voltage clamp protocols (Figures 17, 18, 19 and 20).

(c) Although our model provides secondary *Ca*^2+ ^tail transients elicited by *I_Ca,L _*"tail currents" (Figure 21), this aspect of the model has not been verified extensively due to paucity of measured VC data showing tail transients over the high voltage range (40 ≤ V ≤ 60) in rat ventricular myocytes. Our *I_Ca,L _*tail currents are in general agreement with model generated data at a clamp voltage of 50 mV reported by Greenstein et al. ([8]; Figure 8). The restitution time for the RyR channel in rat ventricular myocytes is believed to be at least 25 ms [117] and as fast as 150 ms [118] indicating the wide range of values reported in the literature. We demonstrate model-generated RyR refractoriness by changing the duration of the simulated VC pulse and obtain RyR recovery characteristics that are consistent with measured data for *Ca*^2+ ^spark restitution in rat ventricular myocytes reported by Sobie et al. [106]. However, our results on this important phenomena, particularly the onset of tail transients with increasing inter-stimuli interval are preliminary and further modeling investigations would benefit considerably from the availability of additional measured data.

## Conclusion

We have developed a mathematical model of *Ca*^2+ ^dynamics under voltage clamp conditions in the rat ventricular myocyte, which is based solidly on experimental data and includes the most extensive description available of a novel feature, namely the luminal *Ca*^2+ ^sensor in the junctional SR which models the protein-protein interaction between triadin/junctin, calsequestrin and the RyR channel. The luminal sensor imparts the much needed refractoriness to the Ry-sensitive *Ca*^2+ ^release channel. This element is critical in providing realistic fits to cytosolic *Ca*^2+ ^transients and an adequate refilling time for the SR *Ca*^2+ ^stores. Our voltage-clamp simulations demonstrate graded *Ca*^2+ ^transients with sufficient gain, as well as quantification of *Ca*^2+ ^balances for all external and internal *Ca*^2+ ^fluxes. Our model of the dyadic coupling unit (DCU) provides mechanistic explanations of the major input-output relationship for CICR (Figure 16), as well as its modulation by trigger current (clamp voltage). The variety of experiments emulated in this study demonstrates that the model has long-term stability in regulating cytosolic *Ca*^2+^, as shown in the 120-sec duration experiments of Negretti et al. (Figure 20) at a pulse repetition rate of 0.33 Hz., and the faster (physiological) paced stimulation at 4 Hz shown in Figure 14A. It also provides biophysically based insights into the molecular mechanisms underlying whole-cell responses to the wide variety of testing approaches used in voltage clamp studies of myocytes that have appeared in the literature over the past two decades (Figures 17, 18, 19 and 20). Thus, the model serves as a platform for the predictive modeling of VC investigations in a number of areas. These include new hypotheses with regards to the under-expression of triadin/junctin resulting in a malfunctioning luminal sensor, which could affect long-term calcium stability of the cell (Figure 14B), and/or changes in the refractoriness of the RyR *Ca*^2+ ^channel (Figures 13 and 21) affecting the integrity of CICR under a variety of conditions. These are fundamental issues that would benefit from a better mechanistic understanding of deranged calcium signalling in the rat ventricluar myocyte. This study is aimed at providing an initial step towards this goal.

## Abbreviations

ATP: Adenosine triphosphate; [*Ca*^2+^]: calcium ion concentration; [*Ca*^2+^]*_dhp _*: *Ca*^2+ ^concentration at the mouth of the DHP-sensitive *I_Ca,L _*channel; [*Ca*^2+^]*_dyad _*: spatial *Ca*^2+ ^concentration in the dyad; [*Ca*^2+^]*_jSR _*: luminal *Ca*^2+ ^concentration in the jSR; [*Ca*^2+^]*_LSR _*: *Ca*^2+ ^concentration in the LSR; [*Ca*^2+^]*_myo _*: cytosolic *Ca*^2+ ^concentration; [*Ca*^2+^]*_serca _*: *Ca*^2+ ^concentration buffered by the SERCA protein; [*Ca*^2+^]*_o _*: extracellular *Ca*^2+ ^concentration; [*Ca*^2+^]*_ryr _*: *Ca*^2+ ^concentration at the "mouth" of the RyR channel on the dyadic side; CaCM: *Ca*^2+ ^bound calmodulin; CaF3: *Ca*^2+ ^bound to the buffer Fluo3; CaM: calmodulin; CaMKII: *Ca*^2+^/calmodulin-dependent protein kinase II; *CaMKII_act _*: activated *Ca*^2+^/calmodulin-dependent protein kinase II; CaN: calcineurin; *C_a_N_act _*: activated calcineurin; CDF: calcium dependent facilitation; CDI: calcium dependent inactivation; CF: caffeine; CICR: calcium-induced calcium-release; CS: calsequestrin; [*Cs*^+^]*_myo _*: *Cs*^+ ^concentration in the cytosol; [*Cs*^+^]*_dyad _*: *Cs*^+ ^concentration in the dyadic space; DCU: dyadic coupling unit; DHP: dihydropyridine; DHPR: dihydropyridine receptor; E-C: excitation contraction; $EC_{50}^{bwd}$: affinity of backward *Ca*^2+ ^flux from LSR to cytosol; $EC_{50}^{fwd}$: affinity of forward *Ca*^2+ ^flux from cytosol to LSR; *I_Ca,L _*: L-type *Ca*^2+ ^current; *I_Ca,L,SL _*: sarcolemmal component of the *I_Ca,L _*channel current; *I_Ca,L,TT _*: *I_Ca,L _*channel facing the dyadic space; *I_Cs _*: cesium current through the *I_Ca,L _*channel; *I_cyt,serca _*: *Ca*^2+ ^uptake current directed from the cytosol to the SERCA; *I_Na _*: sodium current through the *I_Ca,L _*channel; *I_Na,b _*: background sodium current; *I_NaCa _*: sodium calcium exchanger current; *I_NaCa,SL _*: sarcolemmal component of the *I_NaCa _*exchanger current; *I_NaCa,TT _*: *I_NaCa _*exchanger facing the dyadic space; *I_NaCs _*: sodium cesium pump current; *I_PMCA _*: plasma membrane *Ca*^2+ ^ATPase pump current; *I_ryr _*: *Ca*^2+ ^current due to CICR from an individual jSR; *I_serca,sr _*: *Ca*^2+ ^uptake current directed from the SERCA to the LSR; *I_tr _*: *Ca*^2+ ^current due to concentration gradient driven *Ca*^2+ ^transport from LSR to jSR; jSR: junctional portion of the sarcoplasmic reticulum; *k_d _*: dissociation constant; *k_mp _*: half saturation constant for the sarcolemmal *Ca*^2+ ^pump; LCC: L-type DHP-sensitive *Ca*^2+ ^channel; L-type: long lasting type; LSR: longitudinal portion of the sarcoplasmic reticulum; mM: milli molar; mV: milli volt; [*Na*^+^]*_dyad _*: *Na*^+ ^concentration in the dyadic space; [*Na*^+^]*_myo _*: *Na*^+ ^concentration in the cytosol; nM: nano molar; *O_c _*: fractional occupancy of calmodulin by *Ca*^2+ ^in the cytosol; *O_calse _*: fractional occupancy of calsequestrin by *Ca*^2+ ^in the jSR; *O_tc _*: fractional occupancy of troponin-Ca sites by *Ca*^2+ ^in the cytosol; *O_tmgc _*: fractional ocupancy of troponin-Mg sites by *Ca*^2+ ^in the cytosol; *O_tmgmg _*: fractional occupancy of troponin-Mg sites by *Mg*^2+ ^in the cytosol; pC: pico coulomb; *P_o _*: Open probability; PKA: protein kinase A; *PLB_dp _*: Unphosphorylated phospholamban; *PLB_p _*: phosphorylated phospholamban; PSR: phospholamban to SERCA ratio; Ry: ryanodine; RyR: ryanodine receptor; SERCA: sarcoplasmic reticulum *Ca*^2+ ^ATPase; SL: sarcolemma; SR: sarcoplasmic reticulum; Tc: *Ca*^2+^-specific troponin binding site; T-tubule: transverse tubules; TT: transverse tubules; VC: voltage clamp; VDI: voltage dependent inactivation;

## Competing interests

The authors declare that they have no competing interests.

## Authors' contributions

AK carried out the voltage clamp modeling studies and drafted the manuscript. LS substantially contributed to the development of the rat ventricular cell model. MV made substantial intellectual contributions to the study and in drafting of the manuscript. PTP provided the experimental voltage clamp data used to validate the model and contributed to the drafting of the manuscript. JWC made key contributions to the conception and design, analysis and interpretation of data, and drafting of the manuscript. All authors read and approved the final manuscript.

## Appendix

Below is the complete set of equations used in the model.

### A1 - Equations for currents in the model

L-Type Ca^2+ ^current

*Ca*^2+ ^current through the DHP-sensitive *I*_*Ca*,*L *_channel

(1) $$\begin{array}{l} I_{Ca,L}=R_{Ca,L}(O2_{dhpr}+O3_{dhpr})P_{Ca}Z_{Ca}^{2}\frac{F^{2}V}{RT}\times \\ \left(\frac{{\left[Ca^{2+}\right]}_{dhp}e^{\frac{2FV}{RT}}-341.0{\left[Ca^{2+}\right]}_{0}}{e^{\frac{2FV}{RT}}-1}\right)\;\; \end{array}$$

*Na*^+ ^current through the DHP-sensitive *I*_*Ca*,*L *_channel

(2) $$\begin{array}{l} I_{Na}=\frac{1.056\times e^{\left(\frac{\nu -21.73}{21.23841}\right)}}{1.056\times e^{\left(\frac{\nu -21.73}{21.23841}\right)}+[Ca_{o}^{2+}]}P_{Na}Z_{Na}^{2}\frac{F^{2}V}{RT}\times \\ \left(\frac{0.75{\left[Na^{+}\right]}_{dhp}e^{\frac{FV}{RT}}-0.75{\left[Na^{+}\right]}_{0}}{e^{\frac{FV}{RT}}-1}\right) \end{array}$$

*Cs*^+ ^current through the DHP-sensitive *I*_*Ca*,*L *_channel

(3) $$\begin{array}{l} I_{Cs}=\frac{1.056\times e^{\left(\frac{\nu -21.73}{21.23841}\right)}}{1.056\times e^{\left(\frac{\nu -21.73}{21.23841}\right)}+[Ca_{o}^{2+}]}P_{Cs}Z_{Cs}^{2}\frac{F^{2}V}{RT}\times \\ \left(\frac{0.75{\left[Cs^{+}\right]}_{dhp}e^{\frac{FV}{RT}}-0.75{\left[Cs^{+}\right]}_{0}}{e^{\frac{FV}{k_{C\text{s}}RT}}-1}\right) \end{array}$$

where *k_Cs _*= 0.5 and [*Ca*^2+^]*_dhp_*, [*Na*^+^]*_dhp_*, [*Cs*^+^]*_dhp _*are Concentrations at the mouth of the DHP-sensitive *Ca*^2+ ^channel.

The corresponding unitary currents *i_Ca_*,*_L_*, *i_Na_*, *i_Cs _*are obtained by dividing the above net channel currents by the number of dyadic units, *N_dyad_*.

Gating scheme for the 2-state Markovian model used to allow *Ca*^2+ ^mediated interaction of the DHP-sensitive *I_Ca_*,*_L _*channel and calmodilin:

(4) $$S1_{dhpr}\;=\;1-S2_{dhpr}$$

(5) $$\frac{dS2_{dhpr}}{dt}\;=\;ks_{12}^{dhpr}S1_{dhpr}-ks_{21}^{dhpr}S2_{dhpr}$$

Expressions for the rate constants:

(6) $$ks_{12}^{dhpr}\;=\;4.0\times e^{\left(\frac{{\left[Ca_{4}CaM\right]}_{dhp}-100.0}{90.0}\right)}\;$$

(7) $$ks_{21}^{dhpr}=18.0$$

Gating scheme for the 6-state Markovian model for the DHP-sensitive L-type *Ca*^2+ ^release channel:

(8) $$\begin{array}{l} C5_{dhpr}=1-(C1_{dhpr}+O2_{dhpr}+O3_{dhpr})\\ -(C4_{dhpr}) \end{array}$$

(9) $$\begin{array}{l} \frac{dC1_{dhpr}}{dt}=k_{21}^{dhpr}O2_{dhpr}+k_{51}^{dhpr}C5_{dhpr}\\ -(k_{12}^{dhpr}+k_{15}^{dhpr})C5_{dhpr} \end{array}$$

(10) $$\begin{array}{l} \frac{dO2_{dhpr}}{dt}=k_{12}^{dhpr}C1_{dhpr}+k_{42}^{dhpr}C4_{dhpr}\\ +k_{32}^{dhpr}O3_{dhpr}+k_{52}^{dhpr}C5_{dhpr}\\ -(k_{21}^{dhpr}+k_{23}^{dhpr})\;O2_{dhpr}\\ -(k_{24}^{dhpr}+k_{25}^{dhpr})\;O2_{dhpr} \end{array}$$

(11) $$\frac{dO3_{dhpr}}{dt}=k_{23}^{dhpr}O2_{dhpr}-k_{32}^{dhpr}O3_{dhpr}$$

(12) $$\begin{array}{l} \frac{dC4_{dhpr}}{dt}=k_{54}^{dhpr}C5_{dhpr}+k_{24}^{dhpr}O2_{dhpr}\\ -(k_{45}^{dhpr}+k_{42}^{dhpr})\;C4_{dhpr} \end{array}$$

(13) $$\frac{dC6_{dhpr}}{dt}=k_{36}^{dhpr}O3_{dhpr}-k_{63}^{dhpr}C6_{dhpr}$$

The open state *O*3*_dhpr _*accounts for the increased tail current produced as the result of a large depolarization. Expressions for rate constants:

(14) $$k_{42}^{dhpr}=1000.0$$

(15) $$k_{45}^{dhpr}=600000.0$$

(16) $$k_{54}^{dhpr}\;=\;\frac{k_{45}^{dhpr}k_{52}^{dhpr}k_{24}^{dhpr}}{k_{42}^{dhpr}k_{25}^{dhpr}}$$

(17) $$k_{15}^{dhpr}\;=\;\frac{k_{51}^{dhpr}k_{12}^{dhpr}k_{25}^{dhpr}}{k_{52}^{dhpr}k_{21}^{dhpr}}$$

• **Case 1: **With CICR (control)

(18) $$k_{12}^{dhpr}=\left(300.3808-\frac{301.0817}{1+e^{\left(\frac{\nu -13.5918}{8.85}\right)}}\right)$$

(19) $$k_{21}^{dhpr}\;=\;\left(3359.8754+\frac{966.95}{1+e^{\left(\frac{\nu -1.66}{1.4585}\right)}}\right)$$

(20) $$\begin{array}{l} \xi =550+6\times \;CaMKII_{act}\\ \;\;+CaN_{act} \end{array}$$

(21) $$k_{24}^{dhpr}\;=\;\left(\frac{336160\times S2_{dhpr}}{\xi }\right)$$

(22) $$k_{25}^{dhpr}=\left(\frac{5939.4+\frac{306806.8}{1+e^{\left(\frac{\nu +1.8716}{1.3072}\right)}}}{\xi }\right)$$

(23) $$k_{52}^{dhpr}\;=\;\left(0.02925+\frac{0.48961}{1+e^{\left(\frac{\nu +12.2249}{0.974}\right)}}\right)$$

(24) $$k_{23}^{dhpr}\;=\;\left(1352-\frac{1350}{1+e^{\left(\frac{U}{1.8}\right)}}+\frac{1700}{1+e^{\left(v+5\right)}}\right)$$

(25) $$k_{32}^{dhpr}\;=\;\left(1030.0575-\frac{713.1966}{1+e^{\left(v-5.0\right)}}\right)\;\;$$

(26) $$\begin{array}{l} k_{51}^{dhpr}\;=\;\frac{383.5435}{1+e^{-\left(\frac{\nu -8.3702}{7.047}\right)}}+\frac{0.16725}{1+e^{-\left(\frac{\nu -31.8252}{0.01075}\right)}}\\ +\frac{378.7085}{1+e^{\left(\frac{\nu -7.8953}{6.7691}\right)}}-373.015\;\; \end{array}$$

(27) $$k_{36}^{dhpr}\;=\;\left(100-\frac{100.0}{1+e^{\left(\frac{\nu -41.0}{1.0}\right)}}\right)\;\;$$

(28) $$k_{63}^{dhpr}\;=\;\left(\frac{600.0}{1+e^{\left(\frac{\nu -41.0}{1.0}\right)}}\right)$$

• **Case 2: **With Ryanodine applied (no RyR release)

(29) $$k_{12}^{dhpr}=\left(30392.87-\frac{30394.73}{1+e^{\left(\frac{\nu -183.5975}{28.5662}\right)}}\right)$$

(30) $$k_{21}^{dhpr}\;=\;\left(3568.74658+\frac{21921.25344}{1+e^{\left(\frac{\nu +24.9838}{0.7372}\right)}}\right)\;\;$$

(31) $$\begin{array}{l} \xi \;=\;550+6\times CaMKII_{act}\\ +CaN_{act}\;\; \end{array}$$

(32) $$k_{24}^{dhpr}\;=\;\left(\frac{336160\times S2_{dhpr}}{\xi }\right)$$

(33) $$k_{25}^{dhpr}\;=\frac{0.0893+\frac{26.3268}{1+e^{\left(\frac{\nu +15.0}{1.0}\right)}}}{\xi }$$

(34) $$+\frac{\frac{32.6642}{1+e^{-\left(\frac{\nu -14.4897}{0.3131}\right)}}+\frac{63.808}{1+e^{\left(\frac{\nu -27.411}{0.0854}\right)}}}{\xi }\;\;$$

(35) $$\begin{array}{l} k_{52}^{dhpr}\;=\;2.69+\frac{0.74152}{1+e^{\left(\frac{\nu +27.945}{1.46976}\right)}}\\ -\frac{2.43098}{1+e^{\left(\frac{\nu -40.25}{0.4034}\right)}}\;\; \end{array}$$

(36) $$\begin{array}{l} k_{23}^{dhpr}\;=\;1908-\frac{2687.3087}{1+e^{\left(\frac{\nu +15.0}{0.9998}\right)}}\\ \text{+ }\frac{879.543}{1+e^{\left(\frac{\nu -11.1635}{0.3238}\right)}} \end{array}$$

(37) $$\begin{array}{l} k_{32}^{dhpr}\;=\;145107.50934-\frac{150299.048}{1+e^{\left(\frac{\nu -1037.5224}{312.91482}\right)}}\\ +\frac{5537.7875}{1+e^{\left(\frac{\nu -9.4649}{0.2893}\right)}}\; \end{array}$$

(38) $$\begin{array}{l} k_{51}^{dhpr}\;=\;\frac{27.651}{1+e^{-\left(\frac{\nu -59.48}{4.444}\right)}}+\frac{11.488}{1+e^{-\left(\frac{\nu +15.1148}{0.9423}\right)}}\\ -\frac{16.3777}{1+e^{-\left(\frac{\nu +15.01}{0.6351}\right)}}+5.3384\;\; \end{array}$$

(39) $$k_{36}^{dhpr}\;=\;\left(100-\frac{100.0}{1+e^{\left(\frac{\nu -41.0}{1.0}\right)}}\right)$$

(40) $$k_{63}^{dhpr}\;=\;\left(\frac{600.0}{1+e^{\left(\frac{\nu -41.0}{1.0}\right)}}\right)\;\;$$

• **Case 3: **With *Ca*^2+ ^substituted with *Ba*^2+^

(41) $$\begin{array}{l} k_{12}^{dhpr}\;=\;3028.7604-\frac{6279.833}{1+e^{\left(\frac{\nu -58.1484}{9.5348}\right)}}\\ +\frac{3259.706}{1+e^{\left(\frac{\nu -40.2856}{0.2272}\right)}} \end{array}$$

(42) $$\begin{array}{l} k_{21}^{dhpr}\;=\;\frac{26296.0826}{1+e^{\left(\frac{\nu +17.40398}{0.22797}\right)}}+\frac{4718.8152}{1+e^{-\left(\frac{\nu +1.4892}{4.9581}\right)}}\\ +\frac{1544.9745}{1+e^{-\left(\frac{\nu -132.7305}{3.6234}\right)}}-808.0319 \end{array}$$

(43) $$k_{24}^{dhpr}=(0.0)$$

(44) $$\begin{array}{l} k_{25}^{dhpr}\;=\;-5883.3476+\frac{5973.3014}{1+e^{\left(\frac{\nu -41.4221}{0.5703}\right)}}\\ \frac{528.171}{1+e^{-\left(\frac{\nu -20.4274}{0.187}\right)}}\;\; \end{array}$$

(45) $$\begin{array}{l} k_{52}^{dhpr}\;=\;175.78742-\frac{174.7872}{1+e^{\left(\frac{\nu -32.3072}{4.4783}\right)}}\\ -\frac{229.8479}{1+e^{-\left(\frac{\nu -36.9508}{5.4521}\right)}}\;\; \end{array}$$

(46) $$\begin{array}{l} k_{23}^{dhpr}\;=\;\frac{2601.9597}{1+e^{\left(\frac{\nu -19.882}{0.4459}\right)}}-\frac{2647.5114}{1+e^{\left(\frac{\nu +15.6872}{2.1947}\right)}}\\ -\frac{2501.6394}{1+e^{\left(\frac{\nu -39.8404}{0.3926}\right)}}+2647.2042\;\; \end{array}$$

(47) $$\begin{array}{l} k_{32}^{dhpr}\;=\;\frac{1683.7686}{1+e^{-\left(\frac{\nu -20.0452}{0.40326}\right)}}+\frac{2161.9699}{1+e^{\left(\frac{\nu -360.2862}{1879.441}\right)}}\\ -\frac{5926.5225}{1+e^{-\left(\frac{\nu +1.7842}{13.43183}\right)}}+4129.8882\;\; \end{array}$$

(48) $$\begin{array}{l} k_{51}^{dhpr}\;=\;\frac{11.1993}{1+e^{-\left(\frac{\nu -35.2478}{0.2466}\right)}}+\frac{36.2341}{1+e^{-\left(\frac{\nu +23.9615}{15.8653}\right)}}\\ -\frac{12.163}{1+e^{-\left(\frac{\nu -27.1529}{0.9455}\right)}}+37.7673\;\; \end{array}$$

(49) $$k_{36}^{dhpr}\;=\;\left(100-\frac{100.0}{1+e^{\left(\frac{\nu -41.0}{1.0}\right)}}\right)$$

(50) $$k_{63}^{dhpr}\;=\;\left(\frac{600.0}{1+e^{\left(\frac{\nu -41.0}{1.0}\right)}}\right)$$

### Uptake of *Ca*^2+ ^from the cytosol into the LSR

*Ca*^2+ ^fluxes from cytosol to SERCA and SERCA to LSR

(51) $$\begin{array}{l} J_{cyt,serca}\;=\;k_{cyt,serca}\times {\left[Ca^{2+}\right]}_{myo}^{2}\times SERCA_{tot}\\ -k_{cyt,serca}\times {\left[Ca^{2+}\right]}_{myo}^{2}\times {\left[Ca^{2+}\right]}_{serca}\\ -k_{serca,cyt}\times {\left[Ca^{2+}\right]}_{serca}\;\; \end{array}$$

(52) $$\begin{array}{l} J_{serca,sr}=k_{sr,serca}\times {\left[Ca^{2+}\right]}_{LSR}^{2}\times {\left[Ca^{2+}\right]}_{serca}\\ -k_{sr,serca}\times {\left[Ca^{2+}\right]}_{LSR}^{2}\times SERCA_{tot}\\ +k_{cyt,sr}\times {\left[Ca^{2+}\right]}_{myo}\;\; \end{array}$$

(53) $$I_{cyt,serca}\;=\;J_{cyt,serca}\times 2FV_{serca}$$

(54) $$I_{serca,sr}\;=\;J_{serca,sr}\times 2FV_{serca}$$

Differential equation for *Ca*^2+ ^buffered by the SERCA protein:

(55) $$\frac{d{\left[Ca^{2+}\right]}_{serca}}{dt}=J_{cyt,serca}-J_{serca,sr}$$

Expressions for the rate constants for *Ca*^2+ ^binding to/release from SERCA:

(56) $$k_{cyt,serca}\;=k_{cyt,serca}\;(1.07\;+5500\;\times CaMKII_{act})\;$$

(57) $$K_{cyt,serca}\;=K_{cyt,serca}\;{\left(\;EC_{50}^{fwd}\right)}^{2}\;\;$$

(58) $$k_{serca,sr}=K_{serca,sr}\;(1.07+5500\times CaMKII_{act})$$

(59) $$k_{sr,serca}=\frac{K_{serca,sr}}{{\left(EC_{50}^{bwd}\right)}^{2}}$$

Affinities for the forward and backward *Ca*^2+ ^fluxes:

(60) $$\begin{array}{l} EC_{50}^{fwd}\;=\;0.015(1+PSR\;\times PLB_{dp})\\ \;\times \left(\frac{1.027}{1+5500\times CaMKII_{act}}\right) \end{array}$$

(61) $$EC_{50}^{bwd}\;=\;1250-1110\times PSR\;\times PLB_{dp}$$

Differential equation for phospholamban

(62) $$\begin{array}{l} \frac{dPLB_{dp}}{dt}=k_{12}^{PLB}PLB_{p}\\ -k_{21}^{PLB}(5500CaMKII_{act}+\\ {2.5CaN_{act}\;+PKA_{act})}^{2}PLB_{dp}\; \end{array}$$

(63) $$PLB_{p}=1-PLB_{dp}$$

### Ca^2+ ^pump in SL

(64) $$I_{PMCA}=\bar{I_{PMCA}}\left(\frac{{\left[Ca^{2+}\right]}_{myo}}{kmpca+{\left[Ca^{2+}\right]}_{myo}}\right)$$

### Na^+^/Ca^2+ ^exchanger

(65) $$I_{NaCa}=\frac{NUM1\times (NUM2-NUM3)}{℘\left(1+0.27e^{{}^{\frac{-0.65FV}{RT}}}\right)}\;$$

(66) $$NUM1\;=\;R_{NaCa}\left(\frac{V_{\max}}{1+{\left(\frac{K_{mAllo}}{{\left[Ca^{2+}\right]}_{NaCa}}\right)}^{2}}\right)\;$$

(67) $$NUM2\;=\;e^{\frac{0.35FV}{RT}}{\left({\left[Na^{+}\right]}_{NaCa}\right)}^{3}{\left[Ca^{2+}\right]}_{o}$$

(68) $$NUM3\;=\;e^{\frac{0.65FV}{RT}}{\left({\left[Na^{+}\right]}_{o}\right)}^{3}{\left[Ca^{2+}\right]}_{NaCa}$$

(69) $$\begin{array}{l} ℘=K_{mCao}{\left({\left[Na^{+}\right]}_{NaCa}\right)}^{3}+K_{mNao}^{3}{\left[Ca^{2+}\right]}_{NaCa}\\ +K_{mNai}^{3}[Ca^{2+}]\left(1+\frac{{\left[Ca^{2+}\right]}_{NaCa}}{K_{mCai}}\right)\\ +K_{mCai}{\left({\left[Na^{+}\right]}_{o}\right)}^{3}\left(1+{\left(\frac{{\left[Na^{+}\right]}_{NaCa}}{K_{mNai}}\right)}^{3}\right)\\ +{\left({\left[Na^{+}\right]}_{NaCa}\right)}^{3}{\left[Ca^{2+}\right]}_{o}\\ +{\left({\left[Na^{+}\right]}_{o}\right)}^{3}{\left[Ca^{2+}\right]}_{NaCa} \end{array}$$

where [*Ca*^2+^]*_NaCa_*, [*Na*^+^]*_NaCa _*are Concentrations at the mouth of the NaCa-exchanger.

The corresponding unitary current *i_NaCa _*is obtained by dividing the above net channel current *I_NaCa _*by the number of dyadic units N*_dyad_*.

### Na^+^/Cs^+ ^pump

(70) $$\begin{array}{l} I_{NaCs}\;=\;R_{NaCs}\bar{I_{NaCs}}\left(\frac{{\left[Cs^{+}\right]}_{0}}{{\left[Cs^{+}\right]}_{0}+kmcs}\right)\\ \times \left(\frac{100{\left({\left[Na^{+}\right]}_{myo}\right)}^{1.5}}{{\left({\left[Na^{+}\right]}_{myo}\right)}^{1.5}+kmna^{1.5}}\right)\\ \times \left(\frac{0.65558}{0.18445+e^{-\left(\frac{\nu +53.353}{15.58}\right)}}\right) \end{array}$$

The corresponding unitary current *i_NaCs _*is obtained by dividing the above net channel current *I_NaCs _*by the number of dyadic units *N_dyad_*.

### Background Na^+ ^current

(71) $$I_{Na,b}=G_{Nab}\left(\nu -\frac{RT}{F}\ln\left(\frac{{\left[Na^{+}\right]}_{o}}{{\left[Na^{+}\right]}_{myo}}\right)\right)$$

### Ca^2+ ^transfer from LSR to a single jSR

(72) $$i_{tr}=\left(\frac{{\left[Ca^{2+}\right]}_{LSR}-{\left[Ca^{2+}\right]}_{jSR}}{\tau_{tr}}\right)2FV_{jSR}$$

### Ca^2+ ^release from a unit jSR into a single DCU

(73) $$i_{ryr}=J_{ryr}\times (2F\times \pi {△}_{r}^{2}{△}_{z})$$

where,

(74) $$J_{ryr}=\frac{O2_{ryr}({\left[Ca^{2+}\right]}_{jSR}-{\left[Ca^{2+}\right]}_{ryr})P_{ryr}}{\pi {△}_{r}^{2}{△}_{z}}$$

Gating scheme for the 4-state Markovian model for the RyR-sensitive SR *Ca*^2+ ^release channel:

(75) $$C4_{ryr}=1-(C1_{ryr}+O2_{ryr}+C3_{ryr})$$

(76) $$\begin{array}{l} \frac{dC1_{ryr}}{dt}=k_{41}^{ryr}C4_{ryr}+k_{21}^{ryr}O2_{ryr}\\ -(k_{12}^{ryr}+k_{14}^{ryr})C1_{ryr} \end{array}$$

(77) $$\begin{array}{l} \frac{dO2_{ryr}}{dt}=k_{12}^{ryr}C1_{ryr}+k_{32}^{ryr}C3_{ryr}\\ -(k_{21}^{ryr}+k_{23}^{ryr})O2_{ryr} \end{array}$$

(78) $$\begin{array}{l} \frac{dC3_{ryr}}{dt}=k_{23}^{ryr}O2_{ryr}+k_{43}^{ryr}C4_{ryr}\\ -(k_{34}^{ryr}+k_{32}^{ryr})C3_{ryr} \end{array}$$

(79) $$\begin{array}{l} k_{12}^{ryr}={\left[Ca^{2+}\right]}_{ryr}^{2}\left(0.05-\frac{3.7465\times 10^{-2}}{1+e^{\frac{{\left[Ca^{2+}\right]}_{ryr}-2052.7}{5.983}}}\right)var\\ +\left(\frac{CaMKII_{act}}{12000}\right) \end{array}$$

(80) $$\begin{array}{l} k_{21}^{ryr}=\left(698.56-\frac{618.56}{1+e^{\frac{{\left[ca^{2+}\right]}_{ryr}-2052.9}{4.35}}}\right)\\ \times \left(1+\frac{6.0}{{\left[Ca^{2+}\right]}_{ryr}}\right)\frac{1}{var} \end{array}$$

(81) $$k_{14}^{ryr}\;=\;8.748\times 10^{-2}{\left[Ca^{2+}\right]}_{ryr}var$$

(82) $$k_{41}^{ryr}\;=\;\left(\frac{2.0}{var}\right)+\frac{CaMKII_{act}}{12000}$$

(83) $$k_{43}^{ryr}\;=\;k_{12}^{ryr}\;;\;k_{34}^{ryr}=k_{21}^{ryr}\;;$$

(84) $$k_{23}^{ryr}\;=\;k_{14}^{ryr}\;;\;k_{32}^{ryr}=k_{41}^{ryr}\;;$$

(85) $$var\;=\;{\left[10.0\times e^{\frac{A1_{l\text{s}}-0.7013}{0.03}}\right]}^{2}\;\;$$

where [*Ca*^2+^]*_ryr _*is the *Ca*^2+ ^concentration at the mouth of the RyR channel on the dyadic side. In the presence of caffeine (CF, concentration in *μ*M):

(86) $$O2_{ryr}=\left(\frac{-0.522}{1+e^{\frac{[CF]-410}{264.9}}}\right)+0.503$$

Gating scheme for the 6-state Markovian model for the Luminal Calcium sensor.

(87) $$I4_{ls}\;=\;1-(A1_{ls}+I2_{ls}+I3_{ls})\;$$

(88) $$B5_{ls}\;=\;1-(I\;2_{ls}+I3_{ls}+B6_{ls})$$

(89) $$\frac{dA1_{ls}}{dt}=k_{41}^{ls}I4_{ls}+k_{21}^{ls}I2_{ls}-(k_{12}^{ls}B5_{ls}+k_{14}^{ls})A1_{ls}$$

(90) $$\begin{array}{l} \frac{dI2_{ls}}{dt}=k_{12}^{ls}B5_{ls}A1_{ls}+k_{42}^{ls}B5_{ls}I4_{ls}+k_{32}^{ls}I3_{ls}\\ -(k_{21}^{ls}+k_{24}^{ls}+k_{23}^{ls}+k_{25}^{ls})\;I2_{ls} \end{array}$$

(91) $$\frac{dI3_{ls}}{dt}=k_{43}^{ls}B5_{ls}I4_{ls}+k_{23}^{ls}I2_{ls}-(k_{34}^{ls}+k_{32}^{ls})\;I3_{ls}$$

(92) $$\frac{dB6_{ls}}{dt}=k_{56}^{ls}{\left[Ca^{2+}\right]}_{jSR}B5_{ls}-k_{65}^{ls}B6_{ls}$$

*A*1*_ls_*   Fractional occupancy of RyR by Triadin/Junctin

*I*2*_ls_*   Fractional occupancy of RyR by Triadin/Junctin and Calsequestrin

*I*3*_ls_*   Fractional occupancy of Triadin/Junctin by Calsequestrin

*I*4*_ls_*   Free Triadin/Junctin, B5ls - Free Calsequestrin

*B*6*_ls_*   Fractional Occupancy of Calsequestrin by Calcium

### Ca^2+ ^dependent CaM mediated activation of CaMKII and CaN

*Ca*^2+ ^binding to CaM and CaM buffering

(93) $$\begin{array}{l} CaM=CaM_{tot}\;-Ca_{2}CaM\;-Ca_{4}CaM\;-CaMB\\ -Ca_{2}CaMB\;-Ca_{4}CaMB\;-CaMCaN\\ -Ca_{2}CaMCaN\;-Ca_{4}CaMCaN\\ -CaMKII_{tot}\;(P_{1}+P_{3}+P_{5}+P_{4}) \end{array}$$

(94) $$B=B_{tot}-\;(CaMB\;+Ca_{2}CaMB\;+Ca_{4}CaMB)$$

(95) $$RO2=k_{02}^{CM}{\left[Ca^{2+}\right]}^{2}\times CaM-k_{02}^{CM}\times Ca_{2}CaM$$

(96) $$R24=k_{24}^{CM}{\left[Ca^{2+}\right]}^{2}\times Ca_{2}CaM-k_{42}^{CM}\times Ca_{4}CaM$$

(97) $$RO2B=k_{02B}^{CM}{\left[Ca^{2+}\right]}^{2}\times CaMB-k_{20B}^{CM}\times Ca_{2}CaMB$$

(98) $$\begin{array}{l} R24B=k_{24B}^{CM}{\left[Ca^{2+}\right]}^{2}\times Ca_{2}CaMB\\ -k_{42B}^{CM}\times Ca_{4}CaMB \end{array}$$

(99) $$ROB=k_{0Bon}^{CM}\times CaM\times B-k_{0Boff}^{CM}\times Ca_{2}CaMB$$

(100) $$R2B=k_{2Bon}^{CM}\times Ca_{2}CaM\times B-k_{2Boff}^{CM}\times Ca_{2}CaMB$$

(101) $$R4B=k_{4Bon}^{CM}\times Ca_{4}CaM\times B-k_{4Boff}^{CM}\times Ca_{4}CaMB$$

(102) $$\begin{array}{l} \frac{dCa_{2}CaM}{dt}=R02-R24-R2B-R2CaN\\ +CaMKII_{tot}\times (RCK_{56}-RCK_{21}) \end{array}$$

(103) $$\begin{array}{l} \frac{dCa_{4}CaM}{dt}=R24-R4CaN-R4B\\ \;-CaMKII_{tot}\times (RCK_{46}-RCK_{23}) \end{array}$$

(104) $$\begin{array}{l} \frac{dCa_{4}CaM}{dt}=R24-R4CaN-R4B\\ -CaMKII_{tot}\times (RCK_{46}-RCK_{23}) \end{array}$$

(105) $$\frac{dCaMB}{dt}=R0B-R02B$$

(106) $$\frac{dCa_{2}CaMB}{dt}=\;R02B+R2B-R24B$$

(107) $$\frac{dCa_{4}CaMB}{dt}=\;R24B-R4B\;\;$$

CaMKII activation:

(108) $$P_{2}\;=\;1-P_{3}-P_{1}-P_{4}-P_{5}-P_{6}$$

(109) $$T\;=\;P_{3}+P_{4}+P_{5}+P_{6}$$

(110) $$\begin{array}{l} k_{34}^{CK}\;=\;0.055\times T+0.0074\times T^{2}\\ +0.015\times T^{3}\;\; \end{array}$$

(111) $$\begin{array}{l} RCK_{34}=k_{34}^{CK}\times P_{3}\\ -\left(\frac{K_{PP1}\times PP1_{tot}\times P_{4}}{k_{mpp1}+(CaMKII_{tot}\times P_{4})}\right)\;\; \end{array}$$

(112) $$\begin{array}{l} RCK_{21}\;=\;k_{21}^{CK}\times Ca_{2}CaM\;\times P_{2}\\ -k_{12}^{CK}\times P_{1}\;\; \end{array}$$

(113) $$\begin{array}{l} RCK_{13}\;=\;k_{13}^{CK}\times {\left[Ca^{2+}\right]}^{2}\times P_{1}\\ -k_{32}^{CK}\times P_{3}\;\; \end{array}$$

(114) $$\begin{array}{l} RCK_{23}\;=\;k_{23}^{CK}\times Ca_{4}CaM\;\times P_{2}\\ \;-k_{32}^{CK}\times P_{3}\;\; \end{array}$$

(115) $$\begin{array}{l} RCK_{45}\;=\;k_{45}^{CK}\times P_{4}\\ -k_{54}^{CK}\times {\left[Ca^{2+}\right]}^{2}\times P_{5}\;\; \end{array}$$

(116) $$\begin{array}{l} RCK_{46}\;=\;k_{46}^{CK}\times P_{4}\\ -k_{64}^{CK}\times Ca_{4}CaM\;\times P_{6}\;\; \end{array}$$

(117) $$\begin{array}{l} RCK_{56}\;=\;k_{56}^{CK}\times P_{5}\\ -k_{65}^{CK}\times Ca_{2}CaM\;\times P_{6} \end{array}$$

(118) $$RCK_{51}\;=\;\frac{k_{PP1}\times PP1_{tot}\times P_{5}}{k_{mPP1}+(CaMKII_{tot}\times P_{5})}\;$$

(119) $$RCK_{62}\;=\;\frac{k_{PP1}\times PP1_{tot}\times P_{6}}{k_{mPP1}+(CaMKII_{tot}\times P_{6})}$$

(120) $$\frac{dP_{1}}{dt}=RCK_{21}+RCK_{51}-RCK_{13}$$

(121) $$\frac{dP_{3}}{dt}=RCK_{23}+RCK_{13}-RCK_{34}$$

(122) $$\frac{dP_{4}}{dt}=RCK_{34}-RCK_{46}-RCK_{45}$$

(123) $$\frac{dP_{5}}{dt}=RCK_{45}-RCK_{56}-RCK_{51}$$

(124) $$\frac{dP_{6}}{dt}=RCK_{46}-RCK_{56}-RCK_{62}$$

(125) $$CaMKII_{act}=100\times (P_{3}+P_{4}+P_{5}+P_{6})$$

CaN activation:

(126) $$\begin{array}{l} Ca_{2}CaN=CaN_{tot}\;-Ca_{4}CaN-CaMCaN\\ \;-Ca_{2}CaMCaN\;-Ca_{4}CaMCaN \end{array}$$

(127) $$\begin{array}{l} RCN_{Ca4}\;=k_{Caon}^{CN}\times {\left[Ca^{2+}\right]}^{2}\times Ca_{2}CaN\\ \;-k_{Caoff}^{CN}\times Ca_{4}CaN \end{array}$$

(128) $$\begin{array}{l} RCN_{02}=k_{02}^{CN}\times {\left[Ca^{2+}\right]}^{2}\times CaMCaN\\ -k_{20}^{CN}\times Ca_{2}CaMCaN \end{array}$$

(129) $$\begin{array}{l} RCN_{24}=k_{24}^{CN}\times {\left[Ca^{2+}\right]}^{2}\times Ca_{2}CaMCaN\\ -k_{42}^{CN}\times Ca_{4}CaMCaN \end{array}$$

(130) $$\begin{array}{l} RCN_{0}=k_{0on}^{CN}\times CaM\;\times Ca_{4}CaN\\ -k_{0off}^{CN}\times CaMCaN \end{array}$$

(131) $$\begin{array}{l} RCN_{2}=k_{2on}^{CN}\times Ca_{2}CaM\;\times Ca_{4}CaN\\ -k_{2off}^{CN}\times Ca_{2}CaMCaN \end{array}$$

(132) $$\begin{array}{l} RCN_{4}=k_{4on}^{CN}\times Ca_{4}CaM\;\times Ca_{4}CaN\\ -k_{4off}^{CN}\times Ca_{4}CaMCaN \end{array}$$

(133) $$\frac{dCa_{4}CaN}{dt}=RCN_{Ca4}-RCN_{0}-RCN_{2}-RCN_{4}$$

(134) $$\frac{dCaMCaN}{dt}=RCN_{0}-RCN_{02}$$

(135) $$\frac{dCa_{2}CaMCaN}{dt}=RCN_{2}+RCN_{02}-RCN_{24}$$

(136) $$\frac{dCa_{4}CaMCaN}{dt}=RCN_{4}+RCN_{24}$$

(137) $$\begin{array}{l} CaN_{act}=100(Ca_{4}CaMCaN\;+0.1\;Ca_{2}CaMCaN)\\ \text{+}10\;Ca_{4}CaMCaN\;+0.1\;CaMCaN\\ \text{+0}\text{.1}Ca_{\text{4}}CaN \end{array}$$

### A2 - Differential equations for buffers used in the model

Fluorescent indicator dye

(138) $$\begin{array}{l} \frac{d[CaF3]}{dt}=k_{fluo3}^{+}[Ca_{myo}^{2+}]({\left[fluo3\right]}_{tot}\;-[CaF3])\\ -k_{fluo3}^{-}[CaF3] \end{array}$$

Intracellular *Ca*^2+ ^buffering:

calmodulin (bulkmyoplasm):

(139) $$\frac{dO_{c}}{dt}=200{\left[Ca^{2+}\right]}_{myo}(1-O_{c})-476.0\times O_{c}$$

Troponin

(Fractional occupancy of troponin-Ca complex by *Ca*^2+^):

(140) $$\frac{dO_{t}c}{dt}=78.4{\left[Ca^{2+}\right]}_{myo}(1-O_{tc})-392.0\times O_{tc}$$

(Fractional occupancy of troponin-Mg complex by *Ca*^2+^):

(141) $$\begin{array}{l} \frac{dO_{c}}{dt}=200{\left[Ca^{2+}\right]}_{myo}(1-O_{tmgc}-O_{tmgmg})\\ -6.6\times O_{tmgc} \end{array}$$

(Fractional occupancy of troponin-Mg complex by *Mg*^2+^):

(142) $$\begin{array}{l} \frac{dO_{tmgmg}}{dt}=2.0{\left[Mg^{2+}\right]}_{myo}(1-O_{tmgc}-O_{tmgmg})\\ -666.0\times O_{tmgmg} \end{array}$$

### A3 - Differential equations for ion concentrations used in the model

Intracellular Ca^2+ ^concentration:

1. *Ca^2+ ^concentration in the cytosol*

(143) $$\begin{array}{l} \frac{d{\left[Ca^{2+}\right]}_{myo}}{dt}=\frac{I_{dyad}-I_{PMCA}-I_{Ca,L,SL}}{2FV_{myo}}\\ +\frac{-I_{cyt,serca}+2I_{NaCa,SL}}{2FV_{myo}}\\ -\frac{dO}{dt}-\frac{dCaF1}{dt} \end{array}$$

where *I_dyad _*is the net integrated *Ca*^2+ ^flux diffusing out of all the dyadic units into the cytosol

(144) $$\frac{dO}{dt}=3.2\frac{dO_{TC}}{dt}+6.4\frac{dO_{TMgC}}{dt}+1.8\frac{dO_{c}}{dt}$$

2. *Ca^2+ ^concentration in the jSR*

(145) $$\frac{d{\left[Ca^{2+}\right]}_{jSR}}{dt}=\frac{i_{tr}-i_{ryr}}{2FV_{jSR}}-31000\frac{dB6_{ls}}{dt}$$

3. Ca^2+ ^concentration in the LSR

(146) $$\frac{d{\left[Ca^{2+}\right]}_{LSR}}{dt}=\frac{I_{cyt,serca}-N_{dyad}i_{tr}}{2FV_{LSR}}$$

4. Diffusion equation for Calcium in the dyadic space

(147) $$\begin{array}{l} \frac{\partial {\left[Ca^{2+}\right]}_{dyad}}{\partial t}=D_{Ca}\nabla^{2}{\left[Ca^{2+}\right]}_{dyad}+J_{ryr}\\ \text{+}J_{dhpr}\text{+}J_{NaCa}\text{+}J_{bnd} \end{array}$$

(148) $$J_{dhpr}=\left(\frac{i_{Ca,L,TT}}{N_{dyad}}\right)\left(\frac{5.1821\times 10^{3}}{\pi {△}_{r}^{2}{△}_{z}}\right)$$

(149) $$J_{NaCa,TT}=\left(\frac{i_{NaCa,TT}}{N_{dyad}}\right)\left(\frac{2\times 5.1821\times 10^{3}}{\pi {△}_{r}^{2}{△}_{z}}\right)$$

(150) $$\begin{array}{l} J_{bnd}=\left(\frac{N_{h}K_{h}}{{\left(K_{h}+{\left[Ca^{2+}\right]}_{dyad}\right)}^{2}}\right)\\ \times \left(\frac{N_{l}K_{l}}{{\left(K_{l}+{\left[Ca^{2+}\right]}_{dyad}\right)}^{2}}\right)\frac{\partial {\left[Ca^{2+}\right]}_{dyad}}{\partial t} \end{array}$$

Intracellular Na^+ ^concentration:

1. *Na^+ ^concentration in the cytosol*

(151) $$\begin{array}{l} \frac{d{\left[Na^{+}\right]}_{myo}}{dt}=N_{dyad}\left[\frac{{\left[Na^{+}\right]}_{dyad}-{\left[Na^{+}\right]}_{myo}}{\tau_{Na}}\right]\\ -\left[\frac{I_{Na,b}+3I_{NaCa,SL}}{F\times V_{myo}}\right]\\ +\left[\frac{3I_{NaCs,SL}}{F\times V_{myo}}\right] \end{array}$$

2. *Na^+ ^concentration in the dyadic space*

(152) $$\begin{array}{l} \frac{d{\left[Na^{+}\right]}_{dyad}}{dt}=\left(\frac{{\left[Na^{+}\right]}_{myo}-{\left[Na^{+}\right]}_{dyad}}{\tau_{Na}}\right)\\ -\left(\frac{3i_{NaCa,TT}+3i_{NaCs,TT}}{F\times V_{cleft}}\right) \end{array}$$

Intracellular Cs^+ ^concentration:

1. *Cs^+ ^concentration in the cytosol*

(153) $$\begin{array}{l} \frac{d{\left[Cs^{+}\right]}_{myo}}{dt}=N_{dyad}\left(\frac{{\left[Cs^{+}\right]}_{dyad}-{\left[Cs^{+}\right]}_{myo}}{\tau_{Cs}}\right)\\ +\left(\frac{2I_{NaCs,SL}}{F\times V_{myo}}\right) \end{array}$$

2. *Cs^+ ^concentration in the dyadic space*

(154) $$\begin{array}{l} \frac{d{\left[Cs^{+}\right]}_{dyad}}{dt}=\left(\frac{{\left[Cs^{+}\right]}_{myo}-{\left[Cs^{+}\right]}_{dyad}}{\tau_{Cs}}\right)\\ +\left(\frac{2i_{NaCs,TT}}{F\times V_{cleft}}\right) \end{array}$$
